# Heuristically Adaptive Diffusion‐Model Evolutionary Strategy

**DOI:** 10.1002/advs.202511537

**Published:** 2026-03-07

**Authors:** Benedikt Hartl, Yanbo Zhang, Hananel Hazan, Michael Levin

**Affiliations:** ^1^ Allen Discovery Center at Tufts University Medford Massachusetts USA; ^2^ Institute for Theoretical Physics TU Wien Wien Austria; ^3^ Wyss Institute for Biologically Inspired Engineering at Harvard University Boston Massachusetts USA

**Keywords:** conditionally optimized, diffusion models, evolutionary algorithms, machine learning

## Abstract

Diffusion Models (DMs) and Evolutionary Algorithms (EAs) share a core generative principle: iterative refinement of random initial distributions to produce high‐quality solutions. DMs degrade and restore data using Gaussian noise, enabling versatile generation, while EAs optimize numerical parameters through biologically inspired heuristics. Our research integrates these frameworks, employing deep learning‐based DMs to enhance EAs across diverse domains. By iteratively refining DMs with heuristically curated databases, we generate better‐adapted offspring parameters, achieving efficient convergence toward high‐fitness solutions while preserving explorative diversity. DMs augment EAs with deep memory, retaining historical data and exploiting subtle correlations for refined sampling. Classifier‐free guidance further enables precise control over evolutionary dynamics, targeting specific genotypical, phenotypical, or population traits. This hybrid approach transforms EAs into adaptive, memory‐enhanced frameworks, offering unprecedented flexibility, and precision in evolutionary optimization, with broad implications for generative modeling and heuristic search.

## Graphical abstract

Building on the mathematical equivalence between diffusion models and evolutionary algorithms, researchers demonstrate unprecedented control over evolutionary optimization through conditional diffusion. By training diffusion models to associate parameters with specific traits, they can guide evolution toward solutions exhibiting desired behaviors independent of fitness scores, enabling multi‐objective optimization without complex reward engineering.

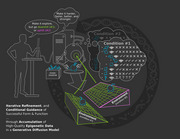

## 1 Teaser

Diffusion Models meet Evolutionary Algorithms: iterative refinement for conditionally optimized, diverse solutions with deep memory.

## 2 Introduction

Two fundamental mechanisms in the biosphere are known to drive novelty: evolution and learning. Conventionally, evolution is understood as a slow variational process adapting organisms or lineages across generations to changing environmental conditions through natural selection [1, 2]. In contrast, learning is a rapid transformational process enabling individuals to acquire knowledge and generalize based on subjective experiences in their lifetime [3, 4, 5, 6]. These mechanisms are extensively researched in separate domains of artificial intelligence, and recent studies have begun highlighting similarities between evolution and learning [7, 8, 9, 10, 11, 12, 13, 14, 15, 16].

Evolutionary algorithms (EAs) implement a search process using biologically inspired variational principles to iteratively refine sets of numerical parameters that encode potential solutions to often rugged, custom objective functions [17, 18, 19, 20]. Traditional black box EAs utilize heuristic population data and associated fitness scores (i.e., evaluations of the objective function reminiscent to the fitness of a phenotype in its environment) to sample new, potentially better adapted candidate solutions (i.e., offspring comprising the next generation). Depending on the specific implementation of the EA [21], this sampling process of novel genotypic parameters can be population‐based through recombination and mutation operations at the genotypic level of thereby iteratively adapted generations, or even leveraged by sampling novel data points from successively re‐parameterized probabilistic models, e.g., with a Gaussian prior [22] (c.f., Methods and Supporting Information Sections S1 and S2 for details).

Evolutionary algorithms incorporating generative processes operate on partial, heuristically‐derived knowledge of the fitness landscape. These algorithms iteratively train probabilistic models using progressively refined data to identify and exploit genotypic correlations across generations. Through continuous refinement of the generative model, they aim to increase the probability of sampling high‐fitness solutions, potentially accelerating optimization. While this approach offers computational advantages, it presents notable challenges. The use of parameterized models to learn the manifold of correlated genotypic parameters introduces an inductive bias that may constrain the evolutionary search. This learned structure could limit exploration of novel solutions and lead to premature convergence, potentially leaving promising regions of the search space unexplored. Moreover, it has become increasingly recognized that the evolution process does not simply build phenotypes adapted to specific environmental conditions [9, 23, 24, 25]: While this certainly is a by‐product, evolution seems to primarily bring forth problem solving machines on all scales in the biosphere. Following recent insights from developmental evolutionary biology [25, 26, 27, 28, 29], the genome does not represent a direct blueprint of all details of the mature organism, it rather instantiates a generative model to construct the latter: genes encode protein sequences which, when expressed in a cell, reconfigure and constrain the cell's functionality. This self‐orchestrated developmental process exhibits fundamental plasticity in both structure and function, enabling the substrate comprising an organism to adapt to novel internal and environmental stressors [25]. Such adaptive capability can be understood as a form of collective intelligence [24] aligning with William James' definition [30]: “Intelligence is the ability to reach the same goal by different means”. Our biosphere is organized as a multi‐scale competency architecture [25], which in turn has dramatic implications on the underlying evolutionary process [31, 32], which, however, is hardly aligned with current evolutionary search strategies; see the Supporting Information Section S1 for a discussion of promising approaches.

In contrast, recent breakthroughs in generative deep learning, particularly through diffusion models (DMs) [36, 37, 38, 39, 40], have lead to significant advances in artificial intelligence, so‐far predominantly in the image‐ or video generation domain [40, 41]. These models utilize stepwise iterative denoising to generate novel, realistic data points that conform to complex target data distributions. The impact of DMs extends significantly beyond visual content generation. In the field of computational biology, these models have advanced protein folding prediction [42]. Within optimization domains, DMs enable generative multi‐objective optimization through pretraining on closed datasets [43, 44, 45, 46]. Moreover, their application to generative game play through world modeling [47] demonstrates their versatility as a fundamental architecture for complex generative tasks across diverse domains.

Generative DMs iteratively transform Gaussian distributions into structured data points conforming to the training data distribution [48]. Drawing inspiration from dissipative systems of non‐equilibrium physics [39, 49], these models implement a two‐phase process: first, a forward (diffusion) process progressively corrupts data points with incremental noise; then, a model learns the reverse (generative) process to predict and remove this noise. This noise prediction enables iterative denoising during the generative process, where initially noisy samples are systematically refined until they match the statistical properties of the training data. The iterative denoising steps create a smooth trajectory through the latent space, allowing the model to capture complex, multi‐modal data distributions with remarkable fidelity (see Methods and the Supporting Information Section S2).

A key strength of DMs lies in their conditional training and sampling capabilities [33, 40], providing precise control over the generative output and thus offering unprecedented flexibility in directing the generation process toward desired outcomes. In general, DMs are tightly related to associative memory systems [50], which fundamentally enables these generative models to sample diverse yet high‐quality results on custom datasets while maintaining reliable training convergence.

The step‐wise error correction process inherent to DMs bears striking resemblance to the mechanisms observed in biology, particularly in their application to modeling multicellular growth and development [31]. This parallel suggests that DMs may be particularly well‐suited for simulating computationally irreducible [51] self‐orchestrated biological processes, including morphogenesis and evolution. The fundamental similarity in iterative refinement between DMs and biological development indicates shared underlying principles governing both artificial and natural generative processes, potentially offering new insights into the nature of generative systems across different domains.

In our complementary work [48], we established the mathematical equivalence between successive adaptation in evolutionary processes and the generative mechanisms of DMs. This equivalence emerges from a fundamental similarity in their iterative refinement processes: evolutionary systems combine directed selection with random mutations, while DMs balance progressive denoising with stochastic perturbations. This approach achieves performance competitive with mainstream methods, notably without deploying deep learning architectures, and illuminating a broader theoretical connection: DMs can serve as a crucial bridge between evolutionary and developmental biology concepts and contemporary machine learning approaches. This bidirectional relationship opens novel pathways for incorporating biological principles into artificial intelligence systems and, conversely, provides new computational frameworks for understanding evolutionary and developmental biology.

**FIGURE 1:**
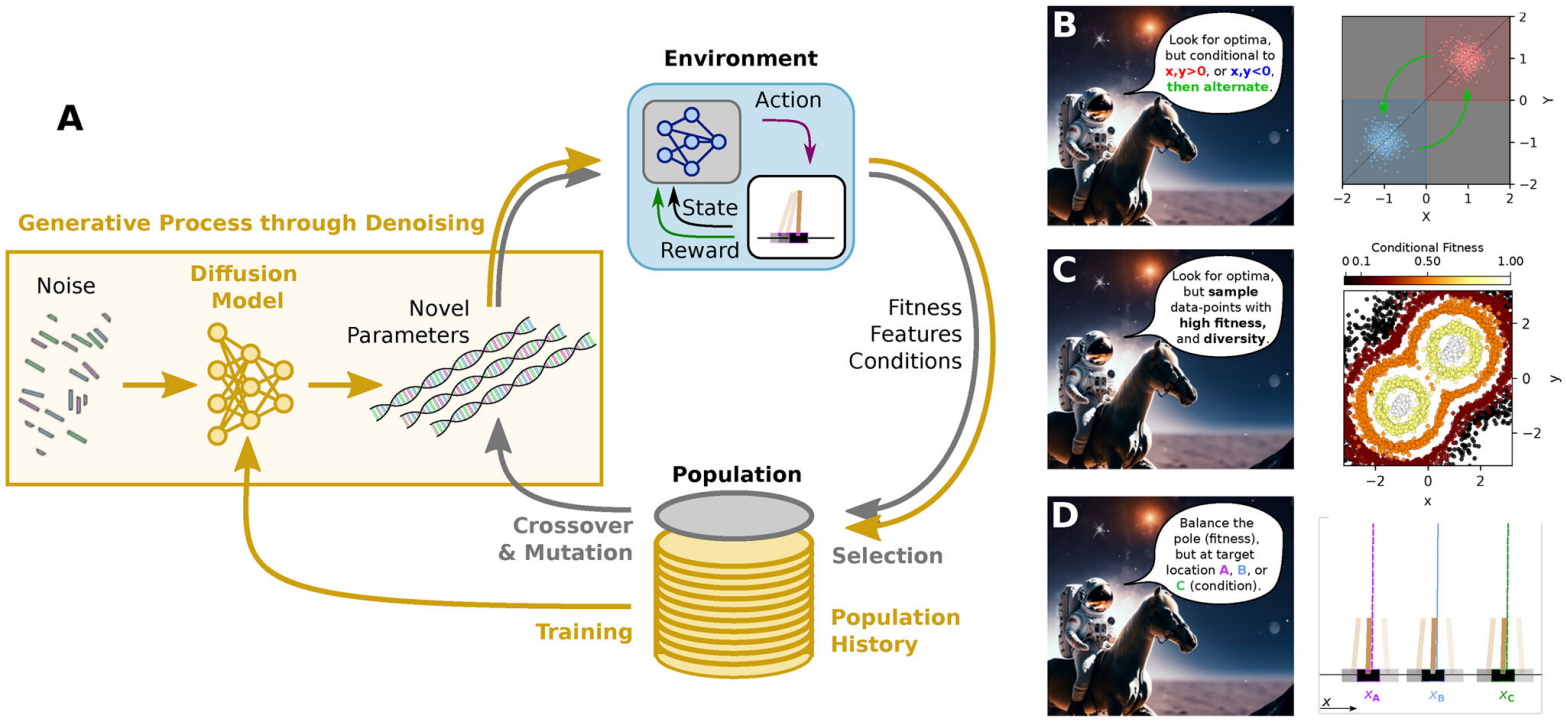
(A) A schematic flow‐chart of a typical evolutionary algorithm (gray arrows and labels) contrasted with our diffusion model (DM)‐based evolutionary optimization (golden arrows and symbols, c.f. Supporting Information Figure S1), showing an evolutionary process either utilizing population‐based (gray) or an ANN‐based DM (golden) as heuristically refined generative model for offspring‐genotype sampling. The DM‐based EA's generative model learns from heuristic experience by training on an epigenetic joint dataset of genome, fitness, and (potentially) conditional feature data of a particular genotype in its environment. We then utilize the successively refined DM to sample high‐quality (high fitness) offspring candidate solutions for a particular environment; via classifier‐free‐guidance techniques [33], this generative process can potentially be biased toward desired target traits in the environment on the phenotypic level. (B) Schematics of DM‐based evolutionary optimization in an environment with two Gaussian optima at $\mu_{\pm }=\left(\pm 1,\pm 1\right)$, but conditioning the search dynamics either to a target parameter range x,y>0 (red) or x,y<0 (blue), or alternate between the two peaks through dynamic conditioning (green). (C) Schematics of utilizing conditional DM evolution of high‐fitness genotypes (low to high fitness color‐coded from black through orange to white) that maintains diversity (spread in parameter space). (D) Schematic behavior of DM‐based conditionally evolved reinforcement learning (RL) [34] agents deployed in the cart‐pole environment [35] the agents are evolved to maximize fitness (balance the pole), but conditionally sampled to steer the cart to a certain location (here A, B, or C) without changing the reward signal.

In this work, we empirically demonstrate that deep learning‐based generative DMs can integrate genotypic reproduction processes from genetic algorithms to adapt within specific environmental and external conditions, guiding heuristic populations toward target parameter directions (see Figure 1). More specifically, we utilize DMs [37, 38], based on Artificial Neural Networks (ANNs), to incorporate generational reproduction in evolutionary optimization (refer to Figure 1A; Figure S1). This approach allows us to explore diverse solutions in complex parameter spaces while achieving significantly higher evolutionary efficiency compared to conventional methods.

Our work redefines evolutionary algorithms as a reproduction mechanism enhanced by DMs. Moreover, by iteratively training the DM on a heuristically acquired dataset buffer after each generation, we incorporate epigenetic [52, 53] associative memory into the evolutionary search process. This associative memory promotes modular adaptations during reproduction, by capitalizing on prior experiences while minimizing inductive biases.

Additionally, through classifier‐free guidance techniques [33], we train generative DMs to conditionally associate high‐quality parameters with specific genotypic, phenotypic, or even population‐wide traits, independently of fitness scores (illustrated in Figure 1B–D, and detailed in the Methods and Supporting Information Section S2). This strategy enables us to control the evolutionary process's search dynamics without altering the objective function but by conditioning the DM to generate offspring with designated target traits or qualities. Consequently, our approach supports biologically inspired multi‐objective optimization without the need for complex reward‐shaping techniques [54]. This is similar to the use of Lagrange multipliers in differentiable optimization, but here serving to impose constraints to effectively guide the search even in rugged, non‐differentiable fitness landscapes.

This work establishes intriguing connections between conditional generative deep learning and evolutionary biology, demonstrating how memory and genotypic conditioning can influence evolutionary optimization algorithms to evolve lineages with specific genotypic, phenotypic, or population‐wide characteristics. Our findings suggest that the denoising process inherent in DMs can serve not only as a powerful tool for evolutionary optimization but also as a bridge between generative AI, DMs, and evolutionary programming—fields that may collectively advance toward more biologically plausible AI frameworks.

In the following, we demonstrate that probabilistic DMs trained “online” on generations of heuristically varied parameter sets can be efficiently utilized as generative models in EAs. We demonstrate our method's competitive performance against other EAs on two toy‐examples before focusing on reinforcement learning tasks. Our approach enables the simultaneous identification of multiple optima, emphasizing diversity and exploration, while still furthering optimal solutions with high efficiency. Furthermore, we demonstrate that classifier‐free guidance techniques can effectively be utilized to conditionally bias the generative process in EAs.

## 3 Results

### 3.1 Diffusion Models Provide a Model‐Free Approach that Adapts Readily to New Problems and Dynamic Environments

Biological evolution is renowned for its adaptability capabilities in (slowly) changing environments [25, 55, 56, 57, 58, 59]. While being increasingly recognized in the computational literature [14], mainstream EAs typically focus on exploring parameter spaces for globally optimal solutions to complex and often rugged, yet generally static objective functions.

To examine the adaptive capabilities of evolutionary processes to changing environmental conditions to mimic a more biologically realistic setting, we introduced a time‐dependent objective function, f(g,τ), where g represents a genotypic parameter realization and τ the current generation, and compare the learning capabilities of our Heuristically Adaptive Diffusion‐Model Evolutionary Strategy (*HADES*) against other different mainstream EAs; see Methods and the Supporting Information Section S3 for algorithmic details on *HADES*. Specifically, we define

(1) $$f\left(g,\tau \right)=\cos\left(\omega \;\tau \right)\;e^{-\frac{{\left(g-\mu_{-}\right)}^{2}}{2\sigma^{2}}}+\cos\left(\omega \;\tau +\phi \right)\;e^{-\frac{{\left(g-\mu_{+}\right)}^{2}}{2\sigma^{2}}}$$

with two Gaussian peaks of STD σ centered at $\mu_{\pm }=\left(\pm 1,\pm 1\right)$ with amplitudes that oscillate phase‐shifted by ϕ across generations τ with angular velocity ω; a static double‐peak problem is recovered by setting ω=0 and ϕ=0.

**FIGURE 2:**
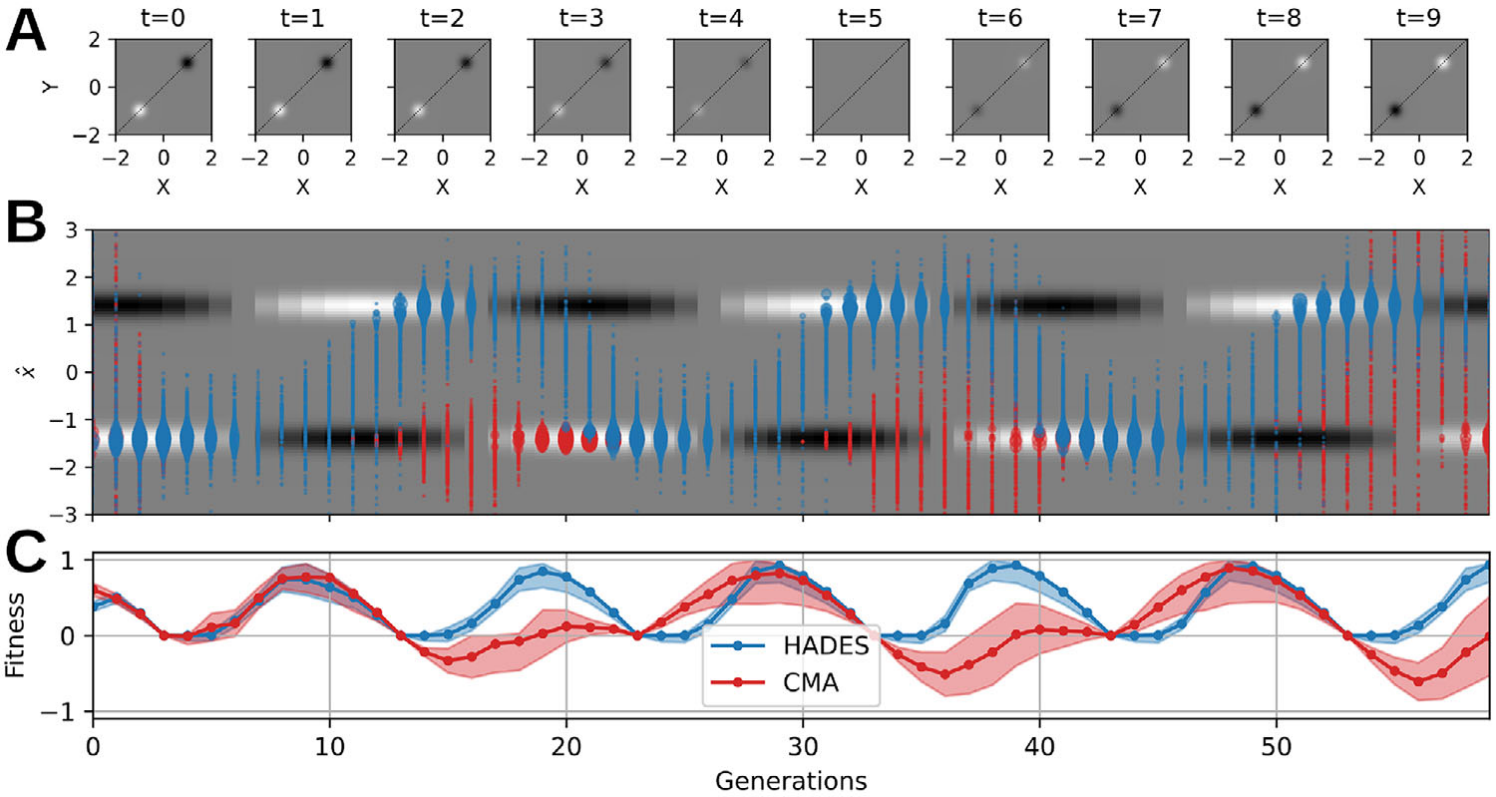
*HADES* adapts to dynamic (oscillatory) environmental changes. (A) Dynamically alternating double‐peak fitness landscape ranging from $f_{\min}=-1$ (black) through $f_{0}=0$ to $f_{\max}=1$ (white) as defined by Equation (1). (B) Population data for *HADES* (blue) and CMA‐ES [22] (red) optimization in the dynamically changing environment illustrated in (A). The 2D data points $g_{i}=\left(x_{i},y_{i}\right)$ are represented as 1D projections ${\hat{g}}_{i}$ onto the diagonal illustrated as dashed lines in (A); the background color indicates the fitness score along x=y, and the radius of the data points ${\hat{g}}_{i}$ scales with fitness $f_{i}$, respectively. (C) Fitness of the data shown in (B): the solid line illustrates the maximum fitness evaluation of the population averaged over ten statistically independent simulations; the shaded area illustrates the average spread of the population's maximum fitness. While *HADES* reliably identifies the current maximum in the alternating double‐peak environment, CMA‐ES clearly struggles to adapt a population to the changing environment in time as the majority of the population resides in the vicinity of one peak.

Thus, we utilize an alternating double‐peak function, where one peak has a positive and the other one negative amplitude by setting ϕ=π, ω=2π/10, and σ=0.1. Over time, the amplitudes periodically alternate in sign, reverting the target of the maximization objective. We apply our *HADES* method and CMA‐ES [22] both with a population size of $N_{p}=256$ and an initial population of STD $\sigma_{I}=0.5$, evaluating each sampled genotypic parameter $g_{i}$ in every generation τ against $f(g_{i},\tau )$; see Supporting Information Section S4 for details. The population dynamics are depicted in Figure 2B. Traditional EAs have very different strategies in updating their generative models across generations due to their inherent inductive biases. Yet, even powerful mainstream approaches such as CMA‐ES may fall short in adapting a population to changing environmental conditions after having seemingly converged on a solutions, even for simple problems as the alternating double‐peak function discussed here. In contrast, *HADES* consistently identifies the periodically changing maximal fitness peak by adapting its population by sampling new offspring through an ever‐refined DM; this is reflected by the respective time‐dependent fitness of both approaches depicted in Figure 2C.

Thus, our *HADES* method offers an efficient and model‐free approach to generate high‐quality genotypic data, particularly excelling in scenarios that require enhanced adaptability capabilities. The intrinsic representative power of DMs enables reliable learning of subtle signals and correlations within arbitrary parameter sets, while their versatile sampling capabilities during the generation phase allow for precise control over the output characteristics. This combination of robust correlation learning and a flexible generative process makes *HADES* particularly well suited to explore complex genotypic landscapes where traditional approaches might struggle to maintain both diversity and quality.

### 3.2 Insights From Developmental Biology: Neutral Multi‐Objective Adaptation via Conditional Diffusion Model Evolution

Typically, EAs aim to find optima in their respective fitness landscapes by maximizing fitness scores through biologically inspired selection and mutation operations. In rugged fitness landscapes, this approach can be exceptionally effective, enabling exploration for global solutions, a distinct advantage over many gradient‐based methods that often get stuck at local optima [21, 60]. However, traditional EAs often struggle with problems that have multiple (unrelated or competing) objectives, as these can create conflicts and frustration in fitness scoring. While mitigation strategies such as problem‐specific reward shaping [54] and curriculum learning techniques [61] exist, these approaches typically demand careful customization and domain expertise. This limitation highlights the need for more robust and adaptable optimization frameworks that can naturally handle multi‐objective scenarios.

We draw inspiration from developmental biology to propose an alternative approach. Recent work [25] suggests that biological evolution does more than just create organisms adapted to specific environments: it produces versatile problem‐solving systems. These biological systems — ranging from gene networks to cells, tissues, organs, organisms, and even organismal collectives, demonstrate remarkable capacity to adapt to environmental cues or domain‐specific challenges in real‐time, while maintaining their overall physiological integrity [9]. Especially during an organism's development, but also during its life time, this adaptation to environmental constraints manifests through sophisticated response mechanisms across multiple scales, while preserving core functionalities without compromising the organism's fundamental fitness. We can interpret this as a form of physiological conditioning of the multi‐scale generative processes of biological systems allowing them to adjust to environmental constraints neutral to their system‐level fitness. The universality of this principle suggests its applicability to mechanisms at the level of RNA and DNA.

DMs offer a particularly suitable framework for incorporating external (environmental) cues in their generative process through classifier‐free guidance [33]: At their most basic level, DMs are trained to generate, i.e., sample novel data ${\hat{x}}_{0}$ from a probability distribution p(x) that conforms to a training dataset $X=\{g_{1},\text{…}\;,g_{N}\}$. The true power of DMs, however, lies in their ability to conditionally sample novel data points ${\hat{x}}_{0}^{\left(c\right)}$ that exhibit desired target traits or features c. These feature vectors c numerically classify selected qualities of the data, which are here formally encoded via a custom, not necessarily differentiable mapping c=c(g). Thus, through classifier‐free guidance, we can explicitly steer the generative process of DMs to sample biased data points ${\hat{x}}_{0}^{\left(c\right)}$ from a conditional probability distribution ${\hat{x}}_{0}^{\left(c\right)}∼p\left(x|c\right)$, where the generated output exhibits specific desired target traits such that $\hat{c}=c\left({\hat{x}}_{0}^{\left(c\right)}\right)\approx c$; see also Methods and Supporting Information Section S2.

This approach forms the foundation of modern text‐guided image and video generation systems [40, 41]. During training, the DM learns to associate [50] data g with corresponding numerical conditions c=c(g). When deployed, it can generate data conforming to custom target conditions, effectively translating abstract constraints into concrete output characteristics.

**FIGURE 3:**
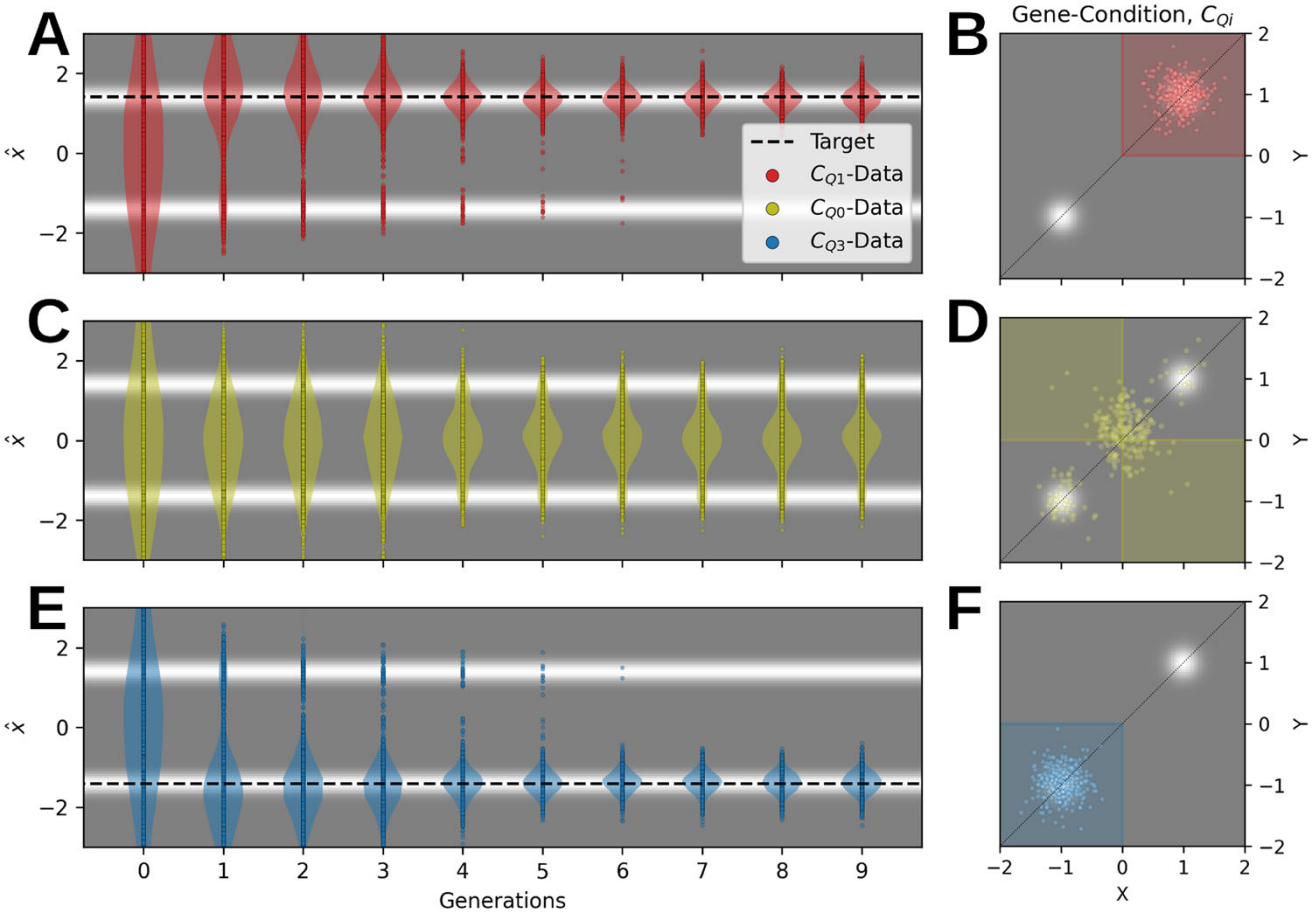
Conditional evolutionary optimization to explore selected target parameter regions in 2D double‐peak fitness landscape. (A, C, E) Fitness landscape (grayscale) and distribution of population data (projected onto the x=y line) for ten statistically independent simulations vs. generations as violin plot, conditioning the DM to sample novel data points from the first quadrant x,y>0 (red), second and forth quadrants x×y<0 (yellow), and third quadrant x,y<0 (blue); datapoints are projected onto the x=y diagonal. (B, D, F) Fitness landscape in gray‐scale from $f_{\min}=0$ (gray) to $f_{\max}=1$ (white) with overlaid data points (colored dots) of an exemplary population from panels (A, C, E), respectively, after nine generations; the target quadrants are illustrated as color‐shaded areas.

Here, we employ classifier‐free guidance to constrain the genotype sampling process in our *HADES* method, ensuring that resulting phenotypes meet specific target conditions $c^{\left(T\right)}$ in their environments. Crucially, this process operates independently of fitness scores! We call this approach Conditional, Heuristically‐Adaptive ReguLarized Evolutionary Strategy through Diffusion (*CHARLES‐D*), which, in contrast to *HADES*, modifies the denoising process by incorporating target conditions into the error estimate: $\varepsilon_{\theta }\left(x_{t},t,c^{\left(T\right)}\right)$. The implementation involves three key steps: First, we associate each element $g_{i}$ in the heuristic training data X with a numerical feature vector $c_{i}=c\left(g_{i}\right)$ that quantifies specific traits. These traits can encompass genotypic parameter qualities (see Figures 3 and 5), fitness‐related and population‐level characteristics (see Figure 6), or phenotypic traits (see Figure 7). Second, at each generation, we train the DM on paired data $\left(g_{i},c_{i}\right)$; the DM's loss function evaluates the model's ability to denoise data $g_{i}$ at fixated corresponding features $c_{i}$ that are unaffected by noise. Third, during the generative phase of a new population we employ custom target conditions $c^{\left(T\right)}$ to guide evolutionary trajectories through the parameter space. This leads to conditionally sampled individuals that increasingly exhibit the desired condition‐specific traits while improving their fitness across generations.

**FIGURE 4:**
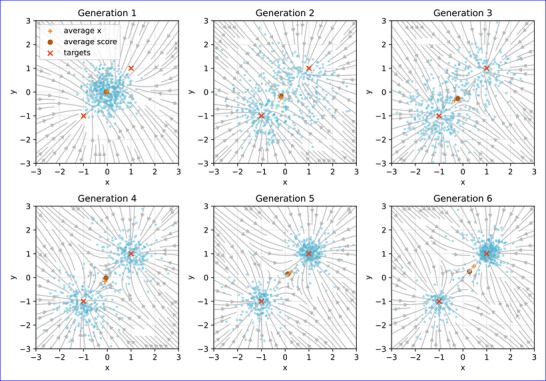
Visualization of the score function $\nabla_{g}\log p\left(g\right)$ (gray streamlines) and the population (blue dots) across different generations. Red crosses indicate the optimal points. In early generations, the score function directs the population toward the center of the landscape. Over time, it becomes more detailed and splits, guiding the population to converge on two separate solutions. The average genome $\bar{g}$ is plotted as an orange cross, with its average score shown as a brown vector. A key observation is that the average score remains small even as the score function pulls the population toward distinct high‐density regions. This highlights the importance of maintaining multi‐modal solutions for escaping local minima and saddle points.

**FIGURE 5:**
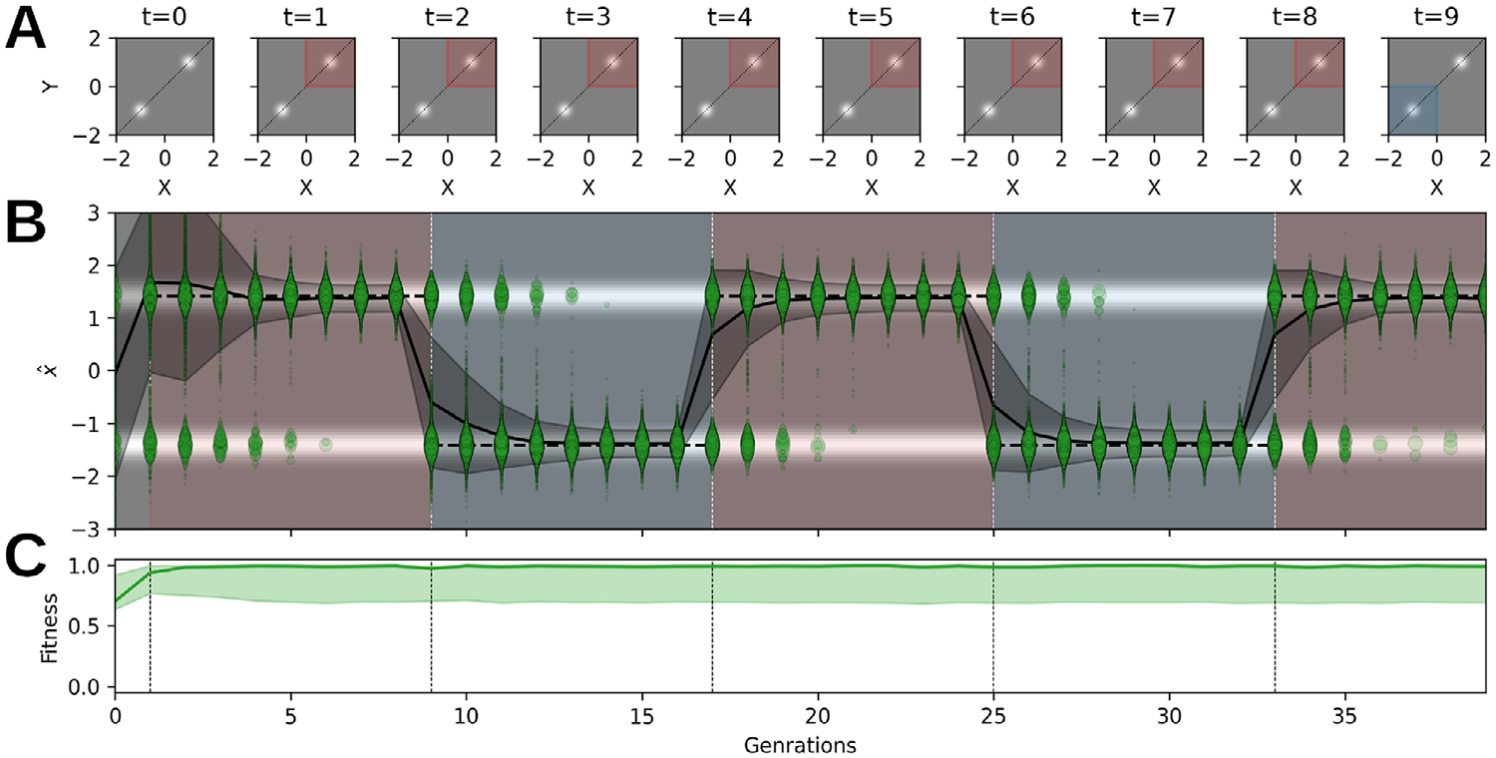
Dynamically Conditioning Genetic Parameters. (A) A static double‐peak fitness landscape, ranging from $f_{\min}=0$ (gray) to $f_{\max}=1$ (white) as defined by Equation (1) with ω=ϕ=0. Dynamical conditioning allows exploration of the first quadrant (red) or the third quadrant (blue), see also Figure 2. (B) The fitness landscape (grayscale) and the distribution of population data (projected onto the x=y line) for ten statistically independent simulations versus generations (radii of green‐colored data points scale with fitness). The average population mean is illustrated by the black solid line, while the gray area marks the STD. The oscillating red ↔ blue color‐coding of the fitness landscape reflects the applied condition for the first or third quadrant during DM sampling, respectively leading to jumps of the population from one peak to the other; transition generations are marked by white vertical dashed lines. (C) The mean and STD of maximum fitness (solid green line and shaded area) demonstrate consistently high fitness values, even during transitions of the conditional sampling.

**FIGURE 6:**
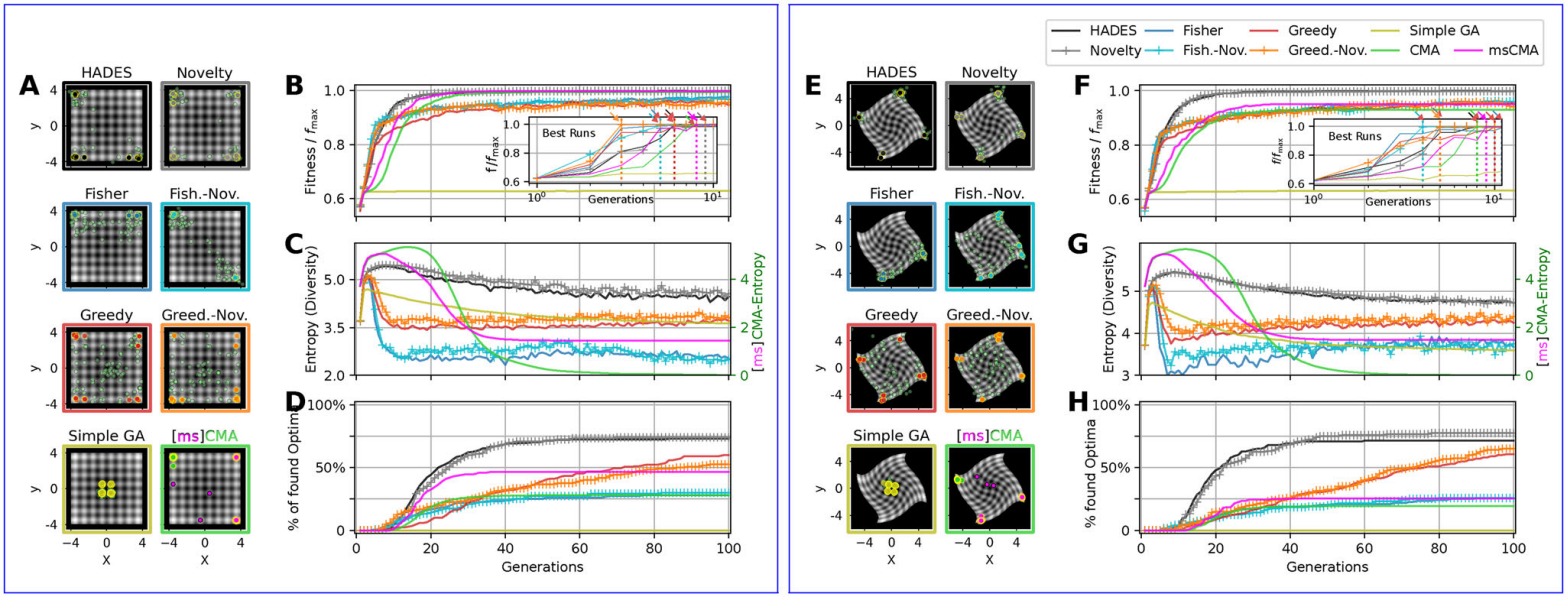
Fitness‐ and Novelty‐Conditional Benchmarks for the Rastrigin (A–D) and “Twisted”‐Rastrigin (E‐H) Task (see Supporting Information Section S6.3 for details). (A, E) The fitness landscape of the Rastrigin (A) and “twisted”‐Rastrigin task (E) with overlaid exemplary results (elite solutions in thick colored‐coded circles, and final population in small green circles) after 100 generations of optimization with different *HADES* and *CHARLES‐D* solvers (see titles and colored‐coded borders) contrasted with SimpleGA (olive), CMA‐ES (limegreen), and multi‐start CMA‐ES (msCMA; magenta) algorithms (bottom row, see text); the fitness landscape is indicated in gray scale ranging from $f_{\min}=0$ (black) to $f_{\max}=64.62$ (white). (B,F) The maximum fitness from different solver configurations (*c.f*., color‐coding and panels (A) and (E), respectively), (C, G) the entropy‐based diversity of the population (see Supporting Information Section S6.4), and (D,H) the number of cumulatively identified solutions across successive generations for the Rastrigin (B‐D) and twisted‐Rastrigin (F‐H) tasks, averaged over 50 statistically independent simulations, respectively. The instets in (A, E) show the cumulatively best fitness versus generations from all statistically independent simulations for the different solvers (CMA‐ES and msCMA results are displayed in respective limegreen and magenta color‐coding in the same panel), and the colored arrows indicate the generation when the problem was solved in the least number of generations by a particular solver for both tasks. Both the vanilla *HADES* and the novelty‐conditional *CHARLES‐D* methods reliably identify multiple optima at the corners of the fitness landscape (D, E): on average, ≈75% of the target peaks are identified, while in ≈10% of all simulations 100% of the solutions are found successfully. The diversity measures shown in (C, G) and (D, H) indicate that the novelty‐conditional *CHARLES‐D* method maintains an increasingly diverse populations of high‐quality genetic material compared to the respective unconditional cases.

**FIGURE 7:**
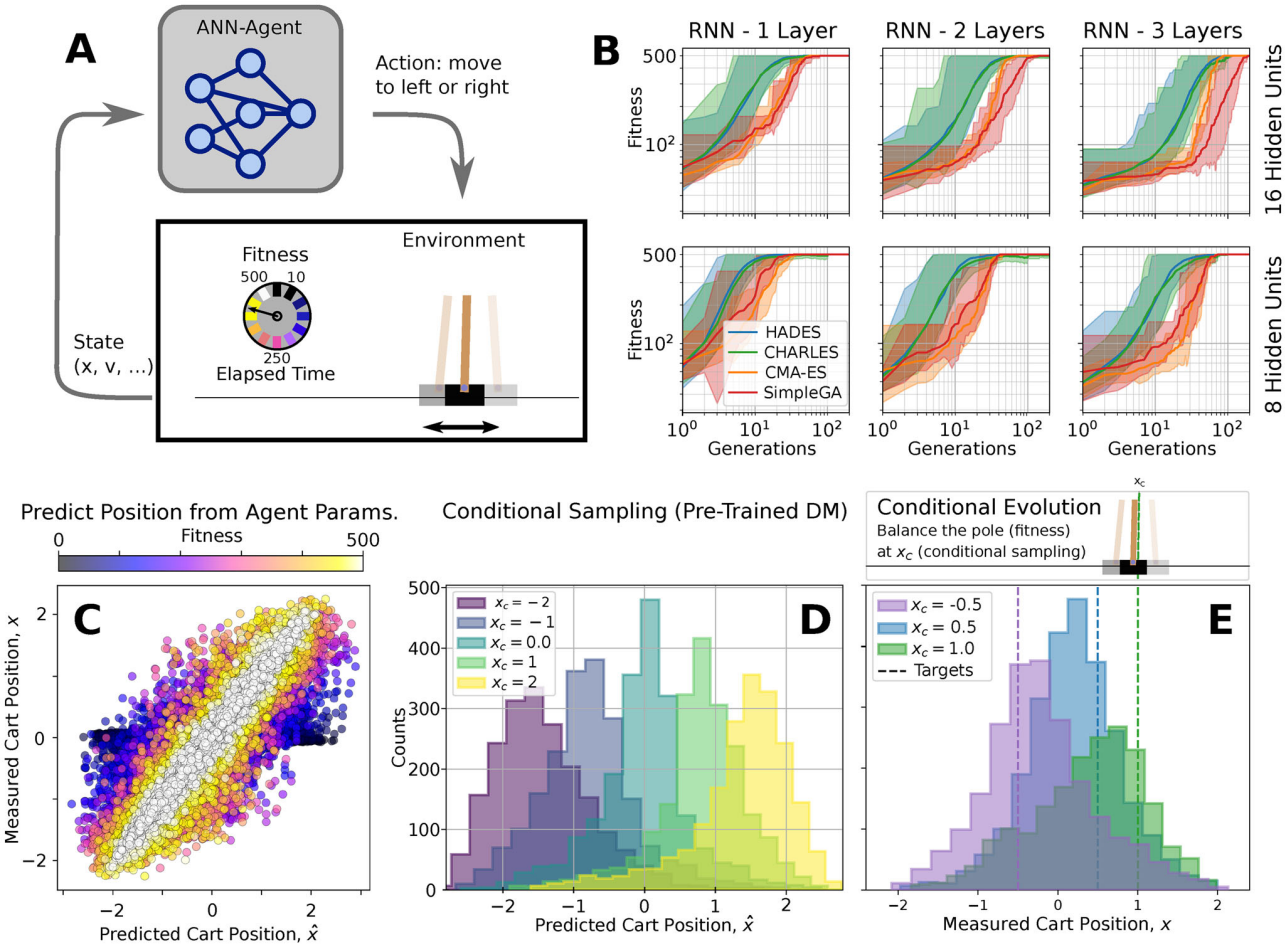
(A) Sketch of an ANN‐based RL‐agent controlling the cart in a cart‐pole environment. (B) Training performance of *HADES*, Fisher‐conditional *CHARLES‐D*, CMA‐ES, and SimpleGA on the cart‐pole task with different ANN architectures. The solid lines emphasize the mean maximum fitness of the different solvers (color‐coding) which we averaged over statistically independent evolutionary optimization runs. The shaded areas larger (smaller) than the mean at a given generation indicate the historically best performing solution (STD) across all independent simulations. (C) Accuracy of predicting a cart‐pole agent's resting position from its ANN parameters (by training a four layer ANN with of 48 hidden units and LeakyReLU activation on a dataset of ten unbiased evolutionary runs using the *HADES* method) with good accuracy for high‐fitness individuals (color‐coded). (D) Estimated resting position of cart‐pole agents sampled conditionally for target resting positions $x_{C}=\left\{-2,-1,0,1,2\right\}$ (color‐coding) by a pretrained DM (two layers with 324 hidden units and ELU activation for 2000 episodes on the same data as in (C)). (D) Resting positions of conditionally evolved lineages with the *CHARLES‐D* method that give rise to RL‐agent policies with desired behavior of target resting positions $x_{C}=\left\{-0.5,0.5,1.0\right\}$ (color coded); the DMs in (E) are not pretrained as in (D), but trained on heuristic data of the respective evolutionary lineage. All solvers maintain a population of $N_{p}=256$ candidate solutions across generations to ensure a fixed fitness evaluation budget at a given generation.

In our first example, we apply the *CHARLES‐D* method to find optimal solutions to the static double‐peak objective function given by Equation (1) with ω=ϕ=0, while conditioning the generative process of the DM to predominantly sample genotypic offspring in specific quadrants of the 2D plane. The results are illustrated in Figure 3. Starting from a normal distributed population with $\sigma_{I}=2$, we associate individual genotypic parameters $g_{i}=\left(x_{i},y_{i}\right)$ with their corresponding quadrant in the parameter space: For data points in the first quadrant $\left(x_{i},y_{i}>0\right)$, we assign the numerical classifier value $c_{i}^{\left(Q1\right)}=1$, for data points in the second and fourth quadrant $\left(x_{j}\times y_{j}<0\right)$, we assign $c_{j}^{\left(Q0\right)}=0$, and for data points in the third quadrant $\left(x_{k},y_{k}<0\right)$, we assign $c_{k}^{\left(Q3\right)}=-1$. The process then proceeds through three sequential steps: First, we evaluate the fitness and feature vectors for all individuals of a given generation. Second, the DM undergoes joint training on the set of associated data and quadrant‐classifiers $\left(g_{i},c_{i}^{\left(Qj\right)}\right)$. Finally, when sampling the genotypes of the next generation, we select a particular target quadrant, T, to condition the generative phase of the DM by $c^{\left(QT\right)}$; see Supporting Information Section S6.

In Figure 3A,C,E, we present population data across consecutive generations in this double‐peak environment for ten statistically independent evolutionary lineages. We condition the *CHARLES‐D* method to sample from one of three distinct regions: the first quadrant, T=1, the second and fourth quadrants, T=0, or the third quadrant, T=3. Snapshots of converged generations for each case are superimposed on the fitness landscape in Figure 3B,D,F. The results demonstrate the remarkable effectiveness of conditioning on the first and third quadrants: despite both fitness peaks being qualitatively equivalent, the *CHARLES‐D* method consistently converges to the peak located in the conditionally targeted quadrant. Conversely, conditioning DM‐sampling on $c^{\left(Q0\right)}$ results in frustration effects, as the second and fourth quadrants lack fitness peaks. We conclude that conditioning in *HADES* serves as a powerful regularizer for genotypic exploration with DM evolution. This mechanism enables selectively biasing the evolutionary process to either explore or avoid specific regions of the parameter space, or more broadly, solutions with particular genotypic qualities, without altering the problem's fitness score. This neutral adaptation toward desired qualities is achieved elegantly through DM conditioning with classifier‐free guidance techniques. Furthermore, these conditions can be formulated flexibly and orthogonally to the optimization problem's fitness‐score, without requiring differentiability. Consequently, *CHARLES‐D* provides an elegant approach to multi‐objective optimization that eliminates the need for cumbersome reward shaping techniques [54].

### 3.3 The Score Function Evolves Into a Gradient of the Fitness Landscape

To elucidate the evolutionary dynamics and highlight the advantages of our approach, we analyzed the evolution of the score function across generations. In score‐based generative modeling, the score function is defined as the gradient of the log‐probability of the data distribution, $\nabla_{g}\log p\left(g\right)$. Within our framework, the diffusion model is trained on heuristically acquired high‐fitness genotypes, implicitly learning a distribution p(g) that reflects the fitness landscape. Consequently, the score function acts as a vector field guiding the search toward regions of higher fitness. This gradient can be estimated directly from the trained noise‐prediction network $\varepsilon_{\theta }$ without requiring the fitness function itself to be differentiable [38]:

(2) $$\nabla_{g}\log p\left(g_{t}\right)\approx -\frac{\varepsilon_{\theta }\left(g_{t},t\right)}{\sqrt{1-{\bar{\alpha }}_{t}}}$$

During the evolutionary process, our method iteratively refines this score function. As illustrated in Figure 4, the score function initially points toward a general high‐fitness region before resolving into distinct vectors that guide the population toward multiple optima simultaneously. This demonstrates how the underlying generative model of the fitness landscape is progressively optimized “online”. For score function evolution with conditions, please see Supporting Information Figures S3,S4, and S5.

This score‐based perspective reveals a fundamental advantage over methods like CMA‐ES, which often model the search distribution with a single, unimodal function (e.g., a multivariate Gaussian). In complex or symmetric fitness landscapes, such as those with multiple peaks or saddle points, the mean of a unimodal search distribution can fall into a low‐gradient area, causing the “average” gradient to vanish and stalling the optimization process. In contrast, our diffusion‐based approach is not susceptible to this issue. This is also validated in experiments shown in Figure 6, where the multi‐start CMA‐ES often struggled in the center of the landscape, suggesting average gradient vanished at symmetric local loss landscapes. The score function is position‐dependent, enabling it to capture complex shape of the fitness landscape. This shows that maintaining population diversity and pursuing multiple solutions is not an independent, secondary objective but rather an intrinsic feature critical for effective optimization in complex solution spaces, which are common in fields such as physics, chemistry, and engineering.

### 3.4 Learning From Past Experience: DM‐Based Generative Samplers Provide a More Powerful and Biologically Inspired Framework for Modular Evolutionary Processes

Biological evolution exhibits an inherent capacity to utilize existing, heuristically acquired knowledge for species adaptation in changing environments [11, 12, 13, 14, 62, 63]. This capability stems from the recombination of established genetic material across evolutionary time‐scales. Thus, the exploitation of previously explored solutions and their adaptation to novel contexts represents a fundamental principle of biological evolution [55, 56, 57, 58, 59]. Within traditional EAs, the integration of past experience remains largely limited to heuristic information stored in the current population, or within the generative model employed by techniques such as CMA‐ES for offspring sampling. Consequently, the generative process in current EAs maintains limited memory of previously explored solutions, leading to inefficiencies from either memory loss or the intrinsic inductive bias of generative models. Generative DMs, however, can be equipped with epigenetic memory [52, 53] by preserving information about previously explored solutions. This capability can be implemented through dataset buffering or DM retraining across multiple generations. These approaches offer a model‐free methodology to enhance evolutionary sampling capabilities based on past experience, while simultaneously testing new environmental hypotheses through an iteratively updated generative process. Information holds significant value in optimization, particularly in computationally expensive tasks, where maintaining reliable memory is crucial. Refining a model‐free generative process via heuristically acquired dataset buffers therefore presents an efficient approach to leverage prior knowledge in evolution. This significantly improves the capacity of an evolutionary process (as modeled by *HADES* and *CHARLES‐D*) to navigate complex parameter spaces more effectively. Moreover, this ability to learn from past experience substantially augments the adaptability and transferability of evolutionary algorithms to changing environments.

To demonstrate this, we conduct the following experiment: (i) utilize the static double‐peak environment (see Equation 1), (ii) equip *CHARLES‐D* with a memory buffer dataset spanning the past five generations, where only the lowest fitness solutions are replaced with current population data, and (iii) implement time‐dependent conditioning that alternates between the first and third quadrants in the parameter space. We again start with an initial normal distributed population with $\sigma_{I}=1$. We then conditionally sample solutions from the first quadrant for eight consecutive generations using $c^{\left(QT\right)}=c^{\left(Q1\right)}$ for τ∈[1,8] during the DM's generative process, followed by conditioning to the third quadrant for the next eight consecutive generations using $c^{\left(QT\right)}=c^{\left(Q3\right)}$ during τ∈[9,16], as shown in Figure 5A. This process repeats periodically for each lineage, with DM initialization and training on the memory buffer dataset after each generation. The experiment is repeated ten times, with population dynamics and elite fitness‐scores presented in Figure 5B,C. The results reveal that *CHARLES‐D* consistently converges to the specified target peak, maintaining performance even under dynamically modified conditions during evolution. Notably, the epigenetic memory enables the evolutionary process to perform discontinuous but targeted transitions between previously visited high‐fitness regions when conditions change between $c^{\left(Q1\right)}↔c^{\left(Q3\right)}$. Rather than exhibiting slow continuous adaptation between peaks located at ±μ, we observe instantaneous repopulation of the new target peak and heuristic fade‐out of the previous one. This rapid, targeted readaptation maintains consistently high fitness scores across conditioning transitions (see Figure 5C), contrasting with the oscillatory elite fitness scores observed in the dynamically changing environment discussed in Figure 2C. This behavior bears striking resemblance to the ecological memory described in [14], though achieved here through a heuristically adaptive DM.

In conclusion, our experiments demonstrate the powerful capability of generative DMs to leverage evolutionary history for enhanced optimization processes. The integration of memory components and conditioning schemes in *CHARLES‐D* and *HADES* significantly improves the adaptive capabilities of evolutionary algorithms in dynamic environments. Our findings extend beyond algorithmic improvements: they raise fundamental questions about unconventional memory mechanisms in biological systems. The observed parallels between DM‐based evolutionary processes and biological adaptation mechanisms suggest promising new directions for understanding the principles governing both artificial and natural evolutionary systems.

### 3.5 Improving on Past Experience: Conditioning for Higher Fitness Can Improve Learning Performance, but Increases Greediness

Next, we investigate the application of fitness conditioning during the DM's generative process: We can jointly train the DM on associated parameters and fitness scores $\left\{g_{i},f_{i}=f\left(g_{i}\right)\right\}$, conditioning the sampling process to generate offspring that potentially achieve a higher target fitness than any previously observed [45], $f^{\left(T\right)}>\max_{i}\left(f_{i}\right)$.

Given that the maximum fitness or reward can not be known a priori, we propose sampling the target fitness for conditionally generating the next generation based on Fisher's fundamental theorem of natural selection, which states that [64] “The rate of increase in fitness of any organism at any time is equal to its genetic variance in fitness at that time”. This principle on the rate of expected fitness improvement has intriguing links to inverse reinforcement learning (IRL) [65, 66], as it can not be assumed that conditioning the DM on arbitrarily large fitness (i.e., significantly surpassing the training data) will yield reasonable offspring parameters, especially in early stages of the evolutionary search process.

However, to avoid this algorithm to become too greedy, we here introduce two flavors of fitness sampling, (i) Fisher‐conditioning $c^{\left(TF\right)}∼\left|N\left(\mu =f_{\mu },\sigma =\sigma_{f}\right)\right|$, and (ii) Greedy‐conditioning of target fitness $c^{\left(TG\right)}∼N\left(\mu =f_{\max},\sigma =\sigma_{f}\right)$, where $f_{\mu }$ is the mean, $f_{\max}$ the maximum, and $\sigma_{f}$ the STD of the fitness scores $f_{i}$ of the current population.

Illustrative examples of sampling parameters conditional to target fitness values by a DM pre‐trained [45] on the double‐peak task are shown in Figure 1C and Figure S2. In Figure 6A–D, we present optimization results for different configurations of *HADES* and fitness‐conditional *CHARLES‐D* applied to the inverted and truncated Rastrigin task, a periodically oscillating function in the 2D plane with four optima located at |x|=|y|≈3.5 (see Supporting Information Section S6.3 for details). For simplicity, we henceforth refer to the inverted and truncated Rastrigin task simply as Rastrigin task.

Our findings indicate that this fitness conditioning can indeed significantly improve the learning capabilities of *CHARLES‐D*, which opens new avenues for exploring complex parameter spaces. However, aiming for too large fitness increases across generations might also lead to more greedy behavior of our algorithm and thus suboptimal convergence, which we will delve into detail in the following section.

### 3.6 Novelty‐Conditional Sampling: A Good Regularizer for Exploration and Maintaining Diversity

Typically, EAs aim at identifying solutions with optimal fitness values in rugged parameter landscapes. However, depending on the fitness landscape, this can be highly non‐trivial and requires a dedicated balance between exploration and exploitation, see for example [67]. Recent discussions, originating from developmental biology [9, 25] suggest that biological systems operate under a different paradigm, where agents continuously explore novel situations to maintain their integrity and adapt to changing environments. This inherent drive for novelty leads to the creation of novel challenges, requiring further adaptation, and thus creating a diversification scaffold [68].

It has recently been argued [68], that the fundamental drive for novelty in biological systems could be rooted in the phenomenon of boredom: biological agents on a variety of scales will seek novel stimuli if their sensory inputs stagnate too long. This is reflected in their intrinsic drive for exploration and discovery, but not necessarily aligns with traditional optimization objectives or search strategies. In fact, incorporating novelty [69] or a bias toward quality‐diversity [70] into optimization processes such as EAs significantly improve their performance. These techniques reinforce search directions in novel parameter regions while penalizing (over)exploitation of already experienced solutions. Instead of directly optimizing for novel and diverse solutions via modified fitness scores, we propose to use novelty‐conditional sampling via *HADES*.

We utilize a metric for diversity in the heuristic dataset buffer of our *CHARLES‐D* method, and condition the DMs generative process to sample diverse genotypes. Specifically, we define diversity $\delta_{i}$ of a single data point $g_{i}$ similar to the non‐parametric k‐nearest‐neighbor entropy estimator [71] as $\delta_{i}=\log\left(\frac{1}{N-k}\sum_{j=k}^{N}d_{ij}\right)$, i.e., as the logarithm of its mean distance in parameter space to all other data points $g_{j}$ with a distance $d_{ij}=\left|g_{i}-g_{j}\right|$ larger than the k‐th nearest neighbor distance $d_{\mathrm{knn}}$ in the dataset buffer. In practice, we use this diversity metric as a novelty condition $c_{i}^{\left(N\right)}=\delta_{i}$ when conditionally training *CHARLES‐D* during the evolutionary process, and sample novel data points with target conditions $c^{\left(NT\right)}$ that maximize diversity by favoring large $\delta_{i}$ (see Supporting Information Section S5 for details).

We demonstrate this approach on the Rastrigin task (see Supporting Information Section S6.3) and show the results in Figure 6A–D, while a minimal example of the static double‐peak environment can be found in Figure S2. We compare fitness and diversity measures of different parametrizations of *HADES* and *CHARLES‐D* instances, along with mainstream methods such as a simple genetic algorithm [72] (SimpleGA), CMA‐ES [22], and a multi‐start CMA‐ES variant. Specifically, we utilize the *HADES* method as baseline (fitness optimization without diversity condition), and use different combinations of Novelty‐based conditioning and Fisher‐ and greedy fitness‐conditioning in different *CHARLES‐D* instances, where we use single or multiple conditions during evolutionary optimization (snapshots of converged populations are presented in Figure 6A). All solvers maintain a population of $N_{p}=256$ candidate solutions across generations to ensure a fixed fitness evaluation budget at a given generation.

In our simulations, we start with a narrowly distributed initial condition with $\sigma_{I}=0.2$, challenging the respective EAs to explore from a central valley of the fitness landscape and find the different peaks located at |x|=|y|≈3.5 through exploration. We emphasize that the solutions are not contained in the initial population (and thus are not part of the initial dataset buffer). We refrain from using elitism in any solver, and do not utilize explicit crossover operations in *HADES* and *CHARLES‐D* optimizations. Offspring – at every generation – are directly sampled from the respective solvers' generative models: the population‐based crossover and mutation dynamics for the SimpleGA; the refined Gaussian for CMA‐ES; $N_{\mathrm{ms}}$ independent Gaussian distributions for multi‐start CMA‐ES (of correspondingly reduced population sizes $N_{p}^{*}$, to match the fixed fitness evaluation budget for a total population size of $N_{p}=N_{p}^{*}\times N_{\mathrm{ms}}=256$); and the constantly refined DMs for *HADES* and *CHARLES‐D* (for more simulation details see Supporting Information Section S4).

We observe from Figure 6B that *HADES* and the Novelty‐conditional *CHARLES‐D* method excel at this task: both methods quickly and reliably identify optimal solutions in 50 statistically independent optimization runs. These approaches demonstrate faster convergence than CMA‐ES and multi‐start CMA‐ES, while the SimpleGA fails to identify the global optima and resides at the nearest locally‐optimal peaks to the center. Moreover, our methods intrinsically maintain a high level of diversity evidenced in the constantly high entropy of the parameters in the population (see Figure 6C). This diversity manifests through the fact that our methods can identify multiple optima in complex fitness landscapes reliably. We quantify this in Figure 6D where we present the average number of solutions found cumulatively during a single lineage for all investigated algorithms. We consider a peak in the Rastrigin problem as “found” during the course of a single evolutionary search, if at least 10 independent parameter samples of a single lineage are located within a radius of 0.25 around this particular peak. Cumulatively found solutions during a particular lineage can thus increase with generations if different peaks are either explored simultaneously or successively by the search procedure. The results show that the vanilla *HADES* and the Novelty‐conditional *CHARLES‐D* can identify ≈75% of all target peaks reliably during a single lineage, and identify all four peaks of the Rastrigin task successfully in ≈10% of all simulations.

The greedy fitness‐conditioning solves the problem more quickly (c.f. inset in Figure 6B) but demonstrates slower convergence on average compared to the previous two solver configurations. The greedy exploration maintains high entropy and, unexpectedly, exhibits explorative behavior over time: as illustrated in Figure 6D, the greedy *CHARLES‐D* configuration, on average, initially identifies a single peak, yet despite the convergence of average fitness, the number of discovered solutions steadily increases with successive generations.

Fisher fitness‐conditioning and CMA‐ES demonstrate limited diversity and typically converge to a single optimum. Nevertheless, CMA‐ES consistently identifies the global optimum in the Rastrigin problem, where the parameter space aligns well with the method's Gaussian generative model [22]: The algorithm explores the environment by adapting the covariance matrix of a multivariate Gaussian distribution (matching the search space dimension) to best fit the likelihood of the data. The EA's reproduction step is realized by sampling novel data points from this refined generative model, exploring the parameter space through successive Gaussian model adjustments.

For the multi‐start CMA‐ES, we exemplarily utilize $N_{\mathrm{ms}}=5$ independent CMA‐ES instances with corresponding Gaussian distributions, which optimize the Rastrigin problem on independent sub‐populations of sizes $N_{p}^{*}=\left\{52,51,51,51,51\right\}$ – to match the fixed fitness evaluation budget per generation for a total population size of $N_{p}=256$. The different sub‐populations typically converge to different locations in the parameter space, leading to an increased diversity score (see Figure 6C). However, the reduced sub‐population sizes limit the explorative power of the individual CMA‐ES instances, which then often converge to local rather than global optima (c.f., bottom right panel of Figure 6A), while still allowing the algorithm to reliably identify ≈50% of all target peaks (see Figure 6D).

In our Rastrigin example, the directions of the nearest local optima near the centered initial generation align with the global optima. Through expansion of the covariance matrix in a particular principal direction, i.e. (x=±y), CMA‐ES efficiently identifies a global optima very efficiently in few generations. However, this search strategy exhibits a strong inductive bias and can be inefficient when the search space requires complex reorientations of search directions. We propose that deep‐learning‐based DMs provide a more flexible approach to understanding the parameter space due to the universal approximation theorem [73], reflected in their adaptable search strategy.

To illustrate this, we introduce a “twisted” variant of the Rastrigin problem. By transforming the coordinate space $g\to \tilde{g}$ non‐linearly, we warp the peaks of the Rastrigin function along an outward spiraling pattern, where global maxima are twisted relative to the initial local optima near the center (see Figure 6E and Supporting Information Section S6.3 for details). This geometric modification of the fitness landscape significantly impacts both the CMA‐ES' and multi‐start CMA‐ES' reliability, while our *HADES* and *CHARLES‐D* methods maintain their performance despite increased complexity (see Figure 6E–H and also Supporting Information Section S6.5).

In all situations presented in Figure 6, using the Novelty‐condition introduces a repulsive bias between parameter clusters during the DM's generative process. As illustrated in Figure 6C,G, D,H, this leads to two key outcomes: accelerated exploration of high‐fitness regions in the parameter space and, simultaneously, to increased population diversity. While CMA‐ES, SimpleGA, and Fisher‐based *CHARLES‐D* converge to a single solution with limited diversity, multi‐start CMA‐ES, *HADES*, and particularly the Novelty‐conditional (greedy) *CHARLES‐D* solvers maintain significantly higher diversity even after fitness convergence. Multi‐start CMA‐ES achieves this by maintaining multiple independent populations, integrating little information across lineages. In turn, *HADES* and *CHARLES‐D* demonstrate that a single population with a more flexible generative model is capable of exploring multiple solutions at once, and that the population intrinsically explores new parameter space regions if environmental conditions are changing. Notably, we utilize a static fitness landscape in Figure 6, but we might consider the population as part of the environment, competing for resources. Novelty conditioning is sensitive to adaptations, especially to clustering of the population in an environment, rendering the environment of *CHARLES‐D* as dynamically changing. This, in turn, impacts the reproductive process of the DM across generations, promoting explorative behavior. On average, the Novelty‐conditional *CHARLES‐D* method shows increased diversity compared to corresponding non‐novelty‐conditional configurations.

Conditioning on novelty effectively applies neutral selection pressure, promoting population diversity while independently optimizing fitness scores. This approach serves as an effective regularization mechanism for DM‐evolution, generating diverse and novel solutions while maintaining the ability to exploit clusters of elite solutions.

### 3.7 Genetically Conditioning Behavior: How Information Traverses Scales

So far, we have demonstrated how the *CHARLES‐D* method can be applied to constrain (i) the search dynamics in the parameter‐space, (ii) the fitness quality of the samples across generations, and even (iii) improve population wide diversity in a diffusion evolution optimization process. Building upon these results, we explore its application in selectively sample genotypic parameters to achieve desired phenotypic traits. Specifically, we explain whether we can conditionally train the DM in *CHARLES‐D* using both (i) genotypic representations and (ii) associated phenotypic qualities of agents in Reinforcement Learning (RL) environments [34]. The goal is to selectively sample RL agents during an evolutionary process that exhibit specific target behaviors, notably without pretraining the DM.

Traditional RL applications aim to identify policies that enable autonomous agents to effectively navigate their environments: RL agents perceive different aspects of their environment, such as state information and a reward signal, and need to propose actions that maximize reward acquisition. The policy of an agent, i.e., its internal decision‐making machinery, is often modeled by Artificial Neural Networks (ANNs) receiving environmental states as input, and outputting high‐quality actions that enable the agent to navigate its environment efficiently. The challenge thus is to identify ANN parameters that enable agents to maximize reward acquisition corresponding to high fitness scores. This is often achieved by gradient‐based RL algorithms [74], which require careful curation of differentiable reward signals. Especially in multi‐objective scenarios either cumbersome reward‐shaping [54] or curriculum learning techniques [61] are necessary to balance different reward signals, or the environment needs to be extremely general [75] leading to substantial computational overhead and potentially unpredictable behavior [76]. In contrast, EAs have proven highly successful to evolve slim and problem‐specific ANN‐based RL agent policies simply based on cumulative reward measures and often result in much more robust, transferable, and interpretable agent policies [31, 72, 77, 78, 79, 80, 81]. As demonstrated in Figure 7, *HADES* proves highly effective for this purpose.

Departing from evolving RL agents with high fitness, we here seek to develop agents that exhibit specific target behaviors not encoded in the environment's reward signal, thus remaining neutral to the agent's fitness. Specifically, we apply classifier‐free‐guidance at the genotypic level of *CHARLES‐D* to evolve RL‐agents with targeted phenotypic‐behavioral traits in their respective environment.

To validate our approach on a minimal yet descriptive example, we employ the cart‐pole system [35] (see Figure 7A), where a cart with a hinged pole moves sideways with the objective of maintaining the pole in a vertical position for as long as possible within a defined range. The fitness score corresponds to the total number of time steps $N_{s}\leq 500$ before the game termination, which occurs when either the pole‐angle exceeding $\phi_{\lim}=\pm 12^{\circ }$ or the cart moving beyond the boundaries $x_{\lim}=\pm 2.4$. The cart is controlled by an ANN (c.f., Supporting Information Section S7). The network processes four input parameters, i.e. the current position, x(s), velocity $\dot{x}\left(s\right)$, pole angle ϕ(s), and pole angular velocity $\dot{\phi }\left(s\right)$, and its outputs determines the cart's movement direction (left or right). The task is considered solved if the RL agent consistently achieves a fitness score of 500 across multiple episodes.

First, we evolve RL‐agents with maximum fitness using *HADES*, comparing its performance against mainstream methods such as CMA‐ES and a SimpleGA. As ANN architecture, we chose Recurrent Neural Networks [82] (RNN) with different layouts. The results depicted in Figure 7B demonstrate, that the *HADES* can solve the problem in as little as 3−4 generations, although RNNs can be tedious to train in RL applications, while CMA‐ES and the SimpleGA take longer by an order of magnitude; the population size was $N_{p}=256$, fitness scores were averaged over $N_{e}=16$ episodes, and we used elitism. We expand our comparison with an analysis of the *CHARLES‐D* method, conditioning on producing offspring with high fitness. The results demonstrate that even when operating under Fisher conditional optimization, the performance noticeably exceeds that of the *HADES*.

Next, we focus on controlling the cart's resting position $x^{\left(r\right)}=\frac{1}{N_{r}}\sum_{s=N_{s}-N_{r}}^{N_{s}}x\left(s\right)$, which expresses the average position where the cart stabilizes the pole during the final $N_{r}=100$ steps of an episode. We conduct ten independent *HADES* optimization trials, during, which we recorded both the ANN parameters and their corresponding behavioral outcomes $\left\{g_{i},x_{i}^{\left(r\right)}\right\}$. To predict the resting position based on the RL‐agent parameters, we train a deep ANN denoted as $f^{\left(r\right)}$, such that ${\hat{x}}_{i}^{\left(r\right)}=f^{\left(r\right)}\left(g_{i}\right)$. We aim to minimize the weighted mean‐square error $h\left[f_{i}\right]{\left|{\hat{x}}_{i}^{\left(r\right)}-x_{i}^{\left(r\right)}\right|}^{2}$, where $h[f_{i}]$ serves as the weighting factor (*c.f*., Supporting Information Sections S2.2 and S6.1). As shown in Figure 7C, the prediction accuracy is particularly strong for agents with high fitness of $f_{i}\approx 500$. Notably, these high‐fitness agents demonstrate resting positions that span the complete range of $x^{\left(r\right)}\in \left[-2,2\right]$.

Using the joint database of parameters and associated behavioral data $\left\{g_{i},x_{i}^{\left(r\right)}\right\}$ from earlier *HADES* evaluations, we train a generative DM “offline” on this genetic database, i.e., without utilizing the DM in any further optimization. We then use the DM to conditionally sample novel genotypes ${\hat{g}}_{\nu }$ that parameterize the behavior of RL‐agents to balance the pole at specific target locations $x_{c}=\left\{-2,-1,0,1,2\right\}$. The results in Figure 7D demonstrate, that the conditional sampling achieves good accuracy in biasing the corresponding RL‐agents toward exhibiting the desired target behavior.

Finally, we use the *CHARLES‐D* method to evolve an initially randomized population of ANN parameters for cart‐pole controllers toward solutions that exhibit specific target behaviors, namely balancing the pole either at resting positions $x_{c}=\left\{-0.5,0.5,1\right\}$. This is achieved without modifying the reward signal, but instead using conditional training of the generative DM on the ANN parameters $g_{i}$ and their associated mean resting positions $x_{i}^{\left(r\right)}$ measured during the evolutionary process. Offspring genotypes are then selectively generated by conditioning the DM on $c^{\left(r\right)}=x_{c}$ (see Supporting Information Sections S4 and S8 for details).

Notably, this experiment is performed independently of the database and pretrained DM discussed in Figure 7C,D. Rather, we begin with a randomized initial population and a randomly initialized DM, that is solely trained on parameters explored during the respectively evolving populations of cart‐pole agents, i.e., in an “online” mode. Figure 7E shows the measured resting positions $x_{i}^{\left(r\right)}$ for the three independent lineages with $x_{c}=-0.5,0.5$, or 1. These positions $x_{i}^{\left(r\right)}$ correspond to generated agents $g_{i}$ that achieved an average fitness score of 500 over $N_{e}=16$ consecutive episodes.

### 3.8 Behavioral Bias via Conditional Sampling can Reliably Solve Sparse‐Reward Environments

**FIGURE 8:**
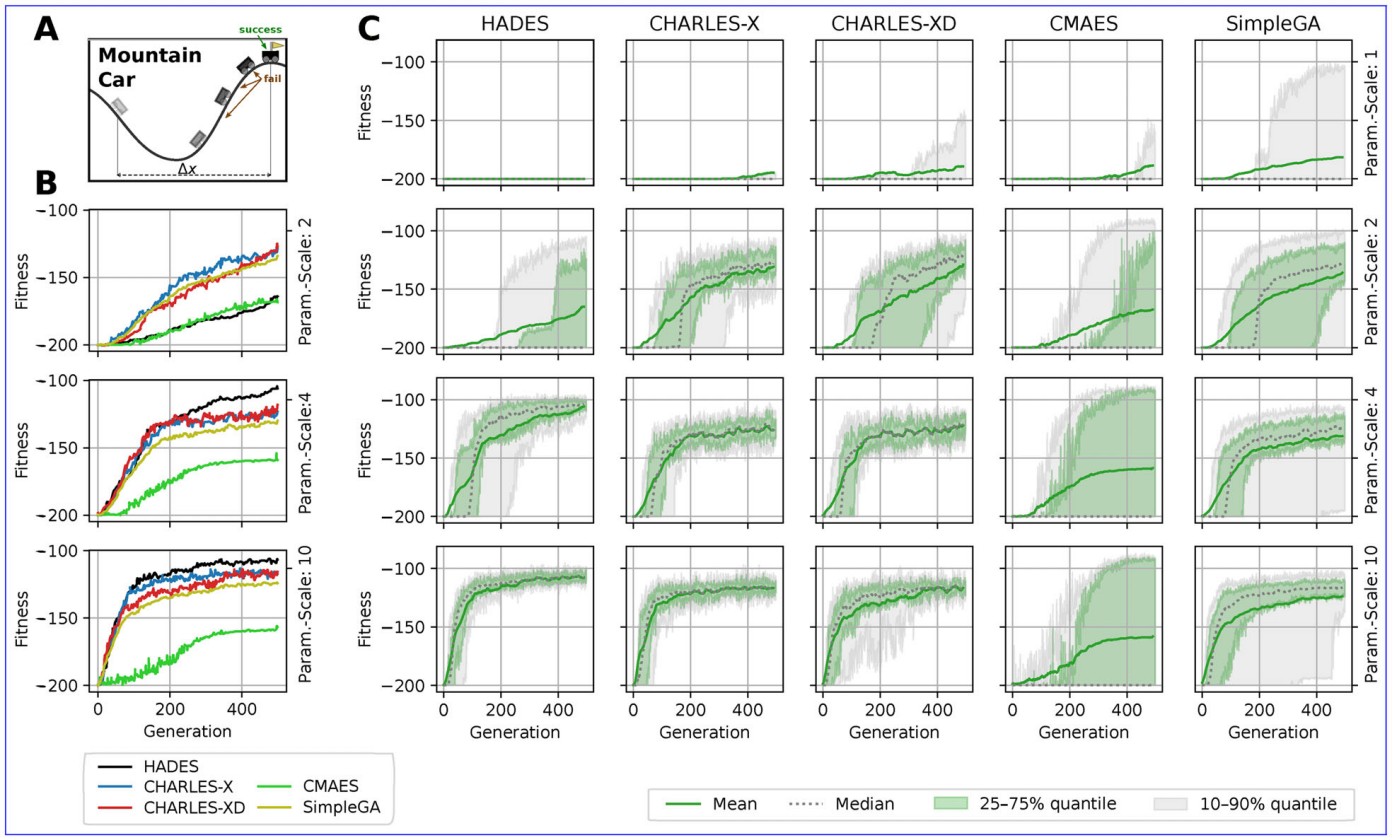
(A) Sketch of an RGRN‐based RL‐agent controlling the car in the MountainCar–v0 environment [83]. (B) Training performance of HADES, Δx‐conditional *CHARLES‐X*, Δx‐ and greedy fitness‐conditional *CHARLES‐XD*, CMA‐ES, and SimpleGA (color‐coded) for a 43‐parameter RGRN controller at different parameter scaling factors ($s_{g}=2,4,10$; rows) at a fixed evaluation budget per generation. Solid lines indicate the maximum fitness per generation, averaged over 20 independent evolving lineages. *CHARLES‐X* and *CHARLES‐XD* achieve rapid and reliable success, closely followed by the SimpleGA, while, in contrast, CMA‐ES shows erratic behavior across parameter scaling factors. HADES reaches high fitness at larger scaling factors but with reduced diversity. (C) Detailed fitness outcomes across 20 independent lineages for the same solvers (c.f., columns) and parameter scaling factors ($s_{g}=1,2,4,10$; rows); see text for details. Green solid lines mark the mean fitness, dashed gray lines the median, green shaded areas the interquartile range, and gray shaded areas the 10–90% range. Narrow bands of *CHARLES‐X* and *CHARLES‐XD* indicate consistent solutions across lineages, in contrast to the wide quantiles of CMA‐ES and the SimpleGA. All solvers maintain a population of $N_{p}=128$ candidate solutions across generations to ensure a fixed fitness evaluation budget at a given generation.

Below, we demonstrate that our conditional diffusion evolution (*CHARLES‐D*) can be used to enhance the search efficiency in reward‐sparse environments. By introducing conditions, we are able to guide the evolutionary search toward promising regions in the solution space without relying on reward‐shaping. As a case study, we consider the MountainCar‐v0 environment from OpenAI Gym [83]. In this task, a car must escape a valley moving to the left and right to build sufficient momentum to climb to the correct side of the hill, as illustrated in Figure 8A. The agent perceives a 2D state vector, comprising the position $x_{s}\in \left[-1.2,0.6\right]$ and velocity of the car ${\dot{x}}_{s}\in \left[-0.07,0.07\right]$ at each timestep s=1,⋯,200, and receives a penalty of $r_{s}=-1$ until the goal is reached, with a maximum episode length of 200 steps. Failure to reach the goal thus yields a cumulative reward (or fitness score) of $f_{i}=-200$. The only way an episode can terminate early is if the car successfully reaches the goal, in which case the score is $f_{i}=-N$, where N is the number of elapsed steps. This setting represents a reward‐sparse environment, where the only positive signal comes from complete task success.

Finding a solution to the MountainCar task is not trivial, as it requires global exploration of the agent's parameter space without any reward feedback on failure. This renders the problem especially difficult for naïve gradient‐based methods without random chance. We therefore benchmarked our methods, *HADES* and *CHARLES‐D*, against CMA‐ES, and SimpleGA on this environment.

Again, we use an ANN‐based RL controller to solve the task, which we parametrize via genetic encoding $g_{i}$ of a 43‐parameter RGRN architecture (see Supporting Information Section S9 for details), and use the cumulative reward as fitness score $f_{i}=\sum_{s}r_{s}$ for every candidate solution i. Since the problem exhibits a strong dependence on the scale of the agent parameters, we introduce a parameter scaling factor $s_{g}\in \left\{1,2,4,10\right\}$, which rescales the genetic parameters $g_{i}$ to the agent's ANN parameters ${\tilde{g}}_{i}=s_{g}g_{i}$. Larger scaling factors enable mutations to induce broader changes in behavior, thereby increasing the effective exploration radius in parameter space. As a result, search performance generally improves with (moderately) increasing $s_{g}$.

To ensure a fair comparison of the *HADES* and *CHARLES‐D* methods with CMA‐ES and SimpleGA, we fix the population size to $N_{p}=128$ for all solvers, sample the initial population $g_{i}$ from a normal distribution of STD $\sigma_{I}=1$, and conistently apply the same parameter scaling $g_{i}\to s_{g}g_{i}$ for all methods. Moreover, we explicitly refrain from elitism or crossover operations in *HADES* and *CHARLES‐D*, and do not use any weight decay for the genetic parameters in any solver; see Supporting Information Sections S4 and S9 for more details.

Our results depicted in Figure 8B–C show that both CMA‐ES and SimpleGA struggle to solve the task reliably, with SimpleGA showing surprisingly good performance: although several lineages eventually solve the task across 20 independent evolutionary optimization runs, many – if not most – agents remain stuck at the default $f_{i}=-200$ fitness score even after 500 generations, particularly at low parameter scaling factors ($s_{g}=1-2$). This is reflected in the low median fitness and wide spreads in the quantiles of their fitness distributions in Figure 8C. HADES shows similar behavior for low parameter scaling factors $s_{g}=1,2$, but shows remarkable (and reliable) performance for larger $s_{g}=4,10$.

By contrast, introducing conditional guidance with *CHARLES‐D* demonstratively improves performance across parameter scaling factors: To this end, we utilize classifier‐free guidance to condition the generation of novel offspring via the DM to deliberately maximize horizontal displacement $\Delta x_{i}=\left|\max\left(x_{s}\right)-\min\left(x_{s}\right)\right|$ of an agent i during an episode s=1,…,200. More specifically, we jointly train the DM on genotypic parameters and behavioral traits $\left\{g_{i},\Delta x_{i}\right\}$, and sample novel offspring with successively larger conditions $c^{\left(\Delta x\right)}≳\max\left(\left\{\Delta x_{i}\right\}\right)$, find details in the Supporting Information Section S9. As sketched in Figure 8A, success requires coordinated back‐and‐forth oscillations of the car to build momentum; conditioning on larger $\Delta x_{i}$ directly targets this behavioral feature. This so‐termed *CHARLES‐X* strategy reliably identifies successful solutions across a broad range of scaling factors. By favoring agents (which initially all exhibit the default cumulative reward of ‐200) with large horizontal spread, *CHARLES‐X* increases car mobility across evolutionary generations and thus enables not only faster but reliable task completion: In Figure 8C, the narrow interquartile and 10—90% ranges of *CHARLES‐X* and *XD* (and for larger $s_{g}$ values, also *HADES*) demonstrate consistent success across lineages, while solutions from CMA‐ES and GA remain highly variable.

We further explored an additional *CHARLES‐XD* conditioning strategy, combining greedy fitness‐based and Δx‐based conditions. Especially at low parameter scaling factors, this variant shows increased exploration performance compared to *CHARLES‐X*, and demonstrates strong and reliable convergence. For larger scaling factors, vanilla *HADES* also shows competitive performance with even higher absolute fitness evaluations compared to *CHARLES‐X*, which we attribute to the slightly conflicting objectives of *CHARLES‐X* and *XD* to simultaneously maximize fitness and Δx. The improved performance of *HADES* comes at the cost of reduced population diversity compared to *CHARLES‐X*. Conditional exploration thus represents a promising warmup phase, which can subsequently be finetuned.

In all cases (*HADES*, *CHARLES‐X*, and *CHARLES‐XD*), the DMs were retrained only every eighth generation, ensuring that computational costs remained comparable to SimpleGA and CMA‐ES in terms of CPU hours (total runtimes only deviate by ≈10%). We thus demonstrate that even with the overhead of successively training a DM on a constantly refined heuristic dataset buffer, we achieve analogous computational performance and superior explorative behavior, even on reward‐sparse environments (see also the Methods section for a statement on Computational Efficiency and Sample Complexity of our method, and the Supporting Information Section S9).

The Supporting Information Section S10 demonstrates that our approach can also be utilized efficiently to more complex problems, such as the lunar‐lander rocket environment [83]. More specifically, we demonstrate that conditional sampling through *CHARLES‐D* can be used to evolve targeted behavior such as minimizing fuel‐usage after ground‐contact – a typical pit‐fall in this environment – again without reward‐shaping.

These results underscore two key advantages of conditional diffusion evolution. In all above cases, the generative process demonstrates clear bias in generating genotypic parameters that encode specific functional behaviors at the phenotypic level that is neutral to the agents' fitness scores. Beyond intriguing potential biological implications (discussed below), our *CHARLES‐D* method offers a powerful form of regularization via conditional guidance, enabling efficient multi‐objective optimization across various problem domains without requiring complex reward shaping techniques [54]. Through classifier‐free‐guidance conditioning of the generative process at genotypic parameter level, we direct evolutionary search toward parameter space regions that yield desired phenotypical behaviors. Importantly, the same mechanism can also be used to exclude undesired traits or solution classes, effectively implementing a contrastive form of evolutionary learning. *CHARLES‐D* effectively channels conditional information (specifically, desired behavior) across multiple layers of abstraction, from the generative phase at the genotypic level to actual behavior of ANN‐based autonomous RL‐agents at the phenotypic level in physically realistic environments. This bridging occurs through joint training of a generative model on governing genotypic parameters and their associated phenotypic traits. DMs capture the developmental layer between genotypes and phenotypes [25] by learning how to gradually generate parameters that conform to conditionally encoded phenotypic traits. In essence, DMs encapsulate the computationally irreducible nature [51] of the developmental process by learning how to actually compute high‐quality samples through step‐wise error correction mechanisms conditional to specified target features, thereby forming an associative memory of domain‐specific generative processes.

## 4 Discussion

Our work, alongside a complementary contribution [48], establishes a connection between diffusion models (DMs) and evolutionary algorithms (EAs) through shared underlying conceptual and mathematical foundations: DMs can be viewed as evolutionary processes in disguise. In this paper, we demonstrate that deep‐learning based DMs can effectively serve as efficient generative models in EAs, enhancing genotypic recombination operations. Rather than relying on pretraining DMs with large general datasets, we continuously refine DMs using heuristically acquired, high‐quality parameters from evolutionary process. This iterative refinement of the DM's generative process, based on the most recent evolutionary evidence in biology, enables the DM to be adaptive to evolutionary changes.

DMs leverage Artificial Neural Network (ANN) to sample novel data points conforming to a target parameter distributions. Given their status as universal function approximators [73], ANNs excel at learning complex correlations within arbitrary datasets, making them ideal for identifying subtle correlations in parameters of evolutionary processes. Through iterative refinement of the DM's generative process using heuristically acquired high‐quality data from evolutionary processes, we propose the novel Heuristically Adaptive Diffusion‐Model Evolutionary Strategy (*HADES*) method. We contrast our method's performance with mainstream EA techniques across various numerical optimization scenarios and report significant improvements in adaptability to changing environments while maintaining reliable convergence to target solutions.

DMs augment evolutionary processes with unconventional (epigenetic) memory [52, 53]: Using elite buffer datasets collected across generations or persistent traits in constantly retrained DMs allows the generative process to utilize previously experienced information, thus enabling faster adaptation in changing environments. This memory capability proves especially crucial when objective functions are computationally expensive. Our findings confirm that maintaining a memory buffer enhances both search result quality and diversity.

Moreover, via classifier‐free‐guidance techniques [33], we can utilize conditional sampling in DMs to directly bias the evolutionary search dynamics, steering it towards regions in the parameter space that exhibit desired target traits. This leverages multi‐objective optimization without the need for complex reward shaping [54] or curricula learning techniques [61]. By conditioning the DM's generative phase across successive generations, we demonstrate control over: (i) the search dynamics in the parameter‐space, (ii) sampling offspring generations with specific fitness distributions similar to inverse reinforcement learning [65] and in addition controlling the diversity or greediness of the population, (iii) biasing the search towards desired phenotypic traits that are neutral to the problem's fitness score, and (iv) even to explicitly maintain population diversity through novelty‐ or diversity‐conditioning. This conditional sampling mirrors image‐ or video‐generation techniques that are controlled by custom text‐inputs [40, 41]. Our Conditional, Heuristically‐Adaptive Regularized Evolutionary Strategy through Diffusion (*CHARLES‐D*) method introduces conditional sampling during optimization, effectively constraining search result qualities via custom control parameters defined independently to the task's objective function. Thus, our approach represents, to the best of our knowledge, the first “Talk to your Optimizer” [84] application.

In that way, DMs demonstratively outperform the generative models of mainstream genetic algorithms in flexibility, versatility, and control over the search dynamics: With *HADES* and *CHARLES‐D* we can successfully solve high‐dimensional complex optimization problems, and even bias the search‐dynamics toward desired behavior, out‐competing mainstream approaches. Through an epigenetic dataset memory buffer, we can even dynamically condition the search behavior to revisit promising, previously experienced parameter space regions, similar to an associative memory [14, 50, 63]. Moreover, such unconventional memory properties enable specific conditioning of the generative process across evolutionary generations to actively promote novelty and diversity, as effective novelty search requires memory capability [85]. Eventually, both *HADES* and *CHARLES‐D* solutions show remarkable diversity, particularly in reinforcement learning tasks.

Several limitations of the current approach can be extended in future work: While biological evolution operates with discrete chemical building blocks, we have applied our algorithm only to continuous parameter spaces. Although, we utilized several DM architectures and observed largely architecture agnostic search dynamics with sufficiently large DM latent spaces, the balance between population size, memory buffer dataset size, training epochs, learning rate schedule, and the number of evolutionary generations between DM retraining may affect search dynamics. High‐quality data proves crucial for both diversity and convergence rates. Careful consideration is necessary to balance exploration‐exploitation behavior, particularly regarding diversity measures. Questions remain about whether to retrain a single DM across generations or train novel DMs from scratch using updated heuristics. Moreover, the ratio of the active population to the total possible solutions in the parameter space needs to be explored. Although both *HADES* and *CHARLES‐D* show great promise for improved multimodal optimization, we note that specialized niching and quality‐diversity algorithms such as RS‐CMSA [86, 87] or MAP‐Elites [88] represent powerful alternatives; however, these approaches are highly problem‐dependent and require extensive tuning. A comprehensive comparison with such methods is therefore left for future work. While DM training introduces additional overhead, this cost is either negligible to computationally heavy fitness evaluations (such as in RL tasks or protein design) or can be mitigated in practice by parallelizing both evaluations and training on separate compute nodes using a shared dataset buffer.

Intriguingly, our methods demonstrate several aspects relevant to the emerging field of Diverse Intelligence [89], in which cognitive and epistemic dynamics are studied in a very wide range of embodiments and spatio‐temporal scales. For example, the DM's generative process applies successive denoising steps, transforming initially random input into successively refined highly‐correlated, high‐quality parameter output conforming to a training dataset. This iterative denoising process represents a perception‐action cycle of genotypic parameters similar to [90, 91] and in Neural Cellular Automata [92, 93], driving (i.e., poorly adapted) genotypes toward statistically more probable (i.e., better adapted) parameter space regions. Furthermore, this process can demonstratively learn from experience and, through conditioning, responds to external stimuli that are orthogonal, or neutral to a fitness score. This perspective reframes the evolutionary process as an active learning system [12]. Thus, an evolutionary process's classification as being a variational or transformational depends on the observer's perspective: individuals experience learning as transformational, refining an internal world model while maintaining their “identity”. From an evolutionary or ecosystem perspective [94], individuals represent temporary “experiments”, while interacting species form a transformational learning system conditional to particular environmental constraints [7, 10, 11, 12, 13, 14, 63].

### 4.1 A Paradigm Shift: Diffusion Models Sample Gene‐Expressions Rather Than Genomic Parameters

DMs have recently been identified as associative memories [50, 95], and, in a complementary work, as evolutionary algorithms [48]. Correspondingly, evolution can be understood as a form of Hebbian‐learning [7, 11, 12, 13, 14, 63]. Evolutionary learning operating at the bio‐molecular level, maintaining DNA‐based associative memory with self‐regulatory capabilities in expressing protein sequences that constrain functionality of their host cell. This perspective presents DNA as a generative model that initializes the developmental process of an organism rather than a direct blue‐print of the latter [25, 26, 27, 96]. In this process, genes are expressed in modular response to a host cell's configuration, internal state, and environment.

Our research demonstrated that DMs, when trained on generating functional parameters conditionally to associated behavioral features, can be used to selectively evolve agent policies exhibiting targeted behavior that is neutral to the fitness score. We explicitly show this in experiments with a cart‐pole environment where agents balance a pole vertically, but conditional at specific target locations: The same DM model can generate distinct control parameters for a cart‐pole agent, enabling pole stabilization at different desired locations, e.g., $x_{A}$, or $x_{B}\neq x_{A}$, simply by conditioning the DM's generative denoising process on policy A or B. Moreover, we can smoothly transform between these policies by exchanging the respective conditions: When shifting from policy A to B, the DM adapts the agent's parameters accordingly, representing an unconventional form of behavioral control through parameter reconfiguration. This mechanism closely parallels how gene‐regulatory networks dynamically reconfigure cell functionality in response to internal or external stimuli [97, 98, 99, 100, 101, 102].

Viewing this through the lens of recent interpretations of the genome as a generative model [25, 26, 27, 96], we hypothesize that the DM used in our methods literally represents a lineage's evolving genome, including its ability to self‐regularize and utilize gene expressions that reconfigure phenotype functionality based on environmental constraints (as demonstrated by conditions A or B in the above cart‐pole example). This suggests that the parameters in our work do not represent genotypic representations but gene expressions encoding phenotype functionality and behavior, similar to the ways in which gene expressions encode protein sequences that control cellular behavior. The complete genome (i.e., represented by the DM) contains much richer information, including an associative memory on gene expression modalities that is accessible on demand. We propose that generative DMs are good models for self‐regulatory, generative DNA, sampling gene‐expressions rather than genotypic representations

Thus, we believe that our model is much closer to biology than previous evolutionary methods, by representing DNA as a generative model with associative memory, surpassing previous evolutionary methods in detail and functionality. This representation enables our evolutionary search strategy to specifically respond to external conditions and generates problem‐specific parameter expressions through conditional denoising processes that are fundamentally rooted in non‐equilibrium physics [39]. Such capabilities allow dynamic reconfiguration of phenotype behavior, closely mirroring biological systems' adaptability, in turn promoting intriguing technological innovations.

## 5 Method

### 5.1 Generative Diffusion Models (DMs)

DMs [36, 37, 38, 39] iteratively transform normal distributed samples into structured data points that conform to a training dataset X. This is realized in two‐phases: first, a (diffusive) forward process progressively corrupts data $x_{t}=\sqrt{\alpha_{t}}x_{0}+\sqrt{1-\alpha_{t}}\varepsilon$ by iteratively blending data $x_{0}∼p_{X}\in R^{D}$ with noise $\varepsilon ∼N^{D}$ over time t=(0,T] with monotonously decreasing $\alpha_{t}:\alpha_{0}=1,\alpha_{T}=0$. Second, a model $\varepsilon_{\theta }\left(\cdot \right)$ learns to predict this blended noise $\varepsilon_{\theta }\left(x_{t},t\right)={\hat{\varepsilon }}_{t}$ to refine initially noisy samples $x_{T}$ through iterative denoising $x_{t-1}=\sqrt{\alpha_{t-1}}{\hat{x}}_{0}+\sqrt{1-\alpha_{t-1}-\sigma_{t}^{2}}{\hat{\varepsilon }}_{t}+\sigma_{t}w$ during the (generative) reverse process, with ${\hat{x}}_{0}=\left(x_{t}-\sqrt{1-\alpha_{t}}{\hat{\varepsilon }}_{t}\right)/\sqrt{\alpha_{t}}$, until generated samples $x_{0}$ conform to the training data X; details in Supporting Information Section S2.2.

### 5.2 Evolutionary Algorithms (EAs)

EAs [17, 18, 19, 20] are heuristic optimization techniques inspired by natural selection. In EAs, a set (population) $G_{\tau }=\left\{g_{\tau ,1},\text{…}\;,g_{\tau ,N_{P}}\right\}$ of $N_{P}$ genotypic parameters $g_{\tau ,i}\in R^{D}$ is maintained and modified over successive iterations (generations) τ to optimize an objective function or fitness score, $f_{\tau ,i}=f\left(g_{\tau ,i}\right):R^{D}\to R$. Typically starting with (i) randomized initial parameters $g_{0,i}∼N^{D}$, (ii) high‐fitness solutions ${\bar{g}}_{\tau ,i}$ are selected for reproduction from the current generation τ: The reproduction operation (iii) often encompasses genetic recombination and mutation operations to create novel offspring, which can also be done by sampling from a generative model $g_{\tau +1,o}∼p_{{\bar{G}}_{\tau }}$ of high‐quality samples ${\bar{G}}_{\tau }$. High‐quality offspring successively (iv) replacing low‐quality solutions navigates a population toward high‐fitness regions in the parameter space across successive generations (ii‐iv) [31, 77].

### 5.3 Heuristically Adaptive Diffusion‐Model Evolutionary Strategy (*HADES*)

HADES utilizes DMs as generative models for the reproduction operation (iii) in EAs, departing from inductively biased approaches [22]. By associating a comprehensive dataset $X=\{G_{1},\text{…}\;,G_{\tau }\}$ of historical data $G_{\tau^{\prime }<\tau }$ and successively refined populations $G_{\tau }$ with corresponding fitness‐scores $f_{\tau ,i}$, we can train a powerful generative DM, capturing subtle correlations in genetic data across entire evolutionary lineages, to sample diverse high‐quality offspring $g_{\tau +1,o}∼p_{\bar{X}}$ with large probability; details in Supporting Information.

### 5.4 Conditional, Heuristically‐Adaptive ReguLarized Evolutionary Strategy Through Diffusion (*CHARLES‐D*)

Through classifier‐free guidance [33], we can train the DM in *HADES* to associate genotypic $g_{i}$ and fitness data $f_{i}$ additional with features $c_{i}=c\left(\cdot \right)$ capturing, e.g., genotypic, phenotypic, population‐wide, or fitness‐specific traits. Extending the DM's input with the evaluated features $\varepsilon_{\theta }\left(x_{t},t\right)\to \varepsilon_{\theta }\left(x_{t},t,c\right)$ allows us to train the DM jointly on data $g_{i}\equiv x_{0}$ and corresponding features $c_{i}=c\left(g_{i}\right)$, where $x_{t}$ is the diffused genetic data. During reproduction (iii), we then sample high‐quality offspring data $g_{\tau +1,o}∼p_{\bar{X}|c^{\left(T\right)}}$ conditional to target features $c^{\left(T\right)}$, thereby effectively biasing the evolutionary process to obtain $c\left(g_{\tau +1,o}\right)\approx c^{\left(T\right)}$.

#### 5.4.1 Hyperparameters

In all our investigations, we use simple feed‐forward architectures for our DMs, typically with two hidden layers and 24‐324 hidden units per layer. To improve readability, we explicitly list the simulation parameters of all presented experiments and details about our methods in the Supporting Information Sections S3 and S4.

#### 5.4.2 Computational Efficiency and Sample Complexity

While deep‐learning‐based DMs introduce computational overhead compared to traditional EAs, the critical metric in many real‐world applications is the number of fitness evaluations rather than wall‐clock time. This distinction proves particularly crucial in domains where fitness evaluation dominates computational cost, such as wet‐lab experiments, physical simulations, or reinforcement learning environments. Our *HADES* and *CHARLES‐D* methods demonstrate competitive sample efficiency across diverse optimization tasks.

In the cart‐pole environment (Figure 7), *HADES* consistently solves the task in 3–4 generations with a population size of $N_{p}=256$, requiring approximately 1,024 fitness evaluations (each consisting of $N_{e}=16$ episode rollouts). In contrast, CMA‐ES and SimpleGA require an order of magnitude more evaluations to achieve comparable performance. For the more complex lunar‐lander task (see Supporting Information Figure S9), both *HADES* and Fisher‐type *CHARLES‐D* solve the environment in ≈5 generations in the best case, corresponding to ≈1,280 evaluations with $N_{p}=256$. These results highlight the effectiveness of our methods in solving complex tasks within a limited evaluation budget, making them highly suitable for data‐limited domains.

The computational cost of training the DM scales as $O(N_{E}\cdot N_{p}\cdot D)$ per generation, where $N_{E}$ represents training epochs, $N_{p}$ the population size, and D the parameter dimension. However, this cost can be amortized through several mechanisms:

1. The population‐based nature of our approach enables parallel fitness evaluation while, in addition, allows a decoupling of the DM training on a separate node, effectively hiding DM training latency when evaluations are distributed.
2. The epigenetic memory buffer allows the DM to leverage historical data, reducing the number of evaluations required in subsequent generations.
3. The framework is amenable to transfer learning, where a pre‐trained DM could provide warm‐start capabilities for related tasks, potentially reducing future convergence time.

Crucially, for optimization problems where gradient‐based methods like backpropagation (BP) prove intractable including non‐differentiable objectives, discrete parameter spaces, or multi‐modal landscapes the relevant comparison lies not with BP but with other black‐box optimizers. In such domains, the enhanced exploration capabilities and conditional control offered by *HADES* and *CHARLES‐D* often outweigh the computational overhead, particularly when fitness evaluation constitutes the dominant cost. Moreover, unlike traditional EAs that discard historical information, our methods maintain an evolving dataset buffer that captures the exploration history, effectively trading computation for sample efficiency – a favorable exchange in evaluation‐limited regimes.

## Author Contributions

B.H., Y.Z., H.H., and M.L. contributed to writing, review, and editing. B.H., Y.Z., and H.H. contributed equally to this work.

## Conflicts of Interest

M.L. is a co‐founder and scientific advisor to Astonishing Labs and owns equity in the company. Astonishing Labs partially funded this work through a sponsored research agreement with Tufts University. The company also has option rights to a patent application filed by Tufts University related to the subject matter and potential applications presented in this paper. The authors declare no other competing interests.

## Supporting information


**Supporting File 1**: advs72244‐sup‐0001‐SuppMat.pdf.

**Supporting File 2**: advs72244‐sup‐0002_conditional_data.zip.pdf.

**Supporting File 3**: advs72244‐sup‐0003_conditional_data‐README.boxnote.

## Data Availability

Computational protocols and numerical data that support the findings of this study are shown in this article, and the Supporting Information. A GitHub repository containing the source code implementation of the method described in this paper is available at https://github.com/bhartl/CondEvo/tree/main/condevo, providing additional resources and examples for reproducibility and further experimentation.

## References

[1] C. Darwin , “The Origin of Species, 1859‐1959,” Bios 30, no. 2 (1959): 67–72.

[2] R. Dawkins , The Selfish Gene (Oxford University Press, 2016).

[3] E. R. Kandel , Principles of Neural Science, Fifth Edition (McGraw Hill Professional, 2013).

[4] A. C. Courville , N. D. Daw , and D. S. Touretzky , “Bayesian Theories of Conditioning in a Changing World,” Trends in Cognitive Sciences 10, no. 7 (2006): 294–300. 16793323 10.1016/j.tics.2006.05.004

[5] J. H. Holland , Emergence: From Chaos to Order (OUP Oxford, 2000).

[6] P. Dayan and L. F. Abbott , Theoretical Neuroscience: Computational and Mathematical Modeling of Neural Systems, Ser. Computational neuroscience (Massachusetts Institute of Technology Press, 2001).

[7] R. Watson and M. Levin , “The Collective Intelligence of Evolution and Development,” Collective Intelligence 2, no. 2 (2023): 26339137231168355.

[8] V. Vanchurin , Y. I. Wolf , M. I. Katsnelson , and E. V. Koonin , “Toward a Theory of Evolution as Multilevel Learning,” Proceedings of the National Academy of Sciences 119, no. 6 (2022): e2120037119. 10.1073/pnas.2120037119 PMC8833143 35121666

[9] M. Levin , “Technological Approach to Mind Everywhere: An Experimentally‐Grounded Framework for Understanding Diverse Bodies and Minds,” Frontiers in Systems NeuroScience 16 (2022): 768201. 35401131 10.3389/fnsys.2022.768201 PMC8988303

[10] R. A. Watson , M. Levin , and C. L. Buckley , “Design for an Individual: Connectionist Approaches to the Evolutionary Transitions in Individuality,” Frontiers in Ecology and Evolution 10 (2022): 823588.

[11] K. Kouvaris , J. Clune , L. Kounios , M. Brede , and R. A. Watson , “How Evolution Learns to Generalise: Using the Principles of Learning Theory to Understand the Evolution of Developmental Organisation,” PLoS Computational Biology 13, no. 4 (2017): e1005358. 28384156 10.1371/journal.pcbi.1005358 PMC5383015

[12] R. A. Watson and E. Szathmáry , “How Can Evolution Learn?” Trends in Ecology & Evolution 31, no. 2 (2016): 147–157. 26705684 10.1016/j.tree.2015.11.009

[13] R. A. Watson , R. Mills , C. Buckley , et al., “Evolutionary Connectionism: Algorithmic Principles Underlying the Evolution of Biological Organisation in Evo‐Devo, Evo‐Eco and Evolutionary Transitions,” Evolutionary Biology 43 (2016): 553–581. 27932852 10.1007/s11692-015-9358-z PMC5119841

[14] D. A. Power , R. A. Watson , E. Szathmáry , et al., “What Can Ecosystems Learn? Expanding Evolutionary Ecology with Learning Theory,” Biology Direct 10 (2015): 1–24. 26643685 10.1186/s13062-015-0094-1 PMC4672551

[15] G. E. Hinton and S. J. Nowlan , “How Learning Can Guide Evolution,” Complex Systems 1, no. 3 (1987): 495–502.

[16] J. M. Baldwin , “A New Factor in Evolution,” Diacronia (2018): 1–13.

[17] P. A. Vikhar , “Evolutionary Algorithms: A Critical Review and Its Future Prospects,” in 2016 International Conference on Global Trends in Signal Processing, Information Computing and Communication (ICGTSPICC) (IEEE, 2016), 261–265.

[18] D. E. Golberg , Genetic Algorithms in Search, Optimization, and Machine Learning (Addison‐Wesley, 1989).

[19] J. J. Grefenstette , “Genetic Algorithms and Machine Learning,” in Proceedings of the Sixth Annual Conference on Computational Learning Theory (1993): 3–4.

[20] J. H. Holland , Adaptation in Natural and Artificial Systems: An Introductory Analysis with Applications to Biology, Control, and Artificial Intelligence (MIT Press, 1992).

[21] S. Katoch , S. S. Chauhan , and V. Kumar , “A Review on Genetic Algorithm: Past, Present, and Future,” Multimedia Tools and Applications 80, no. 5 (Oct. 2020) 8091–8126, 10.1007/s11042-020-10139-6. 33162782 PMC7599983

[22] N. Hansen and A. Ostermeier , “Completely Derandomized Self‐Adaptation in Evolution Strategies,” Evolutionary Computation 9, no. 2 (2001): 159–195. 11382355 10.1162/106365601750190398

[23] C. L. Buckley , T. Lewens , M. Levin , B. Millidge , A. Tschantz , and R. A. Watson , “Natural Induction: Spontaneous Adaptive Organisation Without Natural Selection,” Entropy 26, no. 9 (2024), https://www.mdpi.com/1099‐4300/26/9/765. 10.3390/e26090765 PMC11431681 39330098

[24] P. McMillen and M. Levin , “Collective Intelligence: A Unifying Concept for Integrating Biology Across Scales and Substrates,” Communications Biology 7, no. 1 (Mar. 2024), 10.1038/s42003-024-06037-4. PMC10978875 38548821

[25] M. Levin , “Darwin's Agential Materials: Evolutionary Implications of Multiscale Competency in Developmental Biology,” Cellular and Molecular Life Sciences 80, no. 6 (2023): 142. 37156924 10.1007/s00018-023-04790-z PMC10167196

[26] B. Hartl and M. Levin , “What Does Evolution Make? Learning in Living Lineages and Machines,” Trends in Genetics 41, no. 6 (Jun. 2025): 480–496, 10.1016/j.tig.2025.04.002. 40500652

[27] K. J. Mitchell and N. Cheney , “The Genomic Code: The Genome Instantiates a Generative Model of the Organism,” Trends in Genetics 41, no. 6 (June 2025): 462–479, 10.1016/j.tig.2025.01.008. 39934051

[28] G. Pezzulo and M. Levin , “Re‐Membering the Body: Applications of Computational Neuroscience to the Top‐Down Control of Regeneration of Limbs and Other Complex Organs,” Integrative Biology 7 (2015): 1487–1517. 26571046 10.1039/c5ib00221d PMC4667987

[29] G. Pezzulo and M. Levin , “Top‐Down Models in Biology: Explanation and Control of Complex Living Systems Above the Molecular Level,” Journal of The Royal Society Interface 13 (November 2016), 20160555. 27807271 10.1098/rsif.2016.0555 PMC5134011

[30] C. Fields and M. Levin , “Regulative Development as a Model for Origin of Life and Artificial Life Studies,” Biosystems 229 (2023): 104927, https://www.sciencedirect.com/science/article/pii/S0303264723001028. 37211257 10.1016/j.biosystems.2023.104927

[31] B. Hartl , S. Risi , and M. Levin , “Evolutionary implications of self‐assembling cybernetic materials with collective problem‐solving intelligence at multiple scales,” Entropy 26, no. 7 (2024): 532. 39056895 10.3390/e26070532 PMC11275831

[32] L. Shreesha and M. Levin , “Cellular competency during development alters evolutionary dynamics in an artificial embryogeny model,” Entropy 25, no. 1 (2023), https://www.mdpi.com/1099‐4300/25/1/131. 10.3390/e25010131 PMC9858125 36673272

[33] J. Ho and T. Salimans , “Classifier‐free diffusion guidance,” in NeurIPS 2021 Workshop on Deep Generative Models and Downstream Applications (2021), https://openreview.net/forum?id=qw8AKxfYbI.

[34] R. S. Sutton and A. G. Barto , Reinforcement Learning: An Introduction, vol. 1, no. 1 (MIT press Cambridge, 1998).

[35] A. G. Barto , R. S. Sutton , and C. W. Anderson , “Neuronlike adaptive elements that can solve difficult learning control problems,” IEEE transactions on systems, man, and cybernetics 13, no. 5 (1983): 834–846.

[36] P. Dhariwal and A. Nichol , “Diffusion models beat gans on image synthesis,” in Advances in Neural Information Processing Systems, M. Ranzato , A. Beygelzimer , Y. Dauphin , P. Liang , and J. W. Vaughan , Eds., vol. 34 (Curran Associates, Inc., 2021), 8780–8794, https://proceedings.neurips.cc/paper_files/paper/2021/file/49ad23d1ec9fa4bd8d77d02681df5cfa‐Paper.pdf.

[37] J. Ho , A. Jain , and P. Abbeel , “Denoising diffusion probabilistic models,” in Advances in Neural Information Processing Systems, H. Larochelle , M. Ranzato , R. Hadsell , M. Balcan , and H. Lin , Eds., vol. 33 (Curran Associates, Inc., 2020), 6840–6851, https://proceedings.neurips.cc/paper_files/paper/2020/file/4c5bcfec8584af0d967f1ab10179ca4b‐Paper.pdf.

[38] J. Song , C. Meng , and S. Ermon , “Denoising diffusion implicit models,” in International Conference on Learning Representations (2021), https://openreview.net/forum?id=St1giarCHLP.

[39] J. Sohl‐Dickstein , E. Weiss , N. Maheswaranathan , and S. Ganguli , “Deep unsupervised learning using nonequilibrium thermodynamics,” in International conference on machine learning (PMLR, 2015): 2256–2265.

[40] R. Rombach , A. Blattmann , D. Lorenz , P. Esser , and B. Ommer , “High‐resolution image synthesis with latent diffusion models,” in Proceedings of the IEEE/CVF conference on computer vision and pattern recognition (IEEE, 2022): 10 684–10 695.

[41] T. Brooks , B. Peebles , C. Holmes , et al., “Video Generation Models as World Simulators,” (2024).

[42] J. Jumper , R. Evans , A. Pritzel , et al., “Highly Accurate Protein Structure Prediction With AlphaFold,” Nature 596, no. 7873 (July 2021): 583–589, 10.1038/s41586-021-03819-2. 34265844 PMC8371605

[43] X. Yan and Y. Jin , “EmoDM: A Diffusion Model for Evolutionary Multi‐Objective Optimization,” (2024), https://arxiv.org/abs/2401.15931.

[44] C. F. Higham , D. J. Higham , and P. Grindrod , “Diffusion Models for Generative Artificial Intelligence: An Introduction for Applied Mathematicians,” (2023), https://arxiv.org/abs/2312.14977.

[45] S. Krishnamoorthy , S. M. Mashkaria , and A. Grover , “Diffusion Models for Black‐Box Optimization,” in Proceedings of the 40th International Conference on Machine Learning , ser. Proceedings of Machine Learning Research, A. Krause , E. Brunskill , K. Cho , B. Engelhardt , S. Sabato , and J. Scarlett , Eds., vol. 202 (PMLR, 23–29 Jul 2023): 17 842–17 857, https://proceedings.mlr.press/v202/krishnamoorthy23a.html.

[46] W. Peebles , I. Radosavovic , T. Brooks , A. A. Efros , and J. Malik , “Learning to Learn With Generative Models of Neural Network Checkpoints,” (2022), https://arxiv.org/abs/2209.12892.

[47] E. Alonso , A. Jelley , V. Micheli , et al., “Diffusion for World Modeling: Visual Details Matter in Atari,” in The Thirty‐Eighth Annual Conference on Neural Information Processing Systems (2024), https://openreview.net/forum?id=NadTwTODgC.

[48] Y. Zhang , B. Hartl , H. Hazan , and M. Levin , “Diffusion Models Are Evolutionary Algorithms,” in The Thirteenth International Conference on Learning Representations, ICLR 2025, Singapore, April 24–28, 2025 (OpenReview.net, 2025), https://openreview.net/forum?id=xVefsBbG2O.

[49] H. B. Callen and T. A. Welton , “Irreversibility and Generalized Noise,” Physical Review 83 (Jul 1951): 34–40, 10.1103/PhysRev.83.34.

[50] L. Ambrogioni , “In Search of Dispersed Memories: Generative Diffusion Models Are Associative Memory Networks,” Entropy 26, no. 5 (2024): 381, 10.3390/e26050381. 38785630 PMC11119823

[51] S. Wolfram , A New Kind of Science (Wolfram Media, 2002), https://www.wolframscience.com.

[52] E. Jablonka and G. Raz , “Transgenerational Epigenetic Inheritance: Prevalence, Mechanisms, and Implications for the Study of Heredity and Evolution,” The Quarterly Review of Biology 84 (06 2009): 131–176, http://www.blc.arizona.edu/courses/schaffer/449/Epigenetics/Transgen%20Effects/Jablonka%20Raz%20Transgen%20Epigen.pdf. 19606595 10.1086/598822

[53] E. Jablonka , “The Evolutionary Implications of Epigenetic Inheritance,” Interface Focus 7 (08 2017): 20160135. 28839916 10.1098/rsfs.2016.0135 PMC5566804

[54] A. Y. Ng , D. Harada , and S. J. Russell , “Policy Invariance Under Reward Transformations: Theory and Application to Reward Shaping,” in *Proceedings of the Sixteenth International Conference on Machine Learning*, ser. ICML '99 (Morgan Kaufmann Publishers Inc., 1999), 278–287.

[55] G. P. Wagner , M. Pavlicev , and J. M. Cheverud , “The Road to Modularity,” Nature Reviews Genetics 8, no. 12 (2007): 921–931, 10.1038/nrg2267. 18007649

[56] G. Schlosser and G. P. Wagner , Modularity in Development and Evolution (Chicago, IL: University of Chicago Press, July 2004).

[57] R. Calabretta , A. D. Ferdinando , G. P. Wagner , and D. Parisi , “What Does It Take to Evolve Behaviorally Complex Organisms?,” Biosystems 69, no. 2 (2003): 245–262, https://www.sciencedirect.com/science/article/pii/S0303264702001405. 12689732 10.1016/s0303-2647(02)00140-5

[58] K. H. Ten Tusscher and P. Hogeweg , “Evolution of Networks for Body Plan Patterning; Interplay of Modularity, Robustness and Evolvability,” PLoS Computational Biology 7, no. 10 (2011): e1002208, 10.1371/journal.pcbi.1002208. 21998573 PMC3188509

[59] G. P. Wagner and L. Altenberg , “Perspective: Complex Adaptations and the Evolution of Evolvability,” Evolution 50, no. 3 (1996): 967–976, http://www.jstor.org/stable/2410639. 28565291 10.1111/j.1558-5646.1996.tb02339.x

[60] I. Sutskever , J. Martens , G. Dahl , and G. Hinton , “On the Importance of Initialization and Momentum in Deep Learning,” in *Proceedings of the 30th International Conference on Machine Learning*, ser. Proceedings of Machine Learning Research, S. Dasgupta and D. McAllester , Eds., vol. 28 (PMLR, 17–19 Jun 2013), 1139–1147, https://proceedings.mlr.press/v28/sutskever13.html.

[61] Y. Bengio , J. Louradour , R. Collobert , and J. Weston , “Curriculum Learning,” in *Proceedings of the 26th Annual International Conference on Machine Learning*, ser. ICML '09 (Association for Computing Machinery, 2009): 41–48, 10.1145/1553374.1553380.

[62] S. A. Frank , “The Common Patterns of Nature,” Journal of Evolutionary Biology 22, no. 8 (08 2009): 1563–1585, 10.1111/j.1420-9101.2009.01775.x. 19538344 PMC2824446

[63] R. A. Watson , G. P. Wagner , M. Pavlicev , D. M. Weinreich , and R. Mills , “The Evolution of Phenotypic Correlations and “Developmental Memory”,” Evolution 68, no. 4 (Feb. 2014): 1124–1138, 10.1111/evo.12337. 24351058 PMC4131988

[64] R. A. Fisher , The Genetical Theory of Natural Selection (Clarendon Press, 1930), 10.5962/bhl.title.27468.

[65] J. Schmidhuber , “Reinforcement Learning Upside Down: Don't Predict Rewards – Just Map Them to Actions,” (2020), https://arxiv.org/abs/1912.02875.

[66] R. K. Srivastava , P. Shyam , F. Mutz , W. Jaśkowski , and J. Schmidhuber , “Training Agents Using Upside‐Down Reinforcement Learning,” (2021), https://arxiv.org/abs/1912.02877.

[67] H. Hazan and M. Levin , “Exploring the Behavior of Bioelectric Circuits Using Evolution Heuristic Search,” Bioelectricity 4, no. 4 (2022): 207–227, 10.1089/bioe.2022.0033.

[68] M. Levin , “Self‐Improvising Memory: A Perspective on Memories as Agential, Dynamically Reinterpreting Cognitive Glue,” Entropy 26, no. 6 (2024), https://www.mdpi.com/1099‐4300/26/6/481. 10.3390/e26060481 PMC11203334 38920491

[69] J. Lehman and K. O. Stanley , “Abandoning Objectives: Evolution Through the Search for Novelty Alone,” Evolutionary Computation 19, no. 2 (2011): 189–223. 20868264 10.1162/EVCO_a_00025

[70] J. K. Pugh , L. B. Soros , and K. O. Stanley , “Quality Diversity: A New Frontier for Evolutionary Computation,” Frontiers in Robotics and AI 3 (2016), https://www.frontiersin.org/journals/robotics‐and‐ai/articles/10.3389/frobt.2016.00040.

[71] D. Lombardi and S. Pant , “Nonparametric k‐nearest‐neighbor entropy estimator,” Physical Review E 93 (Jan. 2016): 013310, 10.1103/PhysRevE.93.013310. 26871193

[72] S. Risi , Y. Tang , D. Ha , and R. Miikkulainen , Neuroevolution: Harnessing creativity in ai agent design (MIT Press 2025), https://neuroevolutionbook.com.

[73] K. Hornik , M. Stinchcombe , and H. White , “Multilayer feedforward networks are universal approximators,” Neural networks 2, no. 5 (1989): 359–366.

[74] J. Schulman , F. Wolski , P. Dhariwal , A. Radford , and O. Klimov , “Proximal policy optimization algorithms,” (2017), https://arxiv.org/abs/1707.06347.

[75] D. Silver , S. Singh , D. Precup , and R. S. Sutton , “Reward is enough,” Artificial Intelligence 299 (Oct. 2021): 103535, 10.1016/j.artint.2021.103535.

[76] N. Bostrom , “Existential risk prevention as global priority,” Global Policy 4, no. 1 (2013): 15–31, https://onlinelibrary.wiley.com/doi/abs/10.1111/1758‐5899.12002.

[77] B. Hartl , M. Levin , and A. Zöttl , “Neuroevolution of Decentralized Decision‐Making in N‐Bead Swimmers Leads to Scalable and Robust Collective Locomotion,” Communications Physics 8, no. 1 (May 2025), 10.1038/s42005-025-02101-5.

[78] B. Hartl , M. Hübl , G. Kahl , and A. Zöttl , “Microswimmers learning chemotaxis with genetic algorithms,” Proceedings of the National Academy of Sciences 118, no. 19 (May 2021), 10.1073/pnas.2019683118. PMC8126864 33947812

[79] Y. Tang and D. Ha , “The sensory neuron as a transformer: Permutation‐invariant neural networks for reinforcement learning,” in Advances in Neural Information Processing Systems, A. Beygelzimer , Y. Dauphin , P. Liang , and J. W. Vaughan , Eds., (Curran Associates, Inc., 2021), https://openreview.net/forum?id=wtLW‐Amuds.

[80] Y. Tang , D. Nguyen , and D. Ha , “Neuroevolution of self‐interpretable agents,” in *Proceedings of the 2020 Genetic and Evolutionary Computation Conference*, ser. GECCO '20 (Association for Computing Machinery, 2020), 414–424, 10.1145/3377930.3389847.

[81] K. O. Stanley and R. Miikkulainen , “Evolving Neural Networks through Augmenting Topologies,” Evolutionary Computation 10, no. 2 (2002): 99–127, 10.1162/106365602320169811. 12180173

[82] D. E. Rumelhart , G. E. Hinton , and R. J. Williams , Learning Internal Representations by Error Propagation (MIT Press, 1986), 318–362.

[83] G. Brockman , V. Cheung , L. Pettersson , J. Schneider , J. Schulman , J. Tang , and W. Zaremba , “OpenAI Gym,” (2016), https://arxiv.org/abs/1606.01540.

[84] J. Mathews and M. Levin , “The Body Electric 2.0: Recent Advances in Developmental Bioelectricity for Regenerative and Synthetic Bioengineering,” Current Opinion in Biotechnology 52 (Aug. 2018): 134–144, 10.1016/j.copbio.2018.03.008. 29684787 PMC10464502

[85] K. O. Stanley and J. Lehman , Why Greatness Cannot Be Planned: The Myth of the Objective (Springer, 2015).

[86] A. Ahrari , S. M. Elsayed , R. A. Sarker , D. Essam , and C. A. C. Coello , “Static and Dynamic Multimodal Optimization by Improved Covariance Matrix Self‐Adaptation Evolution Strategy with Repelling Subpopulations,” IEEE Transactions on Evolutionary Computation 26, no. 3 (2022): 527–541, 10.1109/TEVC.2021.3117116. 27070282

[87] A. Ahrari , K. Deb , and M. Preuss , “Multimodal Optimization by Covariance Matrix Self‐Adaptation Evolution Strategy with Repelling Subpopulations,” Evolutionary Computation 25, no. 3 (2017): 439–471, 10.1162/evco_a_00182. 27070282

[88] J.‐B. Mouret and J. Clune , “Illuminating Search Spaces by Mapping Elites,” *ArXiv* abs/1504.04909 (2015), https://arxiv.org/abs/1504.04909.

[89] M. Levin , “Technological Approach to Mind Everywhere: An Experimentally‐Grounded Framework for Understanding Diverse Bodies and Minds,” Frontiers in Systems Neuroscience 16 (2022), https://www.frontiersin.org/journals/systems‐neuroscience/articles/10.3389/fnsys.2022.768201/full. 10.3389/fnsys.2022.768201 PMC8988303 35401131

[90] D. Vernon , R. Lowe , S. Thill , and T. Ziemke , “Embodied Cognition and Circular Causality: On the Role of Constitutive Autonomy in the Reciprocal Coupling of Perception and Action,” Frontiers in Psychology 6 (2015), https://www.frontiersin.org/journals/psychology/articles/10.3389/fpsyg.2015.01660. 10.3389/fpsyg.2015.01660 PMC4626623 26579043

[91] J. Gordon , A. Maselli , G. L. Lancia , T. Thiery , P. Cisek , and G. Pezzulo , “The Road towards Understanding Embodied Decisions,” Neuroscience & Biobehavioral Reviews 131 (2021): 722–736, https://www.sciencedirect.com/science/article/pii/S0149763421004164. 34563562 10.1016/j.neubiorev.2021.09.034 PMC7614807

[92] A. Mordvintsev , E. Randazzo , E. Niklasson , and M. Levin , “Growing Neural Cellular Automata,” Distill 5, no. 2 (Feb. 2020), 10.23915/distill.00023.

[93] X. Li and A. G.‐O. Yeh , “Neural‐Network‐Based Cellular Automata for Simulating Multiple Land Use Changes Using GIS,” International Journal of Geographical Information Science 16, no. 4 (2002): 323–343, 10.1080/13658810210137004.

[94] M. J. Ramstead , A. Constant , P. B. Badcock , and K. J. Friston , “Variational Ecology and the Physics of Sentient Systems,” Physics of Life Reviews 31 (2019): 188–205, https://www.sciencedirect.com/science/article/pii/S157106451930003X. 30655223 10.1016/j.plrev.2018.12.002 PMC6941227

[95] J. J. Hopfield , “Neural Networks and Physical Systems with Emergent Collective Computational Abilities,” Proceedings of the National Academy of Sciences 79, no. 8 (1982): 2554–2558, https://www.pnas.org/doi/abs/10.1073/pnas.79.8.2554. 10.1073/pnas.79.8.2554 PMC346238 6953413

[96] K. Friston , D. A. Friedman , A. Constant , V. B. Knight , C. Fields , T. Parr , and J. O. Campbell , “A Variational Synthesis of Evolutionary and Developmental Dynamics,” Entropy 25, no. 7 (2023), https://www.mdpi.com/1099‐4300/25/7/964. 10.3390/e25070964 PMC10378262 37509911

[97] J. Jaeger and N. Monk , “Bioattractors: Dynamical Systems Theory and the Evolution of Regulatory Processes,” The Journal of Physiology 592, no. 11 (2014): 2267–2281. 24882812 10.1113/jphysiol.2014.272385 PMC4048087

[98] U. Alon , *An Introduction to Systems Biology: Design Principles of Biological Circuits*, 0th ed (Chapman and Hall/CRC), https://www.taylorfrancis.com/books/9781420011432.

[99] U. Alon , “Network Motifs: Theory and Experimental Approaches,” Nature Reviews Genetics 8, no. 6 (June 2007): 450–461. 10.1038/nrg2102 17510665

[100] E. H. Davidson and D. H. Erwin , “Gene Regulatory Networks and the Evolution of Animal Body Plans,” Science 311, no. 5762 (2006): 796–800. 16469913 10.1126/science.1113832

[101] M. Levine and E. H. Davidson , “Gene Regulatory Networks for Development,” Proceedings of the National Academy of Sciences 102, no. 14 (2005): 4936–4942, https://pnas.org/doi/full/10.1073/pnas.0408031102. 10.1073/pnas.0408031102 PMC555974 15788537

[102] S. A. Kauffman , “Metabolic Stability and Epigenesis in Randomly Constructed Genetic Nets,” Journal of Theoretical Biology 22, no. 3 (1969): 437–467, https://www.sciencedirect.com/science/article/pii/0022519369900150. 5803332 10.1016/0022-5193(69)90015-0

