# Supplementary material for: Heuristically Adaptive Diffusion‐Model Evolutionary Strategy
Source: Adv Sci (Weinh). 2026 Mar 7;13(20):e11537. doi: 10.1002/advs.202511537 (PMC13067789; doi:10.1002/advs.202511537)
Supplement: Supplementary file 1 — Supporting File 1: advs72244‐sup‐0001‐SuppMat.pdf. [file ADVS-13-e11537-s002.pdf]

Supporting Information for

Heuristically Adaptive Diffusion-Model Evolutionary Strategy

S1 Related Works

Recent approaches have begun bridging the conceptual gaps between evolutionary- and developmental biology, and technological applications. One promising direction employs Variational Auto-Encoders (VAEs) (*1*) to create low-dimensional genotypic search spaces for evolutionary algorithms, while conducting fitness evaluations in higher dimensional parameter spaces (*2, 3*). A related approach, termed Deep Optimisation (*4*), leverages multi-level evolutionary transitions, modeled with deep learning, to solve complex combinatorial problems with polynomial scaling. However, these methods tend to exhibit greedy behavior and require careful curation of the training dataset and architecture to enable exploration in a successively refined latent space representation rather than being constrained by the decoder's canalizing output (*4*). While they effectively compress information from the training data to enable generalization, they do so by prioritizing a low bias–variance trade-off that minimizes variance on training error, ultimately limiting their capacity to generalize beyond the training data. Significant questions remain about how biological evolution generates novelty (*5, 6, 7, 8, 9, 10, 11, 12, 13, 14, 15*). Computational techniques such as novelty search (*16*) and quality-diversity algorithms (*17*) have made progress in addressing these questions, though gaps in our understanding persist. Recent efforts have started drawing connections between evolutionary processes and broader concepts of intelligence (*18, 19, 5, 20*) and learning theory (*21, 22, 7, 23, 24*).

Neural Cellular Automata (NCAs) (*25, 26, 27, 28*), computational tools from the field of *Artificial Life* (*29*), serve as generative models for studying morphogenesis *in silico* through collective multi-agent processes. NCAs are particularly valuable for investigating the indirect encoding relationship between genotype and phenotype, as they capture the emergent, multi-scale properties inherent in biological development. Our recent work (*25*) demonstrates that hierarchical functional genotypic encoding—a characteristic found in biological systems and modeled by NCAs—fundamentally influences evolutionary processes and enables rapid, modular adaptation to environmental changes (*30, 31, 32, 14, 15*). The multi-scale competency architecture of NCAs

manifests through local cell-cell interactions generating global patterns and behaviors. This architecture enables collective pattern formation through distributed computation, while maintaining robust development despite perturbations. Furthermore, it facilitates adaptive responses across multiple spatial and temporal scales, culminating in the self-organization of modular, hierarchical structures. These characteristics mirror biological development's collective (intercellular) intelligence. However, training NCAs through gradient or evolutionary methods remains challenging. Related promising approaches such as neuro-evolution techniques (*33*), growing and self-assembling artificial neural networks (*34*), neural developmental programming (*35*), and self-modeling approaches (*36, 37, 38*) have further expanded our understanding. These methodologies collectively enhance our comprehension of how indirect encoding facilitates the emergence of complex, adaptive behaviors from simple, local rules.

S2 Methods

S2.1 Evolutionary algorithms: Black-box heuristic optimization techniques

The principles of evolution have applications beyond biology, proving useful in addressing complex systems across different domains. The main components of this process - imperfect replication with heredity and fitness-based selection - are versatile and can be applied in diverse fields. In computer and data science, numerous optimization methods are employed; among the most widely used is stochastic gradient descent (SGD), which, with advancements like the Adam optimizer, excels in tasks where gradient calculations provide clear direction toward solutions. However, not all tasks crated equal, some are amenable to gradient-based methods, as gradient calculation can be intractable for many complex problems. For these cases, Evolutionary Algorithms (EAs) such as CMA-ES (*39*) and PEPG (*40*) are essential. These heuristic optimization techniques (*41, 42, 43, 44*) maintain and evolve a population of genotypic parameters over successive generations using biologically inspired operations, including selection, reproduction, crossover, and mutation. The goal is to gradually adapt the genotypic parameters of the entire population so individual phenotypic samples perform well when evaluated against an objective- or fitness function. The evaluated numerical fitness score of an individual correlates with its probability of survival and reproduction to drive

the evolutionary process toward more optimal solutions. Thus, these algorithms use evolutionary biology dynamics to discover optimal or near-optimal solutions within vast, complex, and otherwise intractable parameter spaces. Such approaches are particularly valuable when heuristic solutions are needed to explore extensive combinatorial and permutation landscapes.

EAs can work with either discrete or continuous sets of parameters, with the former being a subset of the latter. Our focus here is on continuous parameter spaces that have domain-specific structures, which are typically *a priori* unknown. As a result, the initial population is often sampled from a standard normal distribution. This population is then progressively refined with each generation to excel on a specific objective function. Essentially, the initially random parameters are heuristically adjusted by evolutionary algorithms, gradually transforming into highly structured parameters that perform effectively on the given task, with the goal of optimizing the objective function to solve the problem at hand.

The reproduction process of EAs to generate novel offspring parameters can either be population based through recombination and mutation operations at the genotypic level, or even leveraged by sampling novel data-points from successively re-parameterized probabilistic models, *e.g.*, with a Gaussian prior (*39*). In essence, evolutionary processes thus act like generative models that are parameterized, or trained, based on heuristic information gathered from previously explored areas of the parameter space, at least from the prior generation and considering the current state of their underlying generative model. This setup is aimed at generating offspring that may be better adapted for the next generation. Furthermore, as we demonstrate in a complementary contribution 45, these evolutionary processes have similarities to diffusion models

S2.2 Integrating Diffusion Models as Offspring-Generative Process in Evolutionary Algorithms

The evolutionary process can be viewed as a transformation of genotype or phenotype distributions – or, more generally, parameter distributions. The current population's distribution undergoes selection and mutation, evolving into a slightly different distribution after each time step. This perspective highlights the great potential of using generative models to simulate evolutionary dynamics. In particular, diffusion models, which have achieved state-of-the-art performance across

various generative tasks–including image, video, and audio generation–are well-suited for modeling the complex distributional changes inherent in evolution. Given their capability to capture intricate data distributions, we hypothesize that a well-trained diffusion model should outperform traditional methods in evolutionary strategies.

Therefore, we propose diffusion model-based evolutionary strategies to generate offspring efficiently by sampling more from high-fitness regions in the parameter space. Given a population $\{\boldsymbol{g}_{\tau,i}\}$ at time τ, we evaluate their fitness values $f_{\tau,i} = f(\boldsymbol{g}_{\tau,i})$ using a fitness function $f : \mathbb{R}^n \longrightarrow \mathbb{R}$. Our key idea is to train a generative model on the current population and their associated fitness values, then sample the next generation $\{\boldsymbol{g}_{\tau,i}\}$, which contains more high-fitness individuals. To achieve this, we map the samples $\{\boldsymbol{g}_{\tau,i}\}$ and their fitness values $f_{\tau,i}$ to a density distribution and train a diffusion model on it. Specifically, we use a function $h : \mathbb{R} \longrightarrow \mathbb{R}^+$ to map fitness to a fitness-derived probability density, such that $p(\boldsymbol{g}) \propto h[f(\boldsymbol{g})]$.

Diffusion models consist of two phases: a forward diffusion phase and a reverse denoising phase. In the forward diffusion phase, noise is gradually blended into the training data–a process known as diffusion. A neural network is then trained to predict the added noise given the noisy data. In the reverse phase, starting from noisy data, the trained neural network is used to denoise step by step, eventually restoring the noise-free data.

Formally, during the forward diffusion phase, the noise-free data sampled from $\boldsymbol{g}$ is considered at time zero, i.e., x_0. It is blended with noise over time according to:

$$\boldsymbol{x}_t = \sqrt{\alpha_t}\,\boldsymbol{x}_0 + \sqrt{1-\alpha_t}\,\boldsymbol{\epsilon}, \tag{S1}$$

where $\boldsymbol{\epsilon} \sim \mathcal{N}(0, I^D)$, and α_t decreases monotonically from 1 to 0 as t increases, with $\alpha_0 = 1$ and $\alpha_T = 0$. As a result, $\boldsymbol{x}_T \sim \mathcal{N}(0, I^D)$ and $\boldsymbol{x}_0 \sim \boldsymbol{g}$. More explicitly, we henceforth use the symbols $\boldsymbol{g}$ and $\boldsymbol{x}_t$ to formally distinguish between genotypic parameters in the evolutionary process and parameters subjected to the diffusion model, respectively. Thus, we consider noise-free (or denoised) data points $\boldsymbol{x}_0$ as genotypic parameters $\boldsymbol{x}_0 \sim \boldsymbol{g}$.

To denoise, a neural network ϵ_θ is trained to predict the added noise by minimizing the prediction loss L:

$$\theta = \arg\min_\theta L(\theta) = \arg\min_\theta \sum_{t=1}^{T} \sum_{\boldsymbol{x}\in\mathbb{R}^n} p(\boldsymbol{x}) \left\| \epsilon_\theta\left(\sqrt{\alpha_t}\,\boldsymbol{x} + \sqrt{1-\alpha_t}\,\boldsymbol{\epsilon}, t\right) - \boldsymbol{\epsilon} \right\|^2. \tag{S2}$$

Given the difficulty in obtaining the exact probability density $p(\boldsymbol{x})$ for the training data, diffusion models are trained by sampling data points $\boldsymbol{x} \sim \boldsymbol{g}$ and time steps $t \in [1, T]$. Therefore, the loss function can be reformulated without explicitly using $p(\boldsymbol{x})$:

$$L(\theta) = \mathbb{E}_{t \sim \mathcal{U}(0,T), \boldsymbol{x} \sim \boldsymbol{g}} \left\| \epsilon_\theta \left(\sqrt{\alpha_t}\, \boldsymbol{x} + \sqrt{1 - \alpha_t}\, \boldsymbol{\epsilon}, t \right) - \boldsymbol{\epsilon} \right\|^2 . \tag{S3}$$

After training the model, the neural network ϵ_θ can be used to sample new data points that follow the distribution of the training data. In the Diffusion Denoising Implicit Model (DDIM) (*46*) framework, the sampling process is an iterative refinement:

$$\boldsymbol{x}_{t-1} = \sqrt{\alpha_{t-1}} \left(\frac{\boldsymbol{x}_t - \sqrt{1 - \alpha_t}\, \boldsymbol{\epsilon}_\theta(\boldsymbol{x}_t, t)}{\sqrt{\alpha_t}} \right) + \sqrt{1 - \alpha_{t-1} - \sigma_t^2} \cdot \epsilon_\theta(\boldsymbol{x}_t, t) + \sigma_t\, \boldsymbol{\epsilon}_t, \tag{S4}$$

where $\boldsymbol{\epsilon}_t \sim \mathcal{N}(0, I^D)$, and σ_t is the noise amount. By default, we use $\sigma_t = \sqrt{\frac{1-\alpha_{t-1}}{1-\alpha_t}} \sqrt{1 - \frac{\alpha_t}{\alpha_{t-1}}}$. Starting with $\boldsymbol{x}_T \sim \mathcal{N}(0, I^D)$, this iterative process generates $\boldsymbol{x}_0 \sim \boldsymbol{g}$.

To apply this to evolutionary tasks, we aim to assign higher probabilities to high-fitness individuals during sampling. Inspired by Equation (S2), we introduce a weighting function $h[f(\boldsymbol{x})]$ into the loss function to bias the model towards high-fitness samples:

$$L_{\text{evo}}(\theta) = \mathbb{E}_{t \sim \mathcal{U}(0,T), \boldsymbol{x} \sim \boldsymbol{g}}\, h[f(\boldsymbol{x})] \left\| \epsilon_\theta \left(\sqrt{\alpha_t}\, \boldsymbol{x} + \sqrt{1 - \alpha_t}\, \boldsymbol{\epsilon}, t \right) - \boldsymbol{\epsilon} \right\|^2 . \tag{S5}$$

With this modified loss function, the sampled data x_0 will follow a combined distribution of the original data and the fitness-derived distribution, i.e., $\boldsymbol{x}_0 \sim h[f(\boldsymbol{x}_0)]\, p_{\boldsymbol{g}}(\boldsymbol{x}_0)$, where $p_{\boldsymbol{g}}$ represents the sample distribution.

We encapsulate this process into a function $\mathcal{G}$, which takes the current population $\boldsymbol{g}_\tau$ and their fitness-derived density $p(\boldsymbol{g}_\tau)$ to produce the new population $\boldsymbol{g}_{\tau+1}$:

$$\mathcal{G} : (\boldsymbol{g}_\tau,\ p(\boldsymbol{g}_\tau)) \rightarrow \boldsymbol{g}_{\tau+1}. \tag{S6}$$

This mirrors the evolutionary process: at each step, we bias the sampling distribution $p_{\boldsymbol{g}}$ with the fitness-derived probability density, effectively sampling more from high-fitness regions (selection). The inherent randomness in sampling introduces variation (mutation).

Different from our previous approach, by framing offspring generation as a generative process, we gain more control over this process. An important application is the use of conditional generation to steer evolution, including controlling population features, maintaining diversity, and even directly influencing fitness.

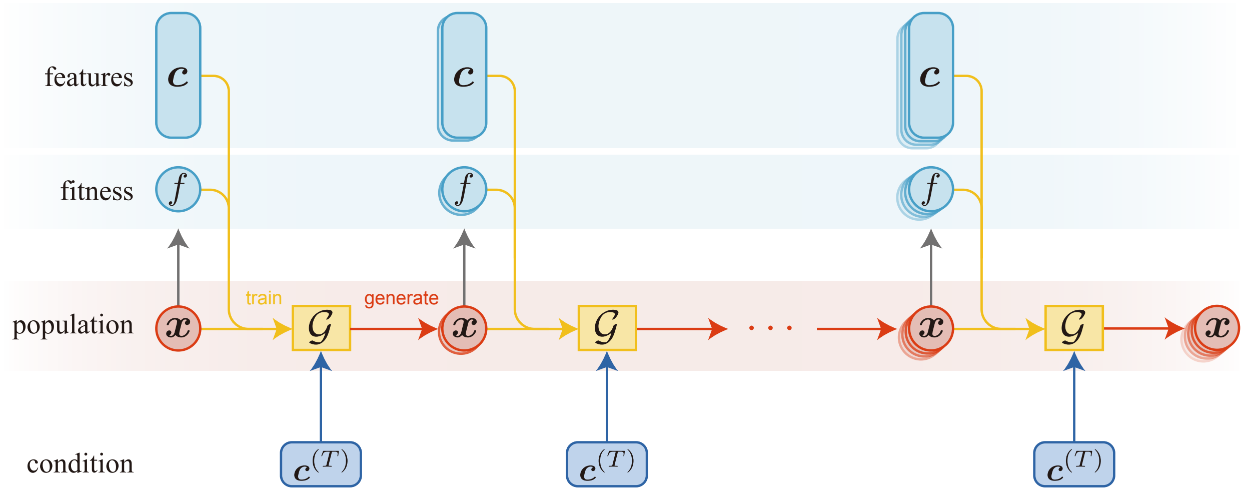

Figure S1: Workflow of the *CHARLES-D* algorithm. Starting with a randomly initialized population (red circles), their fitness and features (shown in rounded blue rectangles) are evaluated. Next, a generative model $\mathcal{G}$ (yellow rectangles) is trained on this population, weighted by their fitness, with features used as conditioning for generation. Following training, external conditions are provided to generate a new population that meets specified requirements. The evaluation-training-generation loop is then repeated. A buffer is maintained to store the population along with their fitness and features for training, enabling full data utilization and preserving population diversity.

S2.3 Conditional, Heuristically-Adaptive ReguLarized Evolutionary Strategy through Diffusion (*CHARLES-D*)

DMs provide a model-free approach for learning denoising-based generative strategies tailored to custom datasets across versatile, problem-specific domains. Once trained on statistically relevant data, they can potentially surpass traditional EAs in generating high-quality offspring genotypes. In our complementary contribution (*45*), we formally connect DMs to EAs and particularly demonstrate that the backward process in DMs can be viewed as an iterative evolutionary process across generations.

Here, we introduce a paradigm shift by sustaining and evolving a heuristic population $\boldsymbol{G}_\tau = \{\boldsymbol{g}_{\tau,1}, \boldsymbol{g}_{\tau,2}, \ldots, \boldsymbol{g}_{\tau,N_p}\} \sim p_{\boldsymbol{X}_{\tau-1}}$ that is sampled across successive generations $\tau = 1, 2, \ldots, N_\tau$ from $p_{\boldsymbol{X}_{\tau-1}}$ via a heuristically refined diffusion model $\mathcal{G}_{\tau-1} \to \mathcal{G}_\tau$ (see SI Section S2.2). This model is constantly refined - *i.e.*, trained "online" - on a successively acquired dataset buffer containing elite solutions of previous generations $\boldsymbol{X}_{\tau-1} = \{\boldsymbol{G}_{\tau'<\tau}\}$ that have been sampled by prior versions of the DM. In training the DM, we notably weight high-fitness data more heavily compared to

low-fitness genotypes using a fitness weighting function $h[f(\boldsymbol{g})]$. This approach increases the probability of sampling high-quality data while still maintaining diversity in the generative process (see Sections S2.2 and S3.1 for details). We then use $\mathcal{G}_{\tau+1}$ as a generative model for sampling high-quality genotypic parameters $\boldsymbol{G}_{\tau+1} \sim p_{X_\tau}$ of the next generation $\tau + 1$, with successively larger fitness than the prior generations. We thus propose a "Heuristically Adaptive Diffusion-Model Evolutionary Strategy" (*HADES*) for learning good and model-free reproductive strategies in EAs (compared to, *e.g.*, the Gaussian prior in CMA-ES) by training DMs on fitness-weighted datasets of genotypic data; see pseudo-code in algorithm 1 and illustrations in fig. 1 and SI Figure S1.

This approach allows us to adaptively generate novel offspring parameters by refining either randomly initialized "proto"-genotypes $\boldsymbol{x}_T \sim \mathcal{N}(0, {\sigma_I}^D)$ (default), or recombined and mutated genetic material from elite solutions i, j of the prior generation τ, schematically expressed as $\boldsymbol{g}_{\tau,i} \bigoplus \boldsymbol{g}_{\tau,j} + \boldsymbol{\epsilon}_t$ (see SI Section S3.2 for details). Notably, the latter option of initiating the generative process of the DM with recombined genetic information relates to inpainting techniques (*47*), which aim to complete missing information in sample data. In traditional inpainting applications, missing information in masked or patched images is integrated seamlessly into the scene. Similarly, in our context, potentially conflicting parameter combinations from the genetic crossover operation (with potential effects on corresponding fitness scores) can be resolved by the DM, functioning in a manner akin to error-correction mechanisms (*8*).

Intriguingly, with DMs, we can apply techniques such as classifier-free guidance (*48*) to condition the generation process. This allows us to implement an evolutionary optimizer whose search dynamics can be controlled without relying on additional reward-shaping techniques (*49*). By training the DM with additional information $\boldsymbol{c}_i = c(\boldsymbol{g}_i)$, which numerically quantifies certain qualities or traits of genotypes $\boldsymbol{g}_i$ in their respective environments, the DM learns to associate (*50*) elements in the parameter space with corresponding classifiers $\boldsymbol{g}_i \leftrightarrow \boldsymbol{c}_i$.

Technically, this is achieved by extending the input of the DM's ANN as $\epsilon_\theta(\boldsymbol{x}_t, t) \longrightarrow \epsilon_\theta(\boldsymbol{x}_t, t, c(\boldsymbol{x}_t))$. The function $c(\cdot)$ is a custom, not necessarily differentiable, vector-valued classifier function or a measurement of a trait of the data point x, or genotype $\boldsymbol{g}$, evaluated in the parameter space, fitness space, or even phenotype space.

During sampling, the DM's generative process can be biased towards novel high-quality data points that exhibit a particular target trait $\boldsymbol{c}^{(\mathrm{T})}$, by conditioning the iterative denoising process of

Algorithm 1 Pseudo-code of *HADES* (*CHARLES-D*): novel generations are consecutively sampled by a heuristically refined DM. The seeds for this sampling are yet again sampled from crossover and mutation operations of the current population and from Gaussian noise to allow both adaptation of (combinations of) elite solutions and unbiased sampling by the DM. *CHARLES-D* can be conditionally biased during sampling. Notably, replacing the DM, $\mathcal{G}$, with a multivariate Gaussian model, and refraining from the option of using conditional sampling, would essentially recover the CMA-ES (*39*) algorithm.

Require: Population size N_p, parameter dimension D, initial STD σ_I, fitness function f, weighting function h, diffusion model $\mathcal{G}$, crossover ratio N_c, total evolution steps N_τ, classifier function c, target condition $\boldsymbol{c}^{(T)}$.

Ensure: $N_c < N_p$

$\boldsymbol{G}_1 \leftarrow \mathcal{N}(0, {\sigma_I}^{N_p \times D})$ ▷ Initialize population

$\mathbf{X} \leftarrow \{\varnothing\}$ ▷ Initialize dataset buffer

for $\tau \in [1, 2, ..., N_\tau]$ **do**

  $\{\boldsymbol{g}_1, \boldsymbol{g}_2, ..., \boldsymbol{g}_{N_p}\} \leftarrow \boldsymbol{G}_\tau$

  $\forall i \in [1, N_p] : q_i \leftarrow h[f(\boldsymbol{g}_i; \tau)]$

  $\forall i \in [1, N_p] : \boldsymbol{c}_i \leftarrow c(\boldsymbol{g}_i; \tau)$

  $\mathbf{X} \leftarrow \mathbf{X} \bigoplus \{(\boldsymbol{g}_i, q_i, \boldsymbol{c}_i)\}$ ▷ Cache associated (data-point, fitness weight, and classifier)-tuples

  $\mathcal{G}_\tau \leftarrow \text{train}(\mathcal{G}, \mathbf{X})$ ▷ (Re)train diffusion model on updated dataset buffer

  $\tilde{\boldsymbol{G}}_{\tau+1} \leftarrow \text{crossover}(\boldsymbol{G}_\tau | q_i, q_j)^{N_c} \bigoplus N(0, {\sigma_I}^{(N_p - N_c) \times D})$ ▷ Sample "proto"-genomes via crossover and noisy data

  $\boldsymbol{G}_{\tau+1} \leftarrow \mathcal{G}_\tau : p_{\mathbf{X}}(\tilde{\boldsymbol{G}}_{\tau+1} | \boldsymbol{c}^{(T)})$ ▷ Sample next generation conditional to target traits via refined denoising

end for

the diffusion model as $\epsilon_\theta(\boldsymbol{x}_t, t, \boldsymbol{c}^{(\mathrm{T})}) \rightarrow \hat{\boldsymbol{x}}_0$ such that $c(\hat{\boldsymbol{x}}_0) \approx \boldsymbol{c}^{(\mathrm{T})}$. This allows the DM to generate high-quality samples with the desired traits, similar to how Stable Diffusion (*51*) and Sora (*52*) generate realistic image or video content based on custom text prompts.

In our context, we propose using conditional sampling to gain exceptional control over a heuristic search process with an open, successively refining dataset. While the heuristic nature of the evolutionary process facilitates global optimum exploration, the successively refined DM-based generative process allows for diverse sampling of high-quality genotypic data points that may exhibit target traits defined independently from the fitness score, akin to prompting an image-generative DM with text input.

We consider this approach a "Talk to your Optimizer" application and refer to this method as *Conditional, Heuristically-Adaptive Regularized Evolutionary Strategy through Diffusion* (*CHARLES-D*). The pseudocode and a visualization are provided in algorithm 1 and SI Figure S1, where deviations of *CHARLES-D* from the *HADES* method in the training and sampling steps are color-coded for clarity.

S3 Algorithmic Details

S3.1 Fitness re-weighting with the Roulette-Wheel Method

In order to reweigh the importance of parameters $\boldsymbol{g}_i$ from the training dataset $\boldsymbol{X} = \{\boldsymbol{g}_1, \boldsymbol{g}_2, \ldots, \boldsymbol{g}_i, \ldots, \boldsymbol{g}_{N_p}\}$ during training of the diffusion models, we rely on a reweighing function $h[f_i]$ of the parameter fitness $f_i = f(\boldsymbol{g}_i)$. Inspired by "roulette-wheel selection" (*44*), we define the remapping function $h[f_i]$ assuming sorted fitness scores, $f_i \leq f_{i+1}$, as

$$h[f_i; s] = \frac{\sum_{j=1}^{i} F(f_j; s)}{\sum_{k=1}^{N_p} \sum_{j=1}^{k} F(f_j; s)}, \tag{S7}$$

where we introduced the relative fitness $F(f_i; s) = \exp\left(s(f_i - f_{\min})/(f_i - f_{\max})\right)$, with selection pressure s. $f_{\min}$ and $f_{\max}$ are the minimum and maximum fitness values associated to the elements in current population $\boldsymbol{G}_\tau = \{\boldsymbol{g}_{\tau,i}\}$.

S3.2 Blending Generative- and Heuristic Crossover with Mutations

To generate a novel generation $\boldsymbol{G}_{\tau+1} = \{\boldsymbol{g}_{\tau+1,i}\}$, we either rely on the **generative process** $\boldsymbol{g}_{\tau+1,j} \sim p(x)$ of the diffusion model $\mathcal{G}$, or perform **manual crossover** operations $\boldsymbol{g}_{\tau+1,k} = \boldsymbol{g}_{\tau,l} \bigoplus \boldsymbol{g}_{\tau,m}$ of existing genetic material in the current population $\boldsymbol{G}_\tau$.

We sample a total number of $(N_p - N_c)$ novel parameters from the diffusion model, and perform N_c manual crossover operations; thus, the parameter $N_c \in [0, N_p]$ is termed *crossover ratio*.

Additionally, we employ **mutation operations** manually for both parameters sampled by the diffusion model and from manual crossover. To this end, we define a *mutation scale* parameter t_μ, and fully rely on the diffusion operation defined in SI Equation (S1) to mutate a parameter $\boldsymbol{g} \to \sqrt{\alpha_{t_\mu}}\boldsymbol{g} + \sqrt{1 - \alpha_{t_\mu}}\boldsymbol{\epsilon}$, with $\boldsymbol{\epsilon} \sim \mathcal{N}(0, I^D)$. Moreover, we define a *mutation ratio* $N_\mu \in [0, N_p]$ specifying the fraction of the novel generation $\boldsymbol{G}_{\tau+1}$ that is randomly selected to be subjected to mutation via t_μ diffusion steps.

We allow "readaption" of such noisy parameters at a *readaptation rate* t_a: to this end, we use the novel and mutated population as initial configurations for the diffusion model and perform t_a denoising steps, *i.e.*, starting at diffusion-time $t = t_a$; notably, $\boldsymbol{x}_{t=0}$ represent fully denoised samples while $\boldsymbol{x}_{t=T}$ samples are fully diffused.

S3.3 Symbols

All symbols used in our methods are collected in SI Table S1.

Table S1: List of Symbols and Notations

Symbol	Definition	Example/Usage
General Parameters		
D	Parameter dimension	Dimension of search space
τ	Generation index	$\tau = 1, 2, \ldots, N_\tau$

Continued on next page

Table S1 – continued from previous page

Symbol	Definition	Example/Usage
N_τ	Total number of generations	Evolution stops at $\tau = N_\tau$
N_p	Population size	Number of individuals per generation
$N_\mathcal{B}$	Buffer size	Number of individuals in the dataset buffer
N_e	Elite ratio	Number of best performing individuals from population
N_c	Crossover ratio	Number of individuals from population for crossover
N_μ	Mutation ratio	Number of individuals from population for mutation
t_μ	Mutation scale	Number of diffusion time-steps used to mutate samples
t_a	Readaptation rate	Number of diffusion time-steps to readapt (de mutated samples
σ_I	Initial standard deviation	STD of initial population
s	Selection pressure	Sharpness of “roulette wheel selection“ fitness reweighing, see SI Section S3.1
Population and Genome		
$\boldsymbol{g}$	Genome variables	Individual parameter set
$\boldsymbol{G}$	Population	Set of all genomes in a generation
$\boldsymbol{G}_\tau$	Population at generation τ	$\{\boldsymbol{G}_{\tau,1}, \boldsymbol{g}_{\tau,2}, \ldots, \boldsymbol{g}_{\tau,N_p}\}$

Continued on next page

Table S1 – continued from previous page

Symbol	Definition	Example/Usage
$\boldsymbol{X}$	Dataset buffer	Collection of past elite solutions
Diffusion Model Parameters		
t	Diffusion time	$t = 1, 2, \ldots, T$
$\boldsymbol{x}_t$	State at diffusion time t	Parameters during diffusion process
α_t	Noise schedule	Controls noise level at time t
$\boldsymbol{\epsilon}$	Random noise	$\boldsymbol{\epsilon} \sim \mathcal{N}(0, I^D)$
ϵ_θ	Neural network	Predicts noise during denoising
$\mathcal{G}$	Generative model	Maps current to next generation
Fitness and Conditioning		
$f(\boldsymbol{g})$	Fitness function	Evaluates quality of genome
$h[f]$	Weighting function	Maps fitness to sampling probability
w_f	Fitness-weighted training	Uses $q = h[f]$ to weight samples in the DM loss
$w_\mathcal{N}$	Fitness-selected training	Uses $h[f]$ as probability to sample from $\boldsymbol{X}$ and generate a high-fitness-biased DM training dataset where all elements are weighted equally in the DM loss
$\boldsymbol{c}$	Condition variables	Target for conditioning
$c(\boldsymbol{g})$	Classifier function	Maps genome to condition
Diffusion Model Architecture (typically feed-forward)		

Continued on next page

Table S1 – continued from previous page

Symbol	Definition	Example/Usage
$N_{\mathcal{L}}$	Number of hidden layers	-
$N_{\mathcal{H}}$	Number of hidden units per hidden layers	-
$f_{\mathcal{F}}$	Activation function of hidden units	-
λ_{LR}	Learning Rate	The learning rate used during training.
$N_{\mathcal{E}}$	Epochs	The number of training epochs.
Special Functions		
$p(\boldsymbol{x})$	Probability density	Distribution of parameters
$L(\theta)$	Loss function	Training objective for diffusion model

S4 Simulation Details

SI Table S2 provides an overview of the simulation parameters for the results depicted in the main text. Additional remarks can be found in the text below. Symbols are defined in SI Section S3.3.

Table S2: *HADES* and *CHARLES-D* simulation parameters for the results presented in the main text. See SI Table S1 for more details about the symbols.

Experiment	Solver	N_p	σ_I	$N_{\mathcal{B}}/N_p$	N_e/N_p	N_c/N_p	N_μ/N_p	t_μ/T
Figure 2	*HADES*	256	0.5	1	0.15	0	1	5×10^{-2}
Figure 3	*CHARLES-D*	256	2	3	0.15	2^{-3}	1	5×10^{-2}
Figure 4	*CHARLES-D*	256	0.5	2	0	0	0.1	5×10^{-2}
Figure 5	*CHARLES-D*	256	2	5	0	2^{-3}	1	5×10^{-2}

Figure 6	*CHARLES-D*	256	0.2	10	0.25	0	0.1	2×10^{-1}
Figure 7 (B)	*HADES*	256	0.5	4	0.2	0.4	1	0.1
Figure 7 (C)	*HADES*	256	0.5	4	0.2	0.4	1	0.1
Figure 7 (D)	*CHARLES-D*	32	0.5	16	0.25	0.45	0.1	0.2
Figure 8	*CHARLES-D*	128	1.0	5	0.1	0	0.25	5×10^{-2}

**Experiment**	t_a	s	w	$N_{\mathcal{L}}$	$N_{\mathcal{H}}$	$f_{\mathcal{F}}$	λ_{LR}	$N_{\mathcal{E}}$
Figure 2	0	10	$w_{\mathcal{N}}$	3	24	Leaky-ReLU	10^{-3}	100
Figure 3	0	5	$w_{\mathcal{N}}$	3	24	Leaky-ReLU	10^{-2}	200
Figure 4	0.1	10	$w_{\mathcal{N}}$	4	32	SiLU	3×10^{-3}	100
Figure 5	0	5	$w_{\mathcal{N}}$	3	24	Leaky-ReLU	10^{-2}	200
Figure 6	0	12	w_f	2	64	ReLU	10^{-2}	200
Figure 7 (B)	0	18	$w_{\mathcal{N}}$	4	32	Leaky-ReLU	10^{-2}	200
Figure 7 (C)	0	18	$w_{\mathcal{N}}$	4	32	Leaky-ReLU	10^{-2}	200
Figure 7 (D)	0	8	w_f	3	324	ELU	10^{-3}	500
Figure 8	0	8	w_f	6	96	ELU	3×10^{-3}	500

For training the diffusion model for *HADES* and *CHARLES-D* experiments, we use the *Adam* optimizer, a batch-size of 256, and an $L2$ weight decay of $\lambda_{L2} = 10^{-5}$ in all our simulations.

For the results depicted in fig. 6, we maintain a population-size of $N_p = 256$, $k = 128$, $\beta = 10$, and $\Delta = 10^{-8}$.

In all simulations showing fitness versus generation statistics, all involved solvers maintain a fixed-size population of N_p candidate solutions across generations. This ensure a fixed fitness evaluation budget at a given generation. No simulation uses elitism (no protection of high-fitness individuals), but we use an elite ratio for evaluating fitness-weights via the roulette wheel selection, and selecting samples for crossover operations (if $N_c > 0$).

S5 Sampling Diversity Conditions

During sampling of the diffusion model in fig. 6, we need to identify target conditions $\boldsymbol{c}^{(NT)}$ that bias the generative process to bring forth novel data points $\boldsymbol{g}_\nu$ with large diversity measure. These conditions are related to entropy measures (*53*) of the current population and don't have a well-defined objective target value (the diversity measure is a dynamic property of an individual in a given population rather than an objective trait at the individual level). Thus, we sample the target conditions $\boldsymbol{c}^{(NT)} \sim p_N$ at every generation from a heuristic Boltzmann distribution $p_N \propto \exp\left(-\beta E(f, \boldsymbol{c}^{(N)})\right)$, with $E(f_i, \boldsymbol{c}^{(N)}) = \frac{\tilde{f}_i}{\boldsymbol{c}_i^{(N)} + \delta_0}$, where we introduced the rescaled fitness $\tilde{f}_i = 1 - \frac{f_i - f_{\min}}{f_{\max} - f_{\min}}$, and transform $\boldsymbol{c}_i^{(N)}$ to positive values by adding $\delta_0 = |\min_i(\delta_i) + \Delta|$. In that way, target conditions with large δ_i are sampled with higher probability, which effectively biases the generative process of the diffusion model towards sampling novel offspring genotypes with larger diversity $\delta_\nu \leq \max_i(\delta_i)$ than present in the current population, δ_i.

S6 Two-Dimensional Fitness Landscapes

S6.1 Fitness- and Diversity Conditioning for the Double-Peak Task

We used the double-peak problem as minimal toy-example for the results shown in the main text. We refer to eq. (1) for the definition and the main text for details.

Similar to fig. 6, we here discuss the results of different *HADES* and *CHARLES-D* configurations on the minimal double-peak task. The results are shown in SI Figure S2. While for this particular situation, CMA-ES and the SimpleGA converge the fastest (see averaged fitness dynamics in SI Figure S2 (B)), the Novelty-conditional *CHARLES-D* instances are second in performance, even better than the fitness-conditional and the baseline algorithms. This is rooted in the fact that the fitness signal of the narrow initial population is small, and all *HADES* and *CHARLES-D* instances are used in a non-greedy setting with low selection pressure (see SI Section S4).

Moreover, we see that the vast majority of the Novelty-conditional *CHARLES-D* instances (but also to some extend the baseline *HADES* solver) reliable identify and even stabilize on both peaks in the fitness landscape, see SI Figure S2 (D), while the more greedy fitness-conditional algorithms often collapse onto one solution after many generations. In contrast, the state-of-the-art CMA-ES

and SimpleGA methods basically zoom in onto one peak very quickly.

Notably, in contrast to the results discussed in fig. 6, we don't use any form of mutational exploration in this setting.

S6.2 Genotypic Condition for Two-peaks Fitness Function

In addition to the analysis presented in fig. 4, we extend our study to conditional generation using the two-peaks fitness function. We evaluate three distinct conditions: guiding the evolution to find solutions where $\boldsymbol{g}_i > 0$ (quadrant 1, or "target 1"), guiding evolution to find solutions where $\boldsymbol{g}_i < 0$ (quadrant 3, or "target -1"), and a control experiment where the search is conditioned on quadrants 2 and 4 with no solutions ("target 0"), c.f., fig. 3.

The results align with our expectations. As shown in SI Figures S3 and S4, starting from an initial population with STD $\sigma_I = 0.1$, the first two conditions, target ± 1, successfully steer the evolutionary process to the respective target solutions. In contrast, the control experiment, target 0, fails to find any solutions (SI Figure S5), demonstrating that an invalid condition can prevent convergence and lead to frustration effects between conditioning and fitness maximization. These findings collectively show that conditioning effectively guides the evolutionary path.

S6.3 The inverse and twisted Rastrigin Task

The Rastrigin function is defined by $f(\mathbf{x}) = An + \sum_{i=1}^{2}[x_i^2 - A\cos(2\pi x_i)]$ with $\mathbf{x} = (x_1, x_2)$; we chose $A = 10$. For the "twisted"-Rastrigin function, we use a spiral coordinate transformation $\mathbf{x} \rightarrow \tilde{\mathbf{x}} = r \times (\cos(\phi + \omega r), \sin(\phi + \omega r))$, with polar coordinates $r = |\mathbf{x}|$ and $\phi = \text{atan2}(x_2, x_1)$, and constant ω. We evaluate $h(\mathbf{x}) = f(\tilde{\mathbf{x}})$. For both the Rastrigin and twisted-Rastrigin function, we evaluate use the negative function value for our optimization experiments truncate the function values for $|x_i| > 4$ to 0. In that way, we establish an oscillatory fitness landscape with four maxima located at $|x_i^*| \approx 3.5$ for the Rastrigin function, with a maximum fitness value of $f_{\text{max}} \approx 64.625$. The maxima of the twisted-Rastrigin function are correspondingly transformed.

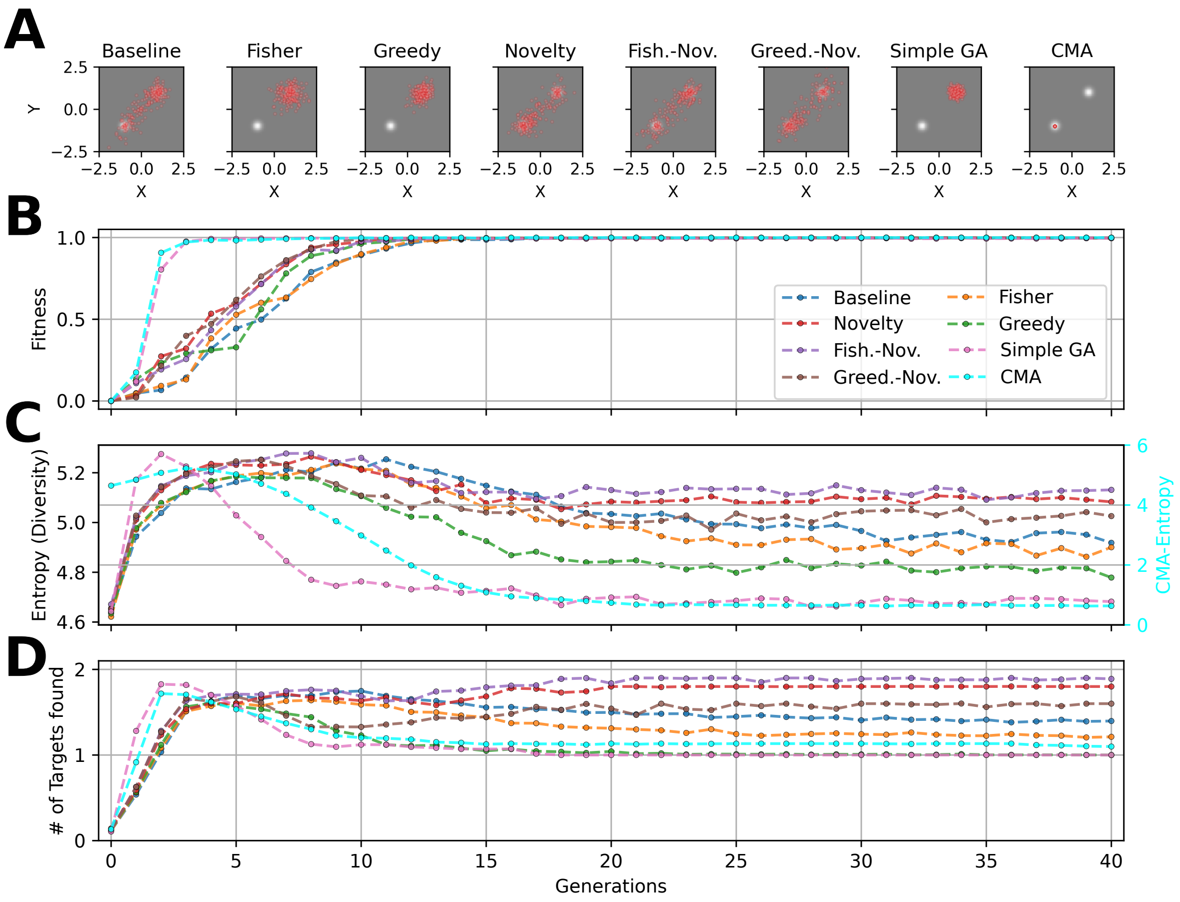

Figure S2: Fitness- and Diversity Benchmarks for the Double-Peak Task. (**A**) Exemplary populations after 40 generations in the 2D double-peak fitness landscape, see eq. (1), for different configurations of the *HADES* and *CHARLES-D* method contrasted with SimpleGA and CMA-ES algorithms (see text). (**B**) Maximum fitness for the different solver configurations illustrated in (A), (**C**) entropy-based diversity (see text) of the population, and (**D**) number of identified targets in the 2D double-peak fitness landscape vs. generations, averaged over 15 statistically independent simulations, respectively. The novelty-conditional *CHARLES-D* method reliably maintains diverse populations of high-quality genetic material, demonstrating that our approach reliably identifies multiple optima through conditional diversification.

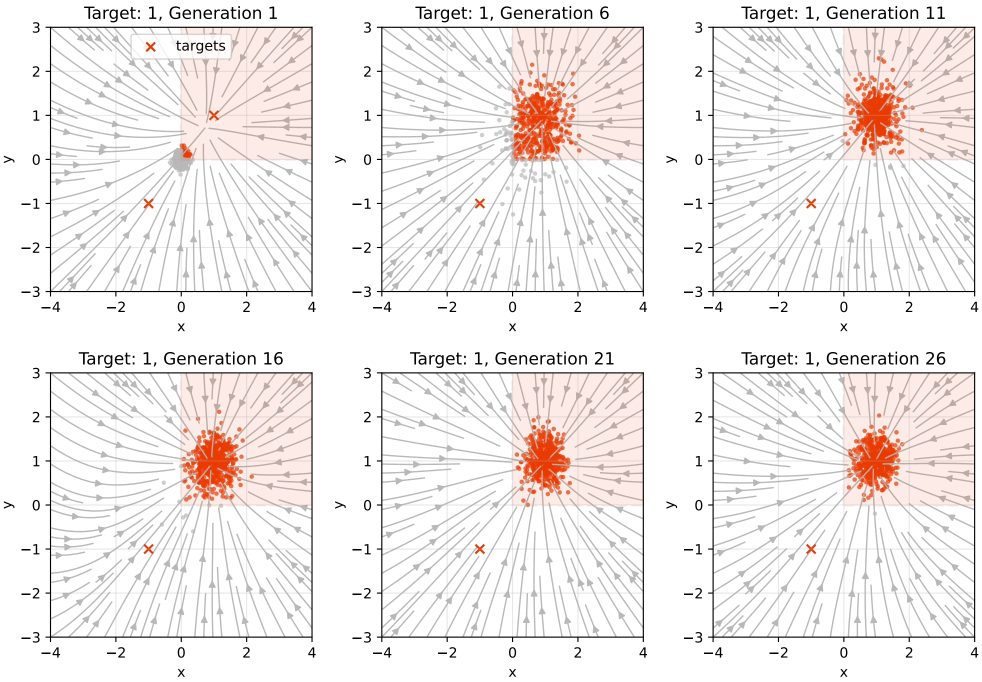

Figure S3: Guiding the search by conditioning on $\boldsymbol{g}_i > 0$. The score function is represented by gray streamlines, and the conditioned region is highlighted by the red box. This condition effectively steers the algorithm to converge on the desired solution within the specified area.

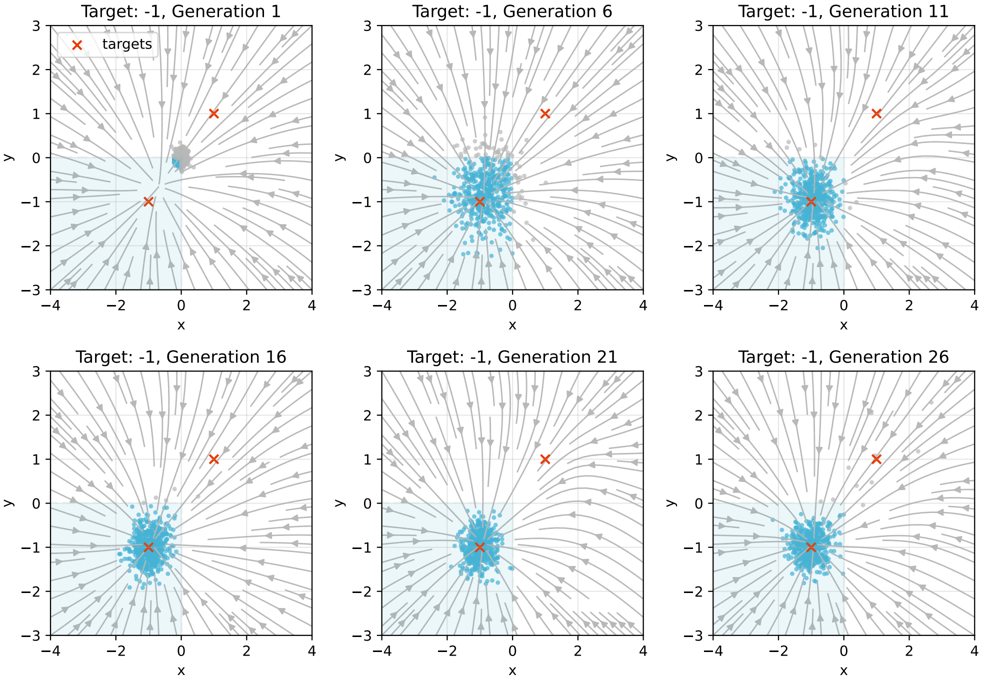

Figure S4: Similar to SI Figure S3, conditioning on $\boldsymbol{g}_i < 0$ guides the algorithm to find the alternative optimal solution.

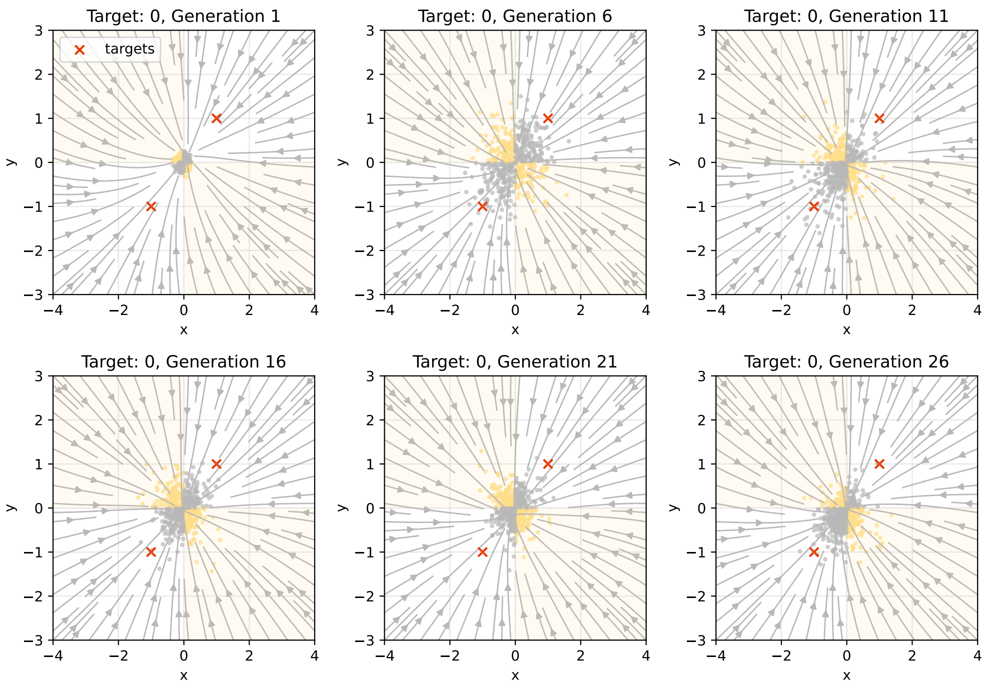

Figure S5: A control experiment in contrast to SI Figures S3 and S4. Here, the conditioned region (yellow box) deliberately contains no optimal solutions. Consequently, the algorithm converges to a suboptimal point, failing to find either target. This result highlights how critical the condition is for the optimization outcome.

S6.4 Grid-Based Entropy Estimate

Similar to Ref. (*45*), we quantify the diversity of the solutions by (i) dividing the 2-D plane into a grid of 101×101 in the range $x_1, x_2 \in [-6, 6]$, and (ii) counting the frequencies of all solutions of a particular generation falling into different grid cells i. The entropy H is then evaluated as

$$H = \sum_{i=1}^{N} P_i \log_2 P_i, \tag{S8}$$

where P_i is the probability of data points being located in grid i. This simple and coarse method allows us to quantify entropy focusing solely on the diversity of solutions across different basins and explicitly avoiding contribution of local diversities.

S6.5 On Evolutionary Redundancy and Sample Novelty from Heuristically Refined Diffusion Models

In all our simulations, the DM is trained on a data buffer consisting of previously sampled genotypes and their associated traits. This might raise the question whether (or to what extend) the DM contributes to the exploration of the parameter space, or merely acts as a post-hoc model that is trained on its own output across generations. Below, we demonstrate that the DM is an integral part in our approach of heuristically exploring parameter landscapes.

To clarify, although we allow explicitly crossover moves and elitism in our algorithm, we did not use these features in the experiments discussed in figs. 2, 4, 6 and 8. In all of those cases, the DM *is* the offspring generator in our evolutionary approach: for every generation, all individuals are sampled directly from the retrained DM, successively extending the training buffer across generations. Thus, the DM naturally implements evolutionary operations in a principled way: fitness-weighted training of the DM corresponds to *selection*, sampling of novel solutions corresponds to *recombination*, while stochasticity during progressive denoising corresponds to *mutation*. In this sense, our method is – by definition – not a post-hoc add-on to traditional evolution but a generative reformulation of its core mechanisms. Furthermore, our conditional evolution capability is a unique benefit of using DMs, which is challenging for other methods.

To isolate the contribution of the DM's generative capabilities to the evolutionary search, we explicitly disabled crossover and elitism in our benchmark experiments figs. 2, 4, 6 and 8, ensuring that each new population originates solely from the DM.

Moreover, our dynamic environment experiments show that DMs can adapt rapidly to shifting optima (see fig. 2) or maintain conditional memory (see fig. 5), capabilities unavailable to restart-based strategies such as multistart CMA-ES (c.f., fig. 6). In the case of fig. 2, we did not use any dataset buffer but retrained the DM on the most recent generation, so the adaptation to the changing environment is solely due to the updated DM.

To further rule out initialization bias in the Rastrigin experiments described in fig. 6, we chose for all solvers an original population with small STD of $\sigma_I = 0.2$ (which does not cover the global optima located at $\approx (\pm 3, \pm 3)$) and deliberately employed a twisted Rastrigin landscape where initial principal directions do not align with the global global optima. Yet, *HADES* and *CHARLES*-

D consistently outperform baselines (see fig. 6), demonstrating genuine algorithmic advantages beyond favorable starts. To emphasize this explorative behavior of our approach, we present the population dynamics when optimizing the Rastrigin (see SI Figure S6 (A)) and twisted Rastrigin tasks (see SI Figure S6 (B)) with *HADES* (c.f., fig. 6): an initial centrally confined population gradually explores higher fitness peaks at larger radii, eventually identifying the global optima in both tasks. This exploration is driven by the refined DM across generations, which we'll discuss below.

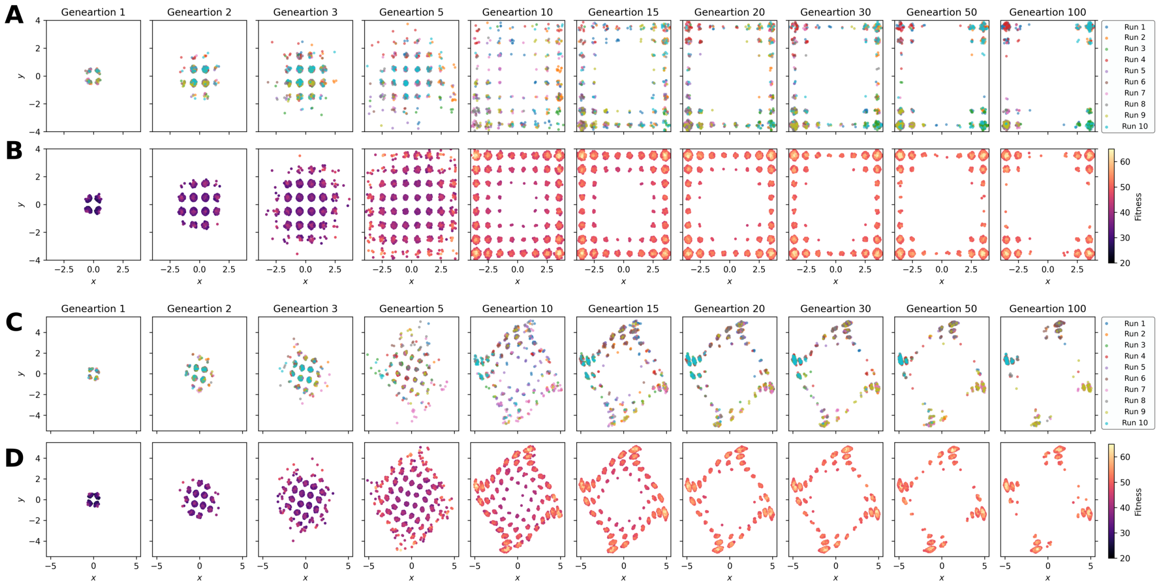

Figure S6: Panel A: the top 25% population across generations (from left to right) of 10 randomly chosen *HADES* optimization runs from fig. 6 (colored by run). Panel B: the top 25% population across generations (from left to right) of all 50 *HADES* optimization runs from fig. 6 (colored by fitness).

We now address an important concern whether the DM might simply restore samples from its training dataset (*i.e.*, from the dataset buffer $\boldsymbol{X}_\tau$ until a certain generation τ) rather than generating novel sampling offspring after retraining. We thus demonstrate in SI Figure S7 – exemplarily on the Rastrigin and twisted Rastrigin tasks – that samples from a generation-by-generation retrained DM $\mathcal{G}_\tau$ exhibit higher fitness and thus drive exploration compared to samples that are (1) directly picked from the DM's training dataset buffer at a specific generation τ, (2) additionally mutated samples from (1), or which are (3) picked via roulette wheel selection (with equivalent parametrization used

in fig. 6 to generate the samples' fitness-weights for DM training for the *HADES* and *CHARLES-D*) from the same buffer as in (1).

More specifically, for case (1) we sample at every generation τ a population of $N_p = 256$ randomly chosen data points from the top 25% of the dataset buffer $\boldsymbol{X}_\tau$; notably – and importantly – this buffer was cumulatively generated by *HADES* up to the previous generation $\tau - 1$, *i.e.*, by sampling from a DM that has been retrained at every prior generation. This thus allows us to compare fitness statistics of either sampling directly from the buffer at a particular generation, or generating samples from a DM that is retrained with exactly the same dataset buffer. In case (2), we mutate 20% (randomly chosen) of the $N_p = 256$ samples from (1) (similar to the SimpleGA in fig. 6 but notably without crossover operations) by adding Gaussian noise of STD 0.2 (which is analogous to the DM's σ_I). In case (3), we use the same buffer as in (1) but select from the buffer according to the roulette wheel selection with equivalent parametrization as used in the *HADES* and *CHARLES-D* optimization in fig. 6.

The results on the example of the Rastrigin (SI Figure S7 (A,B)) and twisted Rastrigin tasks (SI Figure S7 (C,D)) demonstrate that using a constantly refined DM for novel sample-generation in an evolutionary setting increases fitness across generations faster than naïvely sampling and mutating solutions that are present in the very same training dataset buffer of the DM. This is especially illustrative in SI Figure S7 (B,D) when looking at the relative fitness improvement $\Delta f = \max(\boldsymbol{g}_i^{(\mathcal{G}_\tau)} - \boldsymbol{g}_i^{(X\tau)}, 0)$ of DM-sampled individuals, $\boldsymbol{g}_i^{(\mathcal{G}_\tau)}$, or buffer-sampled individuals, $\boldsymbol{g}_i^{(X\tau)}$: before the global maxima are discovered on average (illustrated by the vertical dotted line in SI Figure S7 (B,D)), Δf exhibits positive values for both the mean improved fitness and maximum improved fitness, indicating that DM-sampled individuals exhibit higher fitness on average and absolute values compared to buffered samples.

S7 Cart-Pole Agents

In fig. 7, we utilized different Artificial Neural Network (ANN) architectures to benchmark our algorithms. More specifically, we used multilayer feed-forward (FF), recurrent neural network (RNN) (*54*) , and recurrent gene-regulatory networks (RGRN) (*25*).

For the results depicted in fig. 7 (B), we used RNNs with one to three hidden layers with either

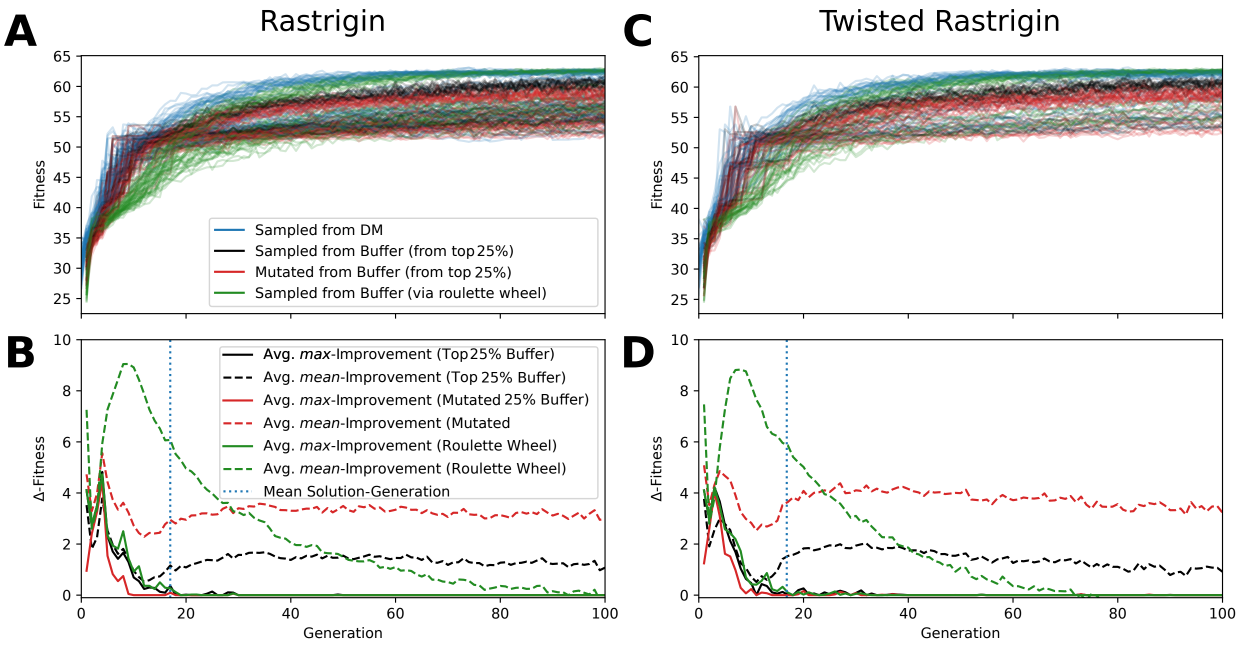

Figure S7: Top panel: The mean fitness of the top 25% population across generations τ for 50 independent lineages solving the Rastrigin (A) and twisted Rastrigin problem (B) with *HADES* optimization (c.f., fig. 6), with candidate solutions sampled from the DM (blue), directly sampled (black) and mutated (red) from the top 25% of solutions present in the dataset buffer (accumulated via *HADES* sampling until the previous generation $\tau - 1$), and sampled via roulette wheel selection from the dataset buffer (green). Bottom panel: fitness improvement $\Delta f = \max(\boldsymbol{g}_i^{(\mathcal{G}_\tau)} - \boldsymbol{g}_i^{(\boldsymbol{X}_\tau)}, 0)$ between DM-sampled parameters $\boldsymbol{g}_i^{(\mathcal{G}_\tau)}$ and buffer sampled parameters $\boldsymbol{g}_i^{(\boldsymbol{X}_\tau)}$ across generations τ; the average improvement of mean top 25% fitness (dashed lines) and maximum fitness (solid line) across 50 independent lineages is presented. The vertical dotted line represents the average number of generations when *HADES* identifies a global maximum peak with $f \geq 64$.

eight or 16 hidden units per hidden layer.

For the results depicted in fig. 7 (C), we used FF agent architectures with one hidden layer with four hidden units and *ReLU* activation function, $f_{\text{ReLU}}(x) = \max(x, 0)$.

For the results depicted in fig. 7 (E), we used an RGRN agent architecture with one hidden layer with a single hidden neuron (see Appendix A in Ref. 25 for details on the architecture).

S8 Cart-Pole Conditioning

In practice, we conditionally train the DM during *CHARLES-D* optimization in fig. 7 jointly on the ANN parameters $\boldsymbol{g}_i$ and the associated resting position $x_i^{(r)}$, the resting velocity $\dot{x}_i^{(r)}$, and the

associated fitness score f_i averaged over $N_e = 16$ episodes. Thus, the vector-valued conditions for given ANN parameters $\boldsymbol{g}_i$ comprises $\boldsymbol{c}_i^{(r)} = \{x_i^{(r)}, \dot{x}_i^{(r)}, f_i\}$.

S9 MountainCar Agent and Conditioning

In fig. 8, we use a recurrent gene-regulatory network (RGRN) as agent controller with one hidden layer with four neuron, amounting to 43 parameters in total (see Appendix A in Ref. 25 for details on the architecture). The neural network parameters $\tilde{\boldsymbol{g}}_i = s_g \boldsymbol{g}_i$ are scaled by a factor of $s_g = \{1, 2, 4, 10\}$ compared to the genotypic parameters $\boldsymbol{g}_i$.

HADES, CMA-ES, and SimpleGA are trained without conditions.

For *HADES* and *CHARLES-D*, we use a six layer DM with 96 neurons per hidden layer and SiLU activation (see SI Section S4).

In practice, we conditionally train the DM during *CHARLES-X* optimization in fig. 8 jointly on the genotypic parameters $\boldsymbol{g}_i$ and the associated horizontal spread Δx_i, and additionally with the associated fitness score f_i for *CHARLES-XD* (see main-text); for all individuals i, Δx_i and f_i are averaged over $N_e = 5$ episodes in all simulations. Thus, the vector-valued conditions for given ANN parameters $\tilde{\boldsymbol{g}}_i$ are given by $c(\boldsymbol{g}_i) = \{\Delta x_i\}$ for *CHARLES-X* and $c(\boldsymbol{g}_i) = \{\Delta x_i, f_i\}$ for *CHARLES-XD*. For offspring generation, we sample the values for the conditional bias from a normal distribution $\boldsymbol{c}^{(\Delta x)} \sim \mathcal{N}(\mu = \max(\{\Delta x_i\}_{\boldsymbol{X}}), \sigma = 0.1)$ centered around the maximum horizontal spread $\max(\{\Delta x_i\}_{\boldsymbol{X}_\tau})$ within the dataset buffer $\boldsymbol{X}_\tau$ at generation τ and an STD of $\sigma = 0.1$. The fitness-conditional sampling of *CHARLES-XD* follows the greedy scheme discussed in the main text.

To match absolute runtime statistics between *HADES* and *CHARLES-D* with CMA-ES and the SimpleGA, we retrained the diffusion model every eigth generation. In SI Figure S8, we present the generational fitness versus total wall-time of the simulations (in seconds) discussed in fig. 8; the simulations are carried out on a single node with 2x AMD 7713 CPUs with 64 cores each (128 cores in total) on the Vienna Scientific Cluster 5 (VSC5) [1], with MPI parallelized fitness evaluation across a population $N_p = 128$ individuals per generation (*i.e.*, one individual per CPU). For algorithmic simplicity, the DM is retrained on a single CPU (MPI rank 0). The average wall-

[1] `https://www.vsc.ac.at`

time per fitness evaluation (*i.e.*, for $N_e = 5$ episodes of the MountainCar environment) are: *HADES* 2.44s; *CHARLES-X* 2.8s; *CHARLES-XD* 2.85s; CMA-ES 2.06s; SimpleGA 2.63s. The results in SI Figure S8 demonstrate that both *HADES* and *CHARLES-X(D)* can perform as efficient as a SimpleGA while resulting in consistently better solutions for $s_g > 1$, and are only slightly slower (in total wall-time) compared to CMA-ES. However, the sample efficiency and training consistency of especially *CHARLES-X* and XD are far superior to CMA-ES, as illustrated by the vanishing median fitness values of the latter (*i.e.*, most CMA-ES runs don't find the solution at all in 500 generations).

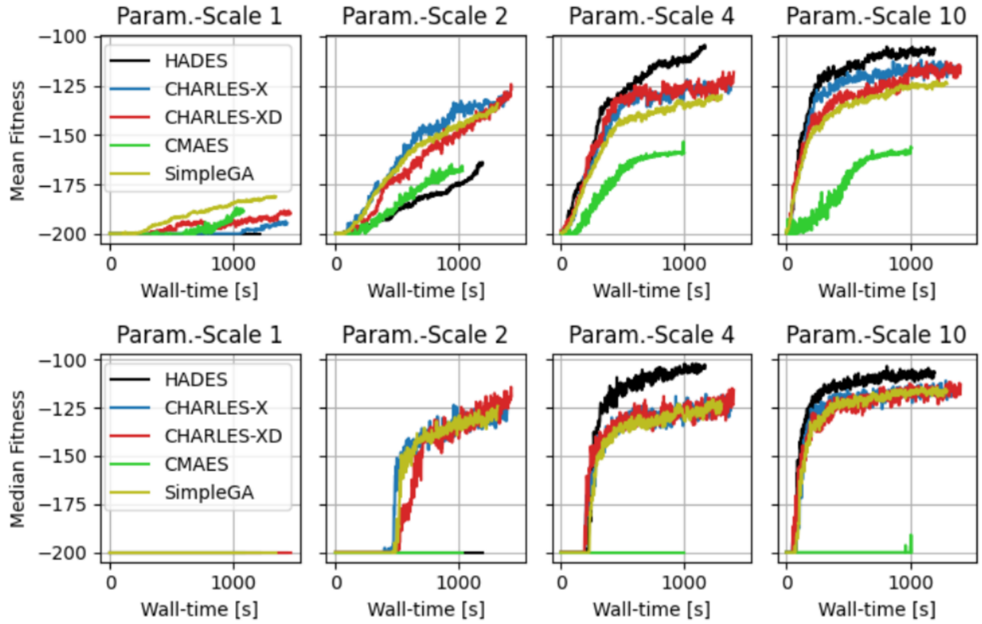

Figure S8: Mean (top row) and median (bottom row) fitness vs. wall-time (in seconds) of solving the MoutnainCar-v0 environment with parameter scaling factors $s_g = \{1, 2, 4, 10\}$ (columns); same simulation data as in fig. 8. The simulations are carried out on a single node with 2x AMD 7713 CPUs with 64 cores each (128 cores in total) on the VSC5, with MPI parallelized fitness evaluation across a population $N_p = 128$ individuals per generation (*i.e.*, one individual per CPU). For algorithmic simplicity, the DM is retrained on a single CPU (MPI rank 0).

S10 Lunar-Lander Conditioning

Here, we apply *HADES* and *CHARLES-D* to the lunar-lander (*55*) (v3) reinforcement learning (RL) environment, a considerably more involved problem compared to the cart-pole task. The lunar-

lander environment is a rocket trajectory optimization problem in two dimensions (in horizontal and vertical directions x, y): an agent can control the rocket by applying thrusters in vertical and lateral directions in order to land safely on a predefined landing pad, designated by two yellow flags (c.f., SI Figure S9(A,B) for an illustration).

The observation-space is eight-dimensional, and comprises the x- and y-coordinates of the rocket, its linear velocities, $\dot{x}$ and $\dot{y}$, the rocket's angle ϕ and angular velocity $\dot{\phi}$, and two boolean variables l_1 and l_2 specifying wether one of the two legs has ground contact. The action-space for the here used "continuous" lunar-lander environment is two-dimensional: while the first coordinate determines the throttle of the main engine, the second coordinate specifies the throttle of the lateral boosters.

The reward comprises several factors concerning the distance of the rocket to the landing pad (located at coordinates $\mathbf{x}_0 = (0, 0)$), the rockets speed and tilt, fuel usage of vertical and horizontal stabilization, and landing (or crashing) skills: In more detail, the reward is increased/decreased if the rocket's distance to the landing pad becomes smaller/larger, whether the rocket moves slower/faster, and the rocket's tilt is more/less upright; furthermore, the reward is increased by 10 points for each leg touching the ground, decreased by 0.03 points for each time-step the side-engine is fired, and decreased by 0.3 points for each time-step the main-engine is fired. An additional 100 points are received if the landing was safely done, and a pentalty of -100 points is received upon crashing. An episode is considered successful if the cumulative reward is at least 200 points.

In SI Figure S9(C), we demonstrate that both *HADES* and Fisher-type *CHARLES-D* (c.f., fig. 7) can solve the environment exceptionally fast, *i.e.*, in ≈ 5 generations in the best case, and $\gtrapprox 20$ generations on average. We used feed-forward (FF) architectures for the controlling neural network, scanning all combinations of $\{1, 2, 3\}$ hidden layers with a total number of $\{8, 16, 32\}$ "Tanh"-activated neurons per layer, utilized for every combination a relatively simple 4-layer FF diffusion model with 32 "ReLU" activated units, and chose the remaining parameters analogous to fig. 7; in SI Figure S10, we present the *HADES* training results for different recurrent (RNN) controller architectures, demonstrating that even much more complex agent architectures can be successfully trained with our method.

An interesting local minimum of the lunar-lander environment is given by a situation where the rocket successfully lands on the landing pad, but does not turn off the side boosters (since

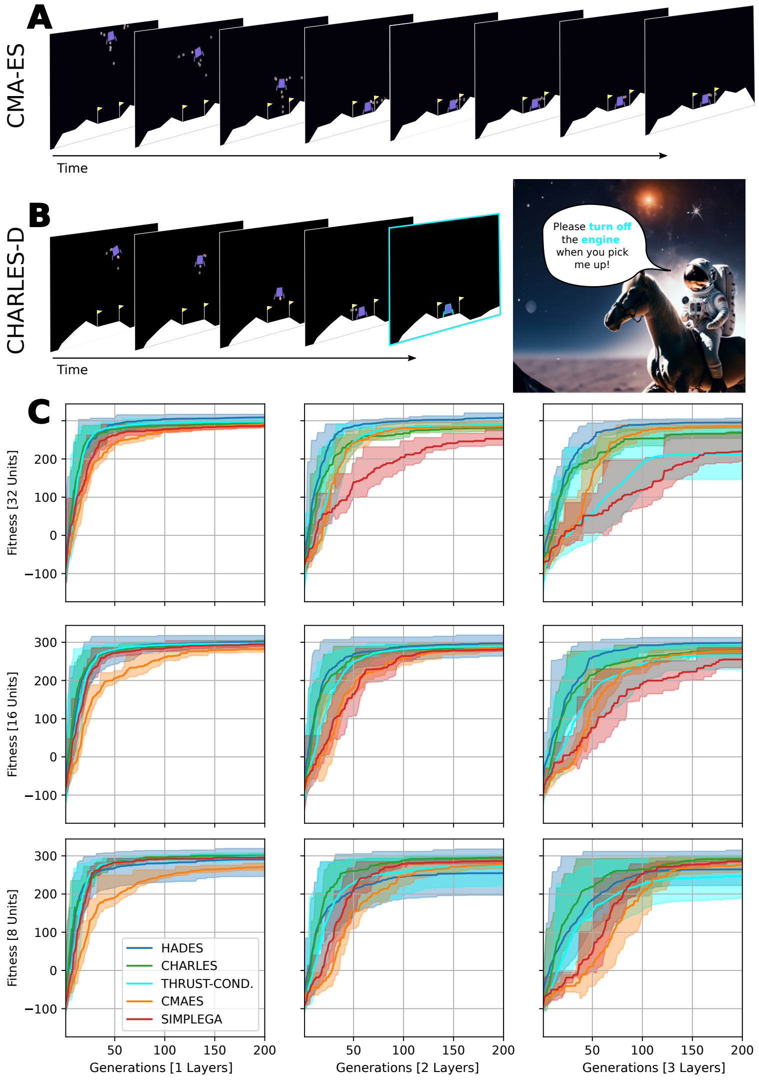

Figure S9: Training a lunar-lander agent to land safely at the center of a landing pad, "throttle-conditional" to exhibit efficient fuel-usage. (A) An example trajectory of a lunar-lander rocket trained with CMA-ES; the agent lands successfully but demonstrates sub-optimal behavior after landing by constantly activating the side-engines (illustrated by red circles streaming out of the violet rocket). (B) An example trajectory of a lunar-lander rocked trained with *CHARLES-D* to explicitly minimize throttle after landing via "throttle-conditional" sampling, resulting in quicker episode-terminations, and more efficient fuel-usage. (C) Evolutionary training progress of the *HADES*, Fisher-type *CHARLES-D*, and "throttle-conditional" *CHARLES-D* (c.f., panel (B)), CMA-ES, and SimpleGA training for different feed-forward (FF) agent architectures (see axes labels and text) for 200 generations, with elite fitness-scores averaged over 10 statistically independent solver evaluations each; the problem is considered solved if a fitness-score of > 200 is reached (c.f., magenta marking).

the side booster penalty is very low, *i.e.*, -0.03 per time frame). In SI Figure S9(A), we present a typical rocket trajectory of a CMA-ES (*39*) trained agent falling into that category: the agent can successfully land, but then constantly utilizes the side boosters to minimize the distance to the origin, $\mathbf{x}_0$, of the landing pad. The episode won't stop until the thrusters are turned off successfully, or the maximum number of time-steps per episode is reached. Thus, such an agent causes artificially prolonged episode duration, as illustrated in SI Figure S9(A).

Despite solving the problem according to the target reward-score (> 200), this is an unsatisfactory situation considering fuel-usage with potentially detrimental implications for the ground-crew. With our *CHARLES-D* framework, we can use conditional sampling to evolve solutions that explicitly turn off the engine when the rocket's legs have ground contact, again without modifying the reward or fitness-score of the problem: here, we simply count all time frames l_c when either one of the legs have ground contact, *i.e.*, when either l_1 or l_2 give a "true" signal. We use the mean ground-contact duration of a particular agent (averaged over 8 episodes) as condition $\boldsymbol{c}^l = l_c$, and jointly train the diffusion model to associate these conditions with the corresponding agent parameters $\boldsymbol{g}_i$ giving rise to that behavior. During sampling, we then use the target condition $\boldsymbol{c}^{(\mathrm{T})} = 0$ to generate high-fitness agents that are biased towards minimal ground contact time.

SI Figure S9(B) illustrates a trajectory of an agent trained with such "throttle-conditioning" via *CHARLES-D*, and SI Figure S9(C) and SI Figure S10 contain quantitative results about the training behavior for FF and RNN architectures, respectively. While both *HADES* and *CHARLES-D* demonstrate strong performance in evolving successfully rocket policies, conditional sampling can help in training progress, especially for small- and medium-sized controller architectures. However, increasingly complex agent architectures may require larger diffusion-models to capture both the dynamics and the throttle condition.

References and Notes

1. D. P. Kingma, M. Welling, Auto-Encoding Variational Bayes, in *2nd International Conference on Learning Representations, ICLR 2014, Banff, AB, Canada, April 14-16, 2014, Conference Track Proceedings*, Y. Bengio, Y. LeCun, Eds. (2014), http://arxiv.org/abs/1312.6114.

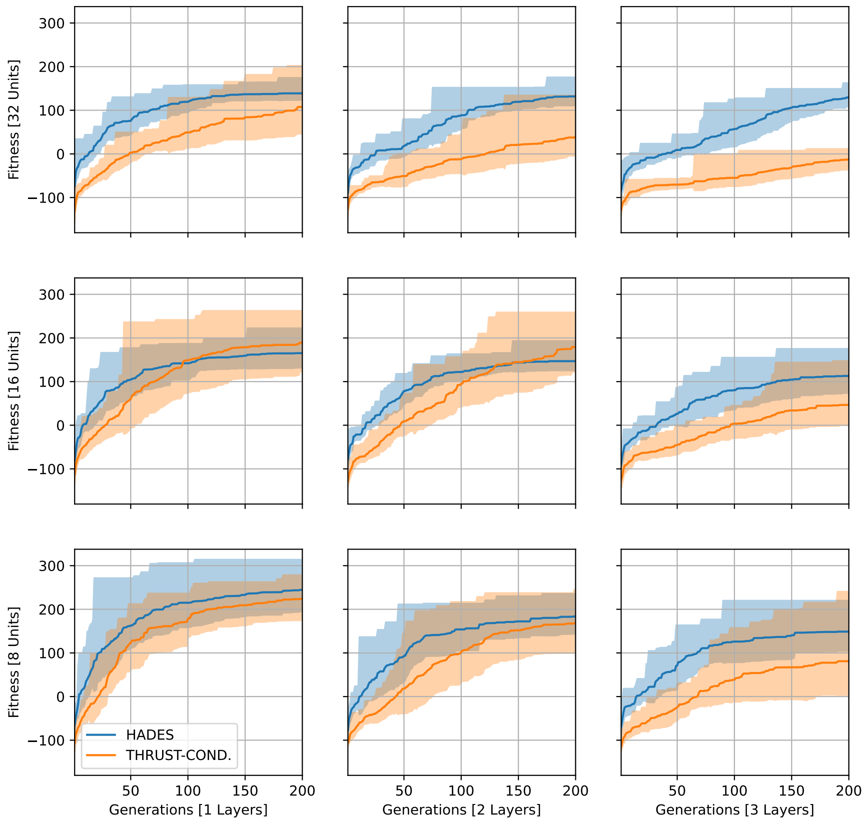

Figure S10: Same as SI Figure S9(C), but with recurrent neural network (RNN) controller architectures, instead of feed-forward controllers. We present results from *HADES* and "throttle-conditional" *CHARLES-D* for 200 generations, with elite fitness-scores averaged over 10 independent evolutionary runs each.

2. A. Gaier, A. Asteroth, J.-B. Mouret, Discovering representations for black-box optimization, in *Proceedings of the 2020 Genetic and Evolutionary Computation Conference* (2020), pp. 103–111.

3. P. J. Bentley, S. L. Lim, A. Gaier, L. Tran, Evolving through the looking glass: Learning improved search spaces with variational autoencoders, in *International Conference on Parallel Problem Solving from Nature* (Springer) (2022), pp. 371–384.

4. J. Caldwell, J. Knowles, C. Thies, F. Kubacki, R. Watson, Deep Optimisation: Transitioning the Scale of Evolutionary Search by Inducing and Searching in Deep Representations. *SN Computer Science* **3** (3) (2022), doi:10.1007/s42979-022-01109-w, `http://dx.doi.org/10.1007/s42979-022-01109-w`.

5. M. Levin, Darwin's agential materials: evolutionary implications of multiscale competency in developmental biology. *Cellular and Molecular Life Sciences* **80** (6), 142 (2023).

6. R. A. Watson, M. Levin, C. L. Buckley, Design for an individual: connectionist approaches to the evolutionary transitions in individuality. *Frontiers in Ecology and Evolution* **10**, 823588 (2022).

7. R. A. Watson, *et al.*, Evolutionary connectionism: algorithmic principles underlying the evolution of biological organisation in evo-devo, evo-eco and evolutionary transitions. *Evolutionary biology* **43**, 553–581 (2016).

8. S. A. Frank, Evolutionary design of regulatory control. II. Robust error-correcting feedback increases genetic and phenotypic variability. *Journal of Theoretical Biology* **468**, 72–81 (2019), doi:10.1016/j.jtbi.2019.02.012, `http://dx.doi.org/10.1016/j.jtbi.2019.02.012`.

9. S. A. Frank, Measurement invariance explains the universal law of generalization for psychological perception. *Proceedings of the National Academy of Sciences* **115** (39), 9803–9806 (2018), doi:10.1073/pnas.1809787115, `https://www.pnas.org/doi/abs/10.1073/pnas.1809787115`.

10. S. A. Frank, Evolution of Robustness and Cellular Stochasticity of Gene Expression. *PLoS Biology* **11** (6), e1001578 (2013), doi:10.1371/journal.pbio.1001578, http://dx.doi.org/10.1371/journal.pbio.1001578.

11. S. A. Frank, Maladaptation and the Paradox of Robustness in Evolution. *PLoS ONE* **2** (10), e1021 (2007), doi:10.1371/journal.pone.0001021, http://dx.doi.org/10.1371/journal.pone.0001021.

12. A. Wagner, W. Rosen, Spaces of the possible: universal Darwinism and the wall between technological and biological innovation. *Journal of The Royal Society Interface* **11** (97), 20131190 (2014), doi:10.1098/rsif.2013.1190, https://royalsocietypublishing.org/doi/abs/10.1098/rsif.2013.1190.

13. A. Wagner, The molecular origins of evolutionary innovations. *Trends in Genetics* **27** (10), 397–410 (2011), doi:10.1016/j.tig.2011.06.002, http://dx.doi.org/10.1016/j.tig.2011.06.002.

14. K. H. Ten Tusscher, P. Hogeweg, Evolution of Networks for Body Plan Patterning; Interplay of Modularity, Robustness and Evolvability. *PLoS Computational Biology* **7** (10), e1002208 (2011), doi:10.1371/journal.pcbi.1002208, http://dx.doi.org/10.1371/journal.pcbi.1002208.

15. G. P. Wagner, L. Altenberg, Perspective: Complex Adaptations and the Evolution of Evolvability. *Evolution* **50** (3), 967–976 (1996), http://www.jstor.org/stable/2410639.

16. J. Lehman, K. O. Stanley, Abandoning objectives: Evolution through the search for novelty alone. *Evolutionary computation* **19** (2), 189–223 (2011).

17. J. K. Pugh, L. B. Soros, K. O. Stanley, Quality Diversity: A New Frontier for Evolutionary Computation. *Frontiers in Robotics and AI* **3** (2016), doi:10.3389/frobt.2016.00040, https://www.frontiersin.org/journals/robotics-and-ai/articles/10.3389/frobt.2016.00040.

18. B. Hartl, M. Levin, What does evolution make? Learning in living lineages and machines. *Trends in Genetics* **41** (6), 480–496 (2025), doi:10.1016/j.tig.2025.04.002, https://doi.org/10.1016/j.tig.2025.04.002.

19. K. J. Mitchell, N. Cheney, The Genomic Code: the genome instantiates a generative model of the organism. *Trends in Genetics* **41** (6), 462–479 (2025), doi:10.1016/j.tig.2025.01.008, http://dx.doi.org/10.1016/j.tig.2025.01.008.

20. G. Pezzulo, M. Levin, Top-down models in biology: explanation and control of complex living systems above the molecular level. *Journal of The Royal Society Interface* **13**, 20160555 (2016), doi:10.1098/rsif.2016.0555.

21. K. Kouvaris, J. Clune, L. Kounios, M. Brede, R. A. Watson, How evolution learns to generalise: Using the principles of learning theory to understand the evolution of developmental organisation. *PLoS computational biology* **13** (4), e1005358 (2017).

22. R. A. Watson, E. Szathmáry, How can evolution learn? *Trends in ecology & evolution* **31** (2), 147–157 (2016).

23. D. A. Power, *et al.*, What can ecosystems learn? Expanding evolutionary ecology with learning theory. *Biology direct* **10**, 1–24 (2015).

24. R. A. Watson, G. P. Wagner, M. Pavlicev, D. M. Weinreich, R. Mills, THE EVOLUTION OF PHENOTYPIC CORRELATIONS AND "DEVELOPMENTAL MEMORY". *Evolution* **68** (4), 1124–1138 (2014), doi:10.1111/evo.12337, https://doi.org/10.1111/evo.12337.

25. B. Hartl, S. Risi, M. Levin, Evolutionary Implications of Self-Assembling Cybernetic Materials with Collective Problem-Solving Intelligence at Multiple Scales. *Entropy* **26** (7), 532 (2024).

26. A. Mordvintsev, E. Randazzo, C. Fouts, Growing Isotropic Neural Cellular Automata, in *The 2022 Conference on Artificial Life*, ALIFE 2022 (MIT Press) (2022), doi:10.1162/isal_a_00552, http://dx.doi.org/10.1162/isal_a_00552.

27. A. Mordvintsev, E. Randazzo, E. Niklasson, M. Levin, Growing Neural Cellular Automata. *Distill* **5** (2) (2020), doi:10.23915/distill.00023, https://doi.org/10.23915/distill.00023.

28. X. Li, A. G.-O. Yeh, Neural-network-based cellular automata for simulating multiple land use changes using GIS. *Int. J. Geogr. Inf. Sci.* **16** (4), 323–343 (2002), doi:10.1080/13658810210137004, https://doi.org/10.1080/13658810210137004.

29. C. G. Langton, *Artificial life: An overview* (Mit Press) (1997).

30. G. P. Wagner, M. Pavlicev, J. M. Cheverud, The road to modularity. *Nature Reviews Genetics* **8** (12), 921–931 (2007), doi:10.1038/nrg2267, http://dx.doi.org/10.1038/nrg2267.

31. G. Schlosser, G. P. Wagner, *Modularity in Development and Evolution* (University of Chicago Press, Chicago, IL) (2004).

32. R. Calabretta, A. D. Ferdinando, G. P. Wagner, D. Parisi, What does it take to evolve behaviorally complex organisms? *Biosystems* **69** (2), 245–262 (2003), doi:https://doi.org/10.1016/S0303-2647(02)00140-5, https://www.sciencedirect.com/science/article/pii/S0303264702001405.

33. K. O. Stanley, R. Miikkulainen, Evolving Neural Networks through Augmenting Topologies. *Evol. Comput.* **10** (2), 99–127 (2002), doi:10.1162/106365602320169811, https://doi.org/10.1162/106365602320169811.

34. E. Najarro, S. Sudhakaran, C. Glanois, S. Risi, HyperNCA: Growing Developmental Networks with Neural Cellular Automata, in *From Cells to Societies: Collective Learning across Scales* (2022), https://openreview.net/forum?id=H5eErMka-9.

35. E. Najarro, S. Sudhakaran, S. Risi, Towards self-assembling artificial neural networks through neural developmental programs, in *Artificial Life Conference Proceedings 35* (MIT Press One Rogers Street, Cambridge, MA 02142-1209, USA journals-info . . .), vol. 1 (2023), p. 80.

36. O. Chang, H. Lipson, Neural Network Quine, in *2018 Conference on Artificial Life, ALIFE 2018, Tokyo, Japan, July 23-27, 2018*, T. Ikegami, *et al.*, Eds. (MIT Press) (2018), pp. 234–241, doi:10.1162/ISAL_A_00049, https://doi.org/10.1162/isal_a_00049.

37. V. N. Premakumar, *et al.*, Unexpected Benefits of Self-Modeling in Neural Systems (2024), `https://arxiv.org/abs/2407.10188`.

38. J. C. Zagal, H. Lipson, Towards self-reflecting machines: Two-minds in one robot, in *Advances in Artificial Life. Darwin Meets von Neumann: 10th European Conference, ECAL 2009, Budapest, Hungary, September 13-16, 2009, Revised Selected Papers, Part I 10* (Springer) (2011), pp. 156–164.

39. N. Hansen, A. Ostermeier, Completely derandomized self-adaptation in evolution strategies. *Evolutionary computation* **9** (2), 159–195 (2001).

40. F. Sehnke, *et al.*, Parameter-exploring policy gradients. *Neural Networks* **23** (4), 551–559 (2010).

41. P. A. Vikhar, Evolutionary algorithms: A critical review and its future prospects, in *2016 International conference on global trends in signal processing, information computing and communication (ICGTSPICC)* (IEEE) (2016), pp. 261–265.

42. J. J. Grefenstette, Genetic algorithms and machine learning, in *Proceedings of the sixth annual conference on Computational learning theory* (1993), pp. 3–4.

43. D. E. Golberg, Genetic algorithms in search, optimization, and machine learning. *Addion wesley* **1989** (102), 36 (1989).

44. J. H. Holland, *Adaptation in natural and artificial systems: an introductory analysis with applications to biology, control, and artificial intelligence* (MIT press) (1992).

45. Y. Zhang, B. Hartl, H. Hazan, M. Levin, Diffusion Models are Evolutionary Algorithms, in *The Thirteenth International Conference on Learning Representations, ICLR 2025, Singapore, April 24-28, 2025* (OpenReview.net) (2025), `https://openreview.net/forum?id=xVefsBbG2O`.

46. J. Song, C. Meng, S. Ermon, Denoising Diffusion Implicit Models, in *International Conference on Learning Representations* (2021), `https://openreview.net/forum?id=St1giarCHLP`.

47. A. Lugmayr, *et al.*, RePaint: Inpainting using Denoising Diffusion Probabilistic Models, in *2022 IEEE/CVF Conference on Computer Vision and Pattern Recognition (CVPR)* (2022), pp. 11451–11461, doi:10.1109/CVPR52688.2022.01117.

48. J. Ho, T. Salimans, Classifier-Free Diffusion Guidance, in *NeurIPS 2021 Workshop on Deep Generative Models and Downstream Applications* (2021), https://openreview.net/forum?id=qw8AKxfYbI.

49. A. Y. Ng, D. Harada, S. J. Russell, Policy Invariance Under Reward Transformations: Theory and Application to Reward Shaping, in *Proceedings of the Sixteenth International Conference on Machine Learning*, ICML '99 (Morgan Kaufmann Publishers Inc., San Francisco, CA, USA) (1999), p. 278–287.

50. L. Ambrogioni, In Search of Dispersed Memories: Generative Diffusion Models Are Associative Memory Networks. *Entropy* **26** (5), 381 (2024), doi:10.3390/E26050381, https://doi.org/10.3390/e26050381.

51. R. Rombach, A. Blattmann, D. Lorenz, P. Esser, B. Ommer, High-resolution image synthesis with latent diffusion models, in *Proceedings of the IEEE/CVF conference on computer vision and pattern recognition* (2022), pp. 10684–10695.

52. T. Brooks, *et al.*, Video generation models as world simulators (2024).

53. D. Lombardi, S. Pant, Nonparametric k-nearest-neighbor entropy estimator. *Phys. Rev. E* **93**, 013310 (2016), doi:10.1103/PhysRevE.93.013310, https://link.aps.org/doi/10.1103/PhysRevE.93.013310.

54. D. E. Rumelhart, G. E. Hinton, R. J. Williams, *Learning internal representations by error propagation* (MIT Press, Cambridge, MA, USA), p. 318–362 (1986).

55. G. Brockman, *et al.*, OpenAI Gym (2016), https://arxiv.org/abs/1606.01540.
